# Nutritional, Antioxidant, Antimicrobial, and Anticholinesterase Properties of *Phyllanthus emblica*: A Study Supported by Spectroscopic and Computational Investigations

**DOI:** 10.3390/metabo13091013

**Published:** 2023-09-14

**Authors:** Mohamed A. A. Orabi, Aso Hameed Hasan, Sameh F. AbouZid, Dalia El Amir, Mona H. Hetta, Ahmed Abdullah Al Awadh, Omaish Salman Alqahtani, Tsutomu Hatano, Mohamed A. El-Shanawany

**Affiliations:** 1 Department of Pharmacognosy, College of Pharmacy, Najran University, Najran 66454, Saudi Arabia; osalqahtani@nu.edu.sa; 2 Department of Chemistry, College of Science, University of Garmian, Kalar 46021, Kurdistan Region, Iraq; aso.hameed@garmian.edu.krd; 3 Department of Chemistry, Faculty of Science, Universiti Teknologi Malaysia, Johor Bahru 81310, Johor, Malaysia; 4 Department of Pharmacognosy, Faculty of Pharmacy, Beni-Suef University, Beni-Suef 62514, Egypt; sameh.zaid@pharm.bsu.edu.eg; 5 Department of Pharmacognosy, Faculty of Pharmacy, Fayoum University, Fayoum 63514, Egypt; monahetta@gmail.com; 6 Department of Clinical Laboratory Sciences, Faculty of Applied Medical Sciences, Najran University, Najran 66454, Saudi Arabia; aaalawadh@nu.edu.sa; 7 Graduate School of Medicine, Dentistry and Pharmaceutical Sciences, Okayama University, Tsushima, Okayama 700-8530, Japan; hatano-t@cc.okayama-u.ac.jp; 8 Department of Pharmacognosy, Faculty of Pharmacy, Badr University in Cairo (BUC), Badr City 11829, Egypt; mohamed.elshanawani@pharm.aun.edu.eg

**Keywords:** antioxidant, antimicrobial, antiacetylcholinesterase, *Phyllanthus emblica*, polyphenols, nutrition, molecular docking

## Abstract

Dietary fruits and vegetables play a vital role as food and drugs and are the main sources of antioxidant defences against degenerative diseases, such as brain dysfunctions, cardiovascular diseases, immune system deteriorations, and cancers, brought on by oxidative damage. *Phyllanthus emblica* is a significant herbal remedy used in conventional medicine to recover lost strength and power. In this research, the potential value of *Phyllanthus emblica* as a food and drug is researched. The total phenolic, total flavonoid, and total tannin contents as well as the nutritional value, vitamin C, vitamin E, and mineral contents of different organs of *P. emblica* were evaluated. The antioxidant and antimicrobial activities of extracts and fractions of different organs of *P. emblica* were determined. A total of eleven flavonoids, simple phenolic, tannin-related phenolic, and tannin molecules were isolated from a hydroalcoholic extract of the leaves and fruits. The structures were identified by spectroscopic data and comparison with the literature values as gallic acid (**1**), naringenin 7-*O*-(6″-*O*-galloyl)-*β*-D-glucopyranoside (**2**), 3,3′-di-*O*-methyl ellagic acid-4′-*O*-*β*-d-glucopyranoside (**3**), 1-*O*-galloyl glycerol (**4**), 1,6-di-*O*-galloyl-*β*-d-glucopyranoside (**5**), flavogallonic acid bislactone (**6**), corilagin (**7**), ethyl gallate (**8**), urolithin M5 (**9**), (E)-*p*-coumaroyl-1-*O*-*β*-d-glucopyranoside (**10**), and 1,2,4,6-tetra-*O*-galloyl-*β*-d-glucopyranoside (**11**). Among them, compounds **3** and **10** are first isolated from the plant. Molecular docking was performed to investigate the comparative interactions between positive controls (galantamine and donepezil) and selected compounds utilizing acetylcholinesterase (4EY7) as a target receptor. Results exhibited the potency of these compounds against the target receptor. In summary, *P. emblica* has a wealth of minerals, vitamins C and E, and polyphenolic phytochemicals that may work together to treat infectious disease, prevent and/or treat oxidative-damage-related illnesses including Alzheimer’s disease.

## 1. Introduction

Environmental radiation and the oxidant byproducts of regular metabolism seriously damage the DNA, protein, and lipid biomolecules. This damage plays a significant role in the development of degenerative illnesses such as brain dysfunctions, cardiovascular diseases, immune system deterioration, and cancers. Millions of people worldwide are now affected by neurological diseases such as Alzheimer’s and dementia. Even though there are treatments, they rarely provide the intended therapeutic effects. As a result, many research teams have been trying to come up with an innovative approach for treating these ailments. Dietary fruits and vegetables play a vital role as food and drugs and are the main sources of antioxidant defenses against these damages. *Phyllanthus emblica* L. (Phyllanthaceae), also known as Amla, Amlaki, or Indian Gooseberry, is widely distributed throughout tropical and subtropical regions including Pakistan, Uzbekistan, Sri Lanka, Southeast Asia, China, and Malaysia [1]. It is native to India and has a hallowed position in Ayurveda; all parts of the plant are used in herbal remedies to restore lost strength and vitality and as medicine [2,3]. *P. emblica* is an important source of vitamin C, amino acids, and minerals [4]. It produces tannins, curcuminoids, flavonoids, triterpenes, fatty acids, and sterols as the main plant constituents [5]. It sometimes acts as a cardiac stimulant and lowers cholesterol levels [6]. Amla fruits are a wonderful tonic for strengthening and nourishing the lungs. Fruits with seeds are used for asthma, bronchitis, and biliousness. Furthermore, fruit is a gentle laxative, anti-oxidant, anti-aging, and anti-tumor. The therapeutic effects of *P. emblica*, which include antioxidant, anti-inflammatory, anti-hyperlipidemic, anti-diabetic, and neuroprotective activity, have been investigated as well [7,8,9,10,11,12]. The aqueous extract of *P. emblica* inhibited the growth of variant cancer cell lines, A549 (lung), HepG2 (liver), HeLa (cervical), MDA-MB-231 (breast), SK-OV3 (ovarian), and SW620 (colorectal) [13,14,15,16].

Due to its medicinal value, the phenolics, flavonoids, vitamin C, vitamin E, and some mineral contents of leaves, fruits, and stem branches of *P. emblica* L. cultivated in Egypt are evaluated in this research. Chromatographic isolation and spectroscopic identification of various Amla leaves and fruit constituents are also reported. In addition, the antioxidant and antimicrobial properties of solvent extracts and fractions of various organs of *P. emblica* L. were determined.

Today, one of the proposed preventive effects associated with the bioactive compounds in Amla is the reduction of neurological abnormalities, particularly the biochemical changes seen in carriers of Alzheimer’s disease [9]. Therefore, the possible exploitation of the isolated compounds as anti-Alzheimer’s disease operating by inhibition of acetylcholinesterase was evaluated by computational molecular docking.

## 2. Materials and Methods

### 2.1. General Experimental Procedures

UV spectra were recorded using Shimadzu UV–Viz, 1601 PC ultraviolet spectrometer (Shimadzu, Kyoto, Japan). For vitamin C determination, a Jupiter C-18 HPLC column (Phenomenex security system), a Jasco 870-UV detector (Jasco spectroscopic, Tokyo, Japan), a guard cell (Model 5020 operating at ±200 mV), a Waters 6000 A eluent pump (Waters, Milford, OH, USA), a Waters Wisp 710 B refrigerated autosampler coupled to the eluent pump, a reaction coil for mixing Dionex RP-1 pump with mobile phase, an ultracentrifuge, and a 0.45 μm membrane filter were used. For vitamin E determination, analytical Nova Pak C-18 HPLC columns containing C-18 pellicular packing material (degrade themselves 30–40 μm), an alliance separation module (model 2690, Waters), and a Waters 996 Photodiode array detector (PDA), solvent delivery pump (515; Waters, Milford, OH, USA) were used. For quantitative determination of minerals, a Perkin–Elmer, Optima 2000 DV ICP-MS system, Australia, was used for the simultaneous multi-element detection of minerals, and a centrifuge was used to separate undissolved parts of the plant samples. MLTW 54 (Germany), a microwave oven equipped with polytetrafluoroethylene (PTFE) vessels, Bergh of Speed wave MWS, Germany, was used for sample digestion.

^1^H and ^13^C NMR spectra as well as 2D spectra were recorded on NMR Varian Inova AS 600 (Varian) operating at 600 MHz for ^1^H and 151 MHz for ^13^C and NMR Bruker operating at 400 MHz for ^1^H and 100 MHz for ^13^C using CDCl_3_, CD_3_OD, DMSO-*d*_6_ as solvents and the solvent peaks as internal references. Chemical shifts were recorded in ppm on *δ* scale while the coupling constant (*J*) values were estimated in Hertz (Hz). Electrospray ionization mass (ESIMS) data were acquired on an API-4000 instrument (AB Sciex, Framingham, MA, USA) using CH_3_CN/H_2_O (1:1, *v*/*v*) solvent. Electron impact (EI)MS was acquired on a JEOL mass spectrometer (70 eV). MCI-gel CHP-20P (Mitsubishi Chemical, Tokyo, Japan) and Sephadex LH-20 (GE Healthcare Bio-Science AB, Uppsala, Sweden) were used for column chromatography. RP-HPLC analysis was performed at 40 °C on a YMC-Pack ODS-A A-303 (YMC, Kyoto, Japan) column (4.6 i.d. × 250 mm), using the solvent gradient indicated in the extraction isolation below. Preparative RP-HPLC was performed at 40 °C on a YMC-Pack ODS-A A-324 column (10 i.d. × 300 mm) using the solvent systems as in the extraction isolation section below. TLC was performed on pre-coated silica gel 60 GF254 TLC plates (Fluka, Neu-Ulm, Germany) with visualization by spraying with *p*-Anisaldehyde-Sulphuric acid spray reagent. Preparative HPLC was performed on YMC-Pack ODS-A A-324 column (10 i.d. × 300 mm) (YMC, Kyoto, Japan). Antimicrobial screening was performed on Trypticase soy agar medium (Oxoid, Hampshire, UK) composed of casein hydrolysate (17 g/L, Soya peptone (3 g/L), sodium chloride (5 g/L), potassium phosphate (2.5 g/L), glucose (2.5 g/L), and agar (17g/L).

### 2.2. Plant Material

Samples of *Phyllanthus emblica* L. used in this study were collected during the fruiting stage of plants growing in El Qanater gardens, Qaliubia Governorate, Egypt (usually in October and September of these years), and identified by Dr. Abd El Halim Mohamed Senior plant taxonomist, Flora and Phytotaxonomy Department, Agricultural Research Center, Dokki, Giza, and by Mrs. Therese Labib, Head Specialist for Plant Identification Unit, El-Orman Garden, Cairo, Egypt. A voucher specimen (No. BUPD-38) was deposited in the Herbarium of Pharmacognosy Department, Faculty of Pharmacy, Beni-Suef University, Beni-Suef, Egypt.

### 2.3. Extraction and Isolation

The air-dried powdered leaves (1 kg) of *Phyllanthus emblica* L. were extracted with EtOH/H_2_O (8/2, *v*/*v*, 4 L × 4) by maceration. The solvent was evaporated under reduced pressure at 40 °C to yield 120 g extract. The total EtOH extract was suspended in distilled H_2_O (0.5 L) and partitioned in solvents of increasing polarities, including petroleum ether (1 L × 4), DCM (1 L × 4), EtOAc (1 L × 4) and *n*-BuOH saturated with H_2_O (1 L × 4). The obtained fractions were distilled off in a rotary evaporator under reduced pressure and afforded the corresponding dry residues (12, 0.75, 32, and 62 g, respectively). The ethyl acetate fraction (10 g) was subjected to Sephadex LH-20 (GE Healthcare Biosciences, Uppsala, Sweden) (30 g, 1 × 30 cm) chromatography and eluted with a gradient of H_2_O/MeOH. Eluates were collected as 25 mL/fraction, and similar fractions were pooled together. The H_2_O eluates yielded gallic acid (**1**, 300 mg) upon crystallization from MeOH. The eluate with H_2_O/MeOH (8:2, *v*/*v*) was subjected to RP silica gel column (30 × 1.5 cm, i.d.) eluted with H_2_O/MeOH gradient containing 0.1% formic acid in 5% increments. The H_2_O/MeOH (60/40, *v*/*v*) eluate (100 mg) was subjected to Sephadex LH-20 (30 g, 1 × 30 cm) using H_2_O/MeOH (20/80, *v*/*v*) as a mobile phase to obtain naringenin 7-*O*-(6″-*O*-galloyl)-*β*-d-glucopyranoside (**2**, 8 mg).

A part (10 g) of the *n*-BuOH fraction of the fruit extract, obtained by similar procedures of the leaves, was dissolved in 100 mL distilled water, then centrifuged. The water-soluble materials were then dried under reduced pressure at 40 °C. A total of 1.2 g of the water-soluble dry residue (6 g) was submitted to an MCI gel CHP-20P column (1.1 i.d. × 37 cm) and eluted with H_2_O, H_2_O/MeOH (1/9, 1.5/8.5, 2/8, 3/7, 4/6, and 1/1), and MeOH. Eluates were collected in test tubes by a fraction collector adjusted at 700 drops/fraction. Similar fractions, as monitored by RP-HPLC, were grouped together. The H_2_O eluate afforded a MeOH-insoluble residue, which was purified by repeated washing with MeOH and yielded 3,3′-di-*O*-methyl ellagic acid-4′-*O*-*β*-d-glucopyranoside (**3**, 3 mg). The early eluate with 10% aq. MeOH (36 mg) was subjected to preparative RP-HPLC purification with [H_2_O/CH_3_CN (9.3/0.7, *v*/*v*) + 1% CH_3_COOH] furnished pure 1-*O*-galloylglycerol (**4**, 4.7 mg). The early eluate with 20% aq. MeOH afforded pure 1,6-digalloyl-*β*-d-glucose (**5**, 4.8 mg), the next eluate with H_2_O/MeOH (8:2, *v*/*v*) afforded the tannin-related phenolic flavogallonic acid bislactone [**6**, 3.2 mg], whereas the late eluate (13 mg) afforded corilagin (**7**, 5.4 mg) upon preparative HPLC purification with [H_2_O/CH_3_CN (8.5/1.5, *v*/*v*) + 1% CH_3_COOH]. The fractions eluted with H_2_O/MeOH (7:3, *v*/*v*) were also distinguished as early eluted fractions (16 mg) and middle eluted fractions (11 mg), and late eluted fractions (5 mg). Preparative HPLC purification with [H_2_O/CH_3_CN (8/2, *v*/*v*) + 1% CH_3_COOH] of the early and middle eluted fractions separately furnished pure ethyl gallate (8, 5.4 mg) and [3,4,8,9,10-pentahydroxydibenzo [b,d] pyran-6-one (urolithin M5; **9**, 1.3 mg) from the respective fractions. Preparative HPLC purification with [H_2_O/CH_3_CN (7.5/2.5, *v*/*v*) + 1% CH_3_COOH] of the late eluate with the H_2_O/MeOH (6:4, *v*/*v*) furnished the pure (E)-*p*-coumaroyl-1-*O*-*β*-d-glucopyranoside (**10**, 1.6 mg). The H_2_O/MeOH (4/6, *v*/*v*) eluate was rechromatographed over Sephadex LH-20 (10 × 0.5 cm, i.d.) with MeOH to afford 1,2,4,6-tetra-*O*-galloyl-*β*-d-glucopyranoside (**11**, 30 mg).

### 2.4. Spectroscopic Data of the Isolated Compounds

Gallic acid (**1**)**:** white powder; its ^1^H NMR (DMSO-*d*_6_, 400 MHz) and ^13^C NMR data (DMSO-*d*_6_, 100 MHz) are listed in Table 1.

Prunin 6″-*O*-gallate; Naringenin 7-*O*-(6″-*O*-galloyl)-*β*-d-glucopyranoside (**2**): yellow amorphous powder; its ^1^H NMR (CD_3_OD, 400 MHz) and ^13^C NMR data (CD_3_OD, 100 MHz) are listed in Table 2.

3,3′-di-*O*-methyl ellagic acid-4′-*O*-*β*-d-glucopyranoside (**3**): off-white amorphous powder; its ^1^H NMR DMSO-*d*_6_/D_2_O, 9/1, *v*/*v*, 600 MHz] and ^13^C NMR [DMSO-*d*_6_/D_2_O, 9/1, *v*/*v*, 151 MHz] data are listed in Table 3; its HSQC and HMBC spectra are composed in the Supplementary Materials; ESIMS m/z 491 [M − H]^−^ [19].

1-*O*-galloylglycerol (**4**): colorless prisms (H_2_O); ^1^H NMR [acetone-*d*_6_/D_2_O, 9/1, *v*/*v*, 600 MHz] and ^13^C NMR [acetone-*d*_6_/D_2_O, 9/1, *v*/*v*, 150 MHz] are listed in Table 4; its ^1^H-^1^H COSY, HSQC, and HMBC spectra are composed in the Supplementary Materials [20].

1,6-digalloyl-*β*-D-glucose (**5**): off-white amorphous powder; ^1^H NMR [CD_3_OD/D_2_O, 9/1, *v*/*v*), 600 MHz] are listed in Table 5; its ^1^H-^1^H COSY spectrum is composed in the Supplementary Materials.

Flavogallonic acid bislactone (**6**): off-white amorphous powder; ^1^H NMR [acetone-*d*_6_/D_2_O, 9/1, *v*/*v*, 600 MHz] and ^13^C NMR [acetone-*d*_6_/D_2_O, 9/1, *v*/*v*, 151 MHz] are listed in Table 6; its HSQC and HMBC spectra are composed in the Supplementary Materials.

Corilagin (**7**): yellow amorphous powder; ^1^H NMR [CD_3_OD + D_2_O, 9/1, *v*/*v*, 600 MHz] and ^13^C NMR [CD_3_OD/D_2_O, 9/1, *v*/*v*, 151 MHz] are listed in Table 7; its ^1^H-^1^H COSY, HSQC, and HMBC spectra are composed in the Supplementary Materials [23,24].

Ethyl gallate (**8**): off-white crystals (MeOH); ^1^H NMR [acetone-*d*_6_/D_2_O, 9/1, *v*/*v*, 600 MHz] and ^13^C NMR (acetone-*d*_6_/D_2_O, 9/1, *v*/*v*, 151 MHz) are listed in Table 8; its HSQC and HMBC spectra are composed in the Supplementary Materials [25].

3,4,8,9,10-pentahydroxydibenzo [b,d] pyran-6-one (urolithin M5, **9**): colorless needles; ^1^H NMR [acetone-*d*_6_/D_2_O, 9/1, *v*/*v*, 600 MHz] and ^13^C NMR [acetone-*d*_6_/D_2_O,9/1, *v*/*v*, 151 MHz] are listed in Table 9 [26].

(E)-*p*-coumaroyl-1-*O*-*β*-d-glucopyranoside (**10**): off-white amorphous powder; ^1^H NMR [acetone-*d*_6_/D_2_O, 9/1, *v*/*v*, 600 MHz] and ^13^C NMR [acetone-*d*_6_/D_2_O, 9/1, *v*/*v*, 151 MHz] are listed in Table 10; its ^1^H-^1^H COSY, HSQC, and HMBC spectra are composed in the Supplementary Materials [27].

1,2,4,6-tetra-*O*-galloyl-*β*-d-glucopyranoside (**11**): ^1^H NMR [CD_3_OD, 400 MHz] data are listed in Table 11; its ^13^C NMR spectrum is composed in the Supplementary Materials.

### 2.5. Metabolite Quantification

#### 2.5.1. Total Phenolic Content

The Folin-Ciocalteu method was used to calculate the extract’s total phenolic content [29]. Briefly, an aliquot, 1 mL of each of the gallic acid solutions, and the tested extracts were introduced into a volumetric flask (25 mL) containing distilled water (9 mL); 1 mL of Folin-Ciocalteu reagent was added 5 min later 10 mL of Na_2_CO_3_ (7%) was added, the volume was adjusted with distilled water to the mark. The solution was mixed carefully and left for 90 min, at room temperature. A blank experiment was processed in the same way using 1 mL of distilled water. Absorbance using a UV-VIS spectrophotometer was measured at 750 nm. The standard gallic acid calibration curve (regression equation: y = 0.0334x + 0.0993, R^2^ = 0.9971) was used to obtain the total phenolics. The total phenolic content in each sample was expressed as mg of gallic acid equivalent (GAE)/1 g dry weight of the extract.

#### 2.5.2. Total Flavonoid Content

The aluminium chloride colorimetric technique was used to determine the total flavonoid content of crude extract [30]. One ml of each of the tested samples and standard solutions were separately introduced into 10 mL volumetric flasks containing 4 mL distilled water, followed by the addition of 0.3 mL NaNO_2_ (5%). After 5 min of incubation, 0.3 mL of 10% AlCl_3_ solution was added, and the combination was left to stand for 6 min. After the addition of 2 mL of a 1 M NaOH solution, the final volume of the combination was then raised to 10 mL using double-distilled water. After 15 min of standing time, the mixture was tested for absorbance at 510 nm against a blank solution prepared with 1 mL of distilled water. The standard quercetin calibration curve (regression equation: y = 0.002x + 0.0255, R^2^ = 0.9903) was used to obtain the total flavonoids, which was then reported as mg quercetin equivalent per g dry weight of the extract.

#### 2.5.3. Determination of Tannins Content

Dried powdered plant samples (each 1 g) were thoroughly extracted with 100 mL of distilled water for one h using an ultrasonic bath at room temperature. The extract was filtered in a 100 mL volumetric flask, and the filtrate was adjusted to volume with distilled water. An aliquot (5 mL) of the filtrate was transferred to the test tube and treated with 2 mL of 0.1 M FeCl_3_ solution (dissolved in 0.1 N HCl containing 0.008 M potassium ferrocyanide). The absorbance was measured within 10 min, at 550 nm against a blank solution, prepared with distilled water. The standard calibration curve of gallic acid with ferric chloride (regression equation: y = 0.0042x + 1.1494, R^2^ = 0.9876) was used to obtain the total tannins. The tannin content was calculated in terms of gallic acid equivalent (GAE) mg/1 g dry weight [31].

### 2.6. Nutritional Values

#### 2.6.1. Vitamin C Content

Vitamin C was determined based on Kall and Andersen 1999 [32]. Fresh leaves and fruits (25 g each) were mixed with 100 mL extraction buffer composed of 1% metaphosphoric acid with 0.5% oxalic acid. The mix was transferred into a blender with a further 70 mL of the extraction buffer. Oxygen was evacuated by passing a stream of CO_2_ through the suspension for 3 min. After blending for 5 min, the resulting solutions were quantitatively completed into 250 mL with the extraction buffer. Aliquots were centrifuged for 10 min at 9 × 10^3^ rpm at 4 °C. The supernatant was filtered (0.45-μm filter) before the HPLC analyses according to the conditions shown in (Table 1).

#### 2.6.2. Vitamin E Content

Vitamin E was determined based on the reported method [33]. The ground leaves and fruits (2 g each) were added separately in a 15 mL ethanol containing vitamin C (20%, *v*/*v*) in a saponification vessel and vortex for 30 s. After sonication for 15 min, 5 mL of KOH (33.6 g/L) was added, and the vessel was flushed with Argon gas for 1 min. An air condenser was connected, and the contents were digested at 70 °C for 15 min in a shaking water bath. The samples were cooled for 5 min in an ice bath, and then 20 mL of NaCl (20 g/L) was added and vortexed for 30 s. The unsaponifiable fractions were extracted with diethyl ether (20 mL). The ether was evaporated and the unsaponifiable fractions were extracted three times with 20 mL *n*-heptane containing 0.1 g/L of butylated hydroxyl toluene (BHT). The extracts were collected, then centrifuged and diluted to a final volume of 50 mL and filtered through a 0.2 μm filter prior to HPLC analyses according to the conditions in Table 12. Experiments were repeated three times. The compound peaks were identified based on the retention times of the external standards vitamins C and E. The concentrations were calculated from peak areas determined by linear regression.

#### 2.6.3. Mineral Content

The leaves, stem branches, and fruits (200 mg oven dried of each) were separately washed thoroughly with tap water followed by distilled water, dried at 105 °C, ground using a mortar, and stored in plastic bags until analysis. The dried powdered leaves, stem branches, and fruits (200 mg of each) were added to a polytetrafluoroethylene (PTFE) digestion vessel, 5 mL conc. HNO_3_ and 3 mL conc. H_2_O_2_ were added to the vessel and left for approximately 20 min before the vessel was closed. Sample decomposition was carried out in a microwave digestion system. A top-step program (Table 13) was applied to the samples. The undissolved parts were separated with centrifugation at 4000 rpm for 10 min. The extracts were transferred into a volumetric flask and made up to 25 mL with double distilled water. Blank experiments (n = 3) were carried out. Through analysis of the certified reference material (GBW07605 tea sample), the method’s accuracy was examined [34].

### 2.7. Biological Investigations

#### 2.7.1. Antioxidant Assay

Stock solutions (1 mg/mL) prepared in absolute ethanol of the leaves, stem branches and fruit extracts as well as gallic acid was diluted to final concentrations of 250, 125, 50, 25, and 5 μg/mL in ethanol. DPPH (1,1-Diphenyl-2-picryl-hydrazyl) was used as a free radical scavenger in the antioxidant activity. Ascorbic acid (E-Merck, Germany) was used as a reference drug, while absolute ethanol was used as a control. The ability of the extracts to scavenge DPPH free radicals was assessed using a published method [35]. One ml of 0.3 mM DPPH ethanol solution was added to 2.5 mL of sample solutions of different concentrations and allowed to react at room temperature. After 30 min, the absorbance values were measured at 518 nm and converted into percentage inhibition according to the following formula:

Inhibition (%) = ([(*A*_control_ − *A*_sample_)])/(*A*_control_)] × 100

#### 2.7.2. Antimicrobial Assay

The total ethanolic extract as well as the petroleum ether, methylene chloride, ethyl acetate, and butanol fractions of both leaf and fruit were investigated for their antimicrobial effect against certain Gram-positive bacteria, *Bacillus subtilis* (ATCC-6051), *Staphylococcus aureus* (ATCC-12600), and *Streptococcus faecalis* (ATCC-19433), Gram-negative bacteria, *Escherichia coli* (ATCC-11775), *Pseudomonas aeruginosa* (ATCC-10145), and *Neisseria gonorrhoeae* (ATCC-19424), and fungi, *Candida albicans* (ATCC-26555) and *Aspergillus flavus* (ATCC 15517). The micro-organisms used were obtained from Micro Analytical Center, Cairo University. The agar diffusion method was applied using a Trypticase soy agar (Difco) medium inoculated with the bacterial or fungal suspension of the test organisms [36]. The tested extracts of the plant were dissolved in dimethyl sulfoxide (DMSO) in the concentration of 200 mg/mL, and 50 µL was aseptically transferred into sterile discs (10 mg/disc) of Whatman filter paper (5 mm in diameter). The plates were incubated at 37 °C for 24 h in the case of bacteria and at 25 °C for 48 h in the case of fungi. After incubation, the inhibition zones were recorded in m. Three replicates were carried out and the average inhibition zones were determined.

### 2.8. Investigations of Anti-Acetylcholinesterase by Molecular Dockng

Molecular docking calculations of the target compounds were performed using the AutoDock Vina program [37]. The crystal structure of the protein was retrieved from the Protein Data Bank (PDB ID: 4EY7). The water molecules in the proteins were removed and polar hydrogen was added to make these receptors prepared for docking [38,39,40]. In the 40 Å × 40 Å × 40 Å grid sizes, active protein regions were established for the process. The structure of the selected proteins was parameterized using AutoDock Tools [41]. Furthermore, the molecular docking protocol was validated as reported in the literature [42,43,44]. Compounds used in this study were drawn using Avogadro software (v1.2.0). Affinity scores (in kcal/mol) provided by AutoDock Vina for all compounds were obtained and ranked based on the free energy binding theory (greater negative value means greater binding affinity) [45]. The resulting structures and the binding docking poses were graphically inspected to check the interactions using DS Visualizer 2.5 (http://3dsbiovia.com/products/, accessed on 19 July 2023).

## 3. Results

### 3.1. Identification of Isolated Compounds

**Figure 1:**
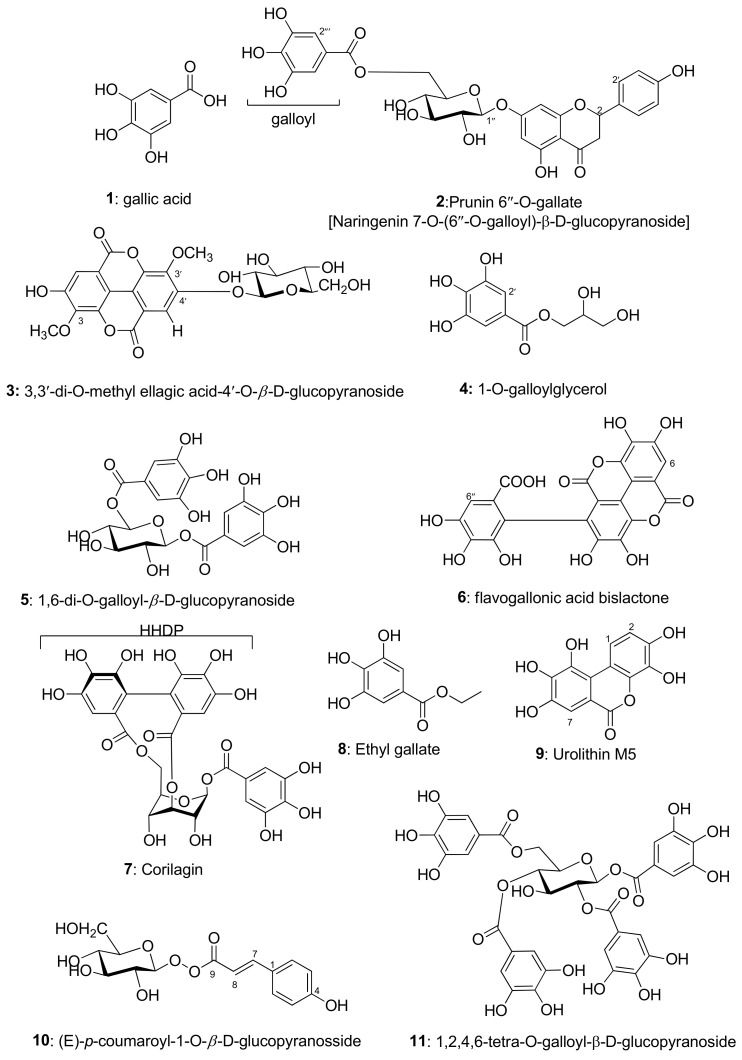
Structures of isolated compounds **1**–**11** from *P. emblica*.

Even though isolating compounds from a natural source is a difficult and time-consuming operation, it is essential to comprehend the phytochemical makeup of the sample and precisely determine the biological effects of the compounds. In this research, we purified eleven phytomolecules from aqueous methanol extracts of leaves and fruits of *P. emblica*. The structures of the isolated compounds (**1**–**11**, Figure 1) were identified from the spectroscopic data [see the listed data in the experimental section (Table 1, Table 2, Table 3, Table 4, Table 5, Table 6, Table 7, Table 8, Table 9, Table 10 and Table 11) and the 1D and 2D NMR spectra composed in the Supplementary Materials file associated with this article] and the comparison with those obtained from the literature. The isolated compounds are categorized as flavonoids, naringenin 7-*O*-(6″-*O*-galloyl)-*β*-d-glucopyranoside (**2**), simple tannin-like phenolics, gallic acid (**1**), 3,3′-di-*O*-methyl ellagic acid-4′-*O*-*β*-d-glucopyranoside (**3**), 1-*O*-galloyl glycerol (**4**), flavogallonic acid bislactone (**6**), ethyl gallate (**8**), urolithin M5 (**9**), (E)-*p*-coumaroyl-1-*O*-*β*-d-glucopyranoside (**10**), and tannins, 1,6-di-*O*-galloyl-*β*-d-glucopyranoside (**5**), corilagin (**7**), and 1,2,4,6-tetra-*O*-galloyl-*β*-d-glucopyranoside (**11**) [17,18,19,20,21,22,23,24,25,26,27]. According to our knowledge, among these isolates, compounds **3** and **10** are first isolated from the plant, and the ^13^C NMR data of compound **2** are reported for the first time in this research.

### 3.2. Total Phenolic, Flavonoid, and Tannin Contents

Results obtained from this quantitative study are recorded in (Table 14). It is obvious that the total phenolic contents were higher in fruits (29 mg GAE/g dry extract) and leaves (29 mg GAE/g dry extract) than stems (mg GAE/g dry weight). The fruits contain the highest quantity of flavonoids (24 mg QE/g dry extract) followed by leaves (13 mg QE/g dry extract) and stem branches (4.5 mg QE/g dry extract). The highest tannins content was present in the stem branches (4.2 mg/g dry weight) followed by leaves (2.48 mg/g dry weight) and fruits (2.2 mg/g dry weight).

### 3.3. The Nutritional Values

Nutritive value of the plant was determined via analysis of vitamins C and E and minerals contents. Analysis results (Table 15) revealed that the fruits contain a high content of vitamin C (282 mg/100 g of fresh fruits). Leaves contain considerable amounts of vitamin C and vitamin E (19, 10 mg/100 g fresh wt.) which make it a rich source for these vitamins. This encourages the use of fresh leaf extracts as a rich vitamin supplement.

Natural minerals content (Table 16) revealed the highest content of potassium is present in fruits (13,510 mg/kg dry weight) which is higher than banana content (3800 mg/kg) [46]. The highest content of sodium is in stem branches (807 mg/kg dry wt.). Leaves contain a high amount of iron (1039 mg/kg), which makes them a good iron supplement. Previous reports on *P. emblica* showed that sodium, potassium, and zinc contents are 4.2, 282, 1.8 mg/100 g fresh weight of fruits [2]. No previous reports dealt with the mineral content of leaves or stem branches.

**Table 15:** Vitamins C and E contents of the leaves and fruits of *P. emblica* L.

	Content (mg/100 g Fresh Sample)		
Vitamin	Leaves	Fruits	RAD for Adults (Amount/Day)
Vitamin C	19 ± 1	282 ± 6	60 mg
Vitamin E	10 ± 0.9	0.34 ± 0.001	13 mg

RAD: recommended dietary allowances [47].

### 3.4. Biological Properties

#### 3.4.1. The Antioxidant Activity

**Figure 2:**
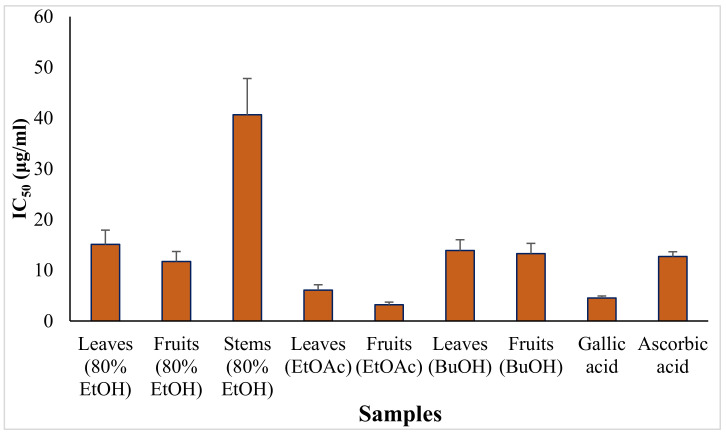
IC_50_ of DPPH• scavenging activity of different *P. emblica* extracts in comparison with isolated gallic acid and standard ascorbic acid. Error bars indicate standard errors of the means of triplicate experiments.

The antioxidant activity of the various extracts of *P. emblica* was tested by the DPPH• free radical scavenging method. The IC_50_ values were compared with that of ascorbic acid. The results showed that the H_2_O/ethanol (2/8, *v*/*v*) extract of the fruits exhibited antioxidant activity that was higher by 8% than the standard ascorbic acid, while the ethyl acetate fraction of the fruits was the most active fraction followed by that of the leaves with IC_50_ values of 3.20 and 6.10 μg/mL, respectively (Figure 2). The ethyl acetate obvious antioxidant activity may be attributed to the accumulation of a large amount of the powerful antioxidant gallic acid [(**1**), IC_50_ 4.53)] and the flavonoid (**2**) (see extraction and isolation section). Previous numerous in vitro studies, including those using the DPPH radical, the 2,2′-azino-bis (3-ethylbenzothiazoline-6-sulfonic acid) (ABTS), nitric oxide radical, the ferric reducing antioxidant power (FRAP), and the LDL oxidation assay methods [8], support our antioxidant activity of *P. emblica* which is herein shown.

#### 3.4.2. Antimicrobial Activity

The antimicrobial screening results (Table 17) revealed that leaf extracts showed the most powerful and broad-spectrum antimicrobial activity against all tested bacterial species. The total extract of leaves exhibited a wide inhibition zone against *B. subtilis*, *S. aureus*, and *P. aeruginosa*, which is ~75% of the inhibition zone of the tetracycline antibiotic. The EtOAc and BuOH subextracts also exhibited a comparable inhibition zone against the same bacterial species. None of the petroleum ether and the MeOH subextracts exhibited strong activity against the bacterial species. Fruit extracts showed moderate broad-spectrum activity against tested bacteria. Total ethanolic extracts of leaves and fruits, and ethyl acetate subextracts of leaves and butanol subextract of fruit, exhibit moderate activity against Candida albicans. None of the extracts exhibited activity against *Aspergillus flavus*.

### 3.5. Computatioal Investigation Antiacetylcholinesterase Properties of Isolated Compounds

In a recent study, a crude methyl extract of *P. emblica* fruit has been found strongly active in inhibiting acetylcholinesterase (AChE) and butyrylcholinesterase with IC_50_ of 53.88 µg/mL and 65.12 µg/mL, respectively [48]. This suggests that *P. emblica* is a rich source of AChE and BuChE inhibitors. It would be a significant achievement if any compound obtained from *P. emblica* has the potential to be an effective treatment for Alzheimer’s disease. In the current study, the molecular docking evaluation of the anticholinesterase action of the isolated molecules was attempted. The results of the molecular docking investigation (Table 18) revealed a high binding affinity (low docking score) and hence a possible acetylcholinesterase inhibitory effect of the compounds. The highest binding affinity was seen among the ligand prunin 6″-*O*-gallate (naringenin 7-*O*-(6″-*O*-galloyl)-*β*-d-glucopyranoside (**2**, −12.4 kcal/mol)]. The tannins 1,2,4,6-tetra-*O*-galloyl-***β***-d-glucopyranoside (**11**) and 1,6-di-*O*-galloyl-*β*-d-glucopyranoside (**6**) and corilagin (**7**) were the next in their binding affinities with docking scores of −11.1, −10.7, and −10.1 kcal/mol, respectively. Next, urolithin M5 (**9**) also exhibited a considerably high binding affinity (−9.8 kcal/mol].

**Figure 3:**
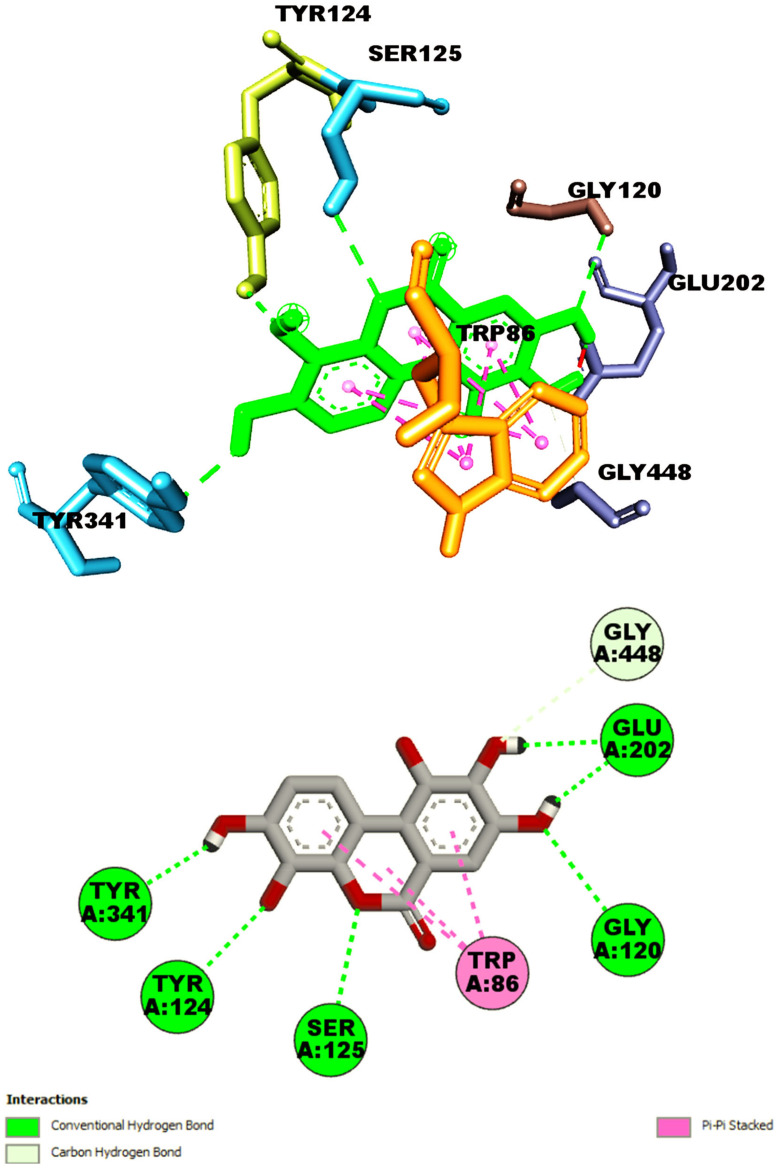
Shown interactions of urolithin M5 (**9**) in the active site of AChE (PBD ID: 4EY7).

**Figure 4:**
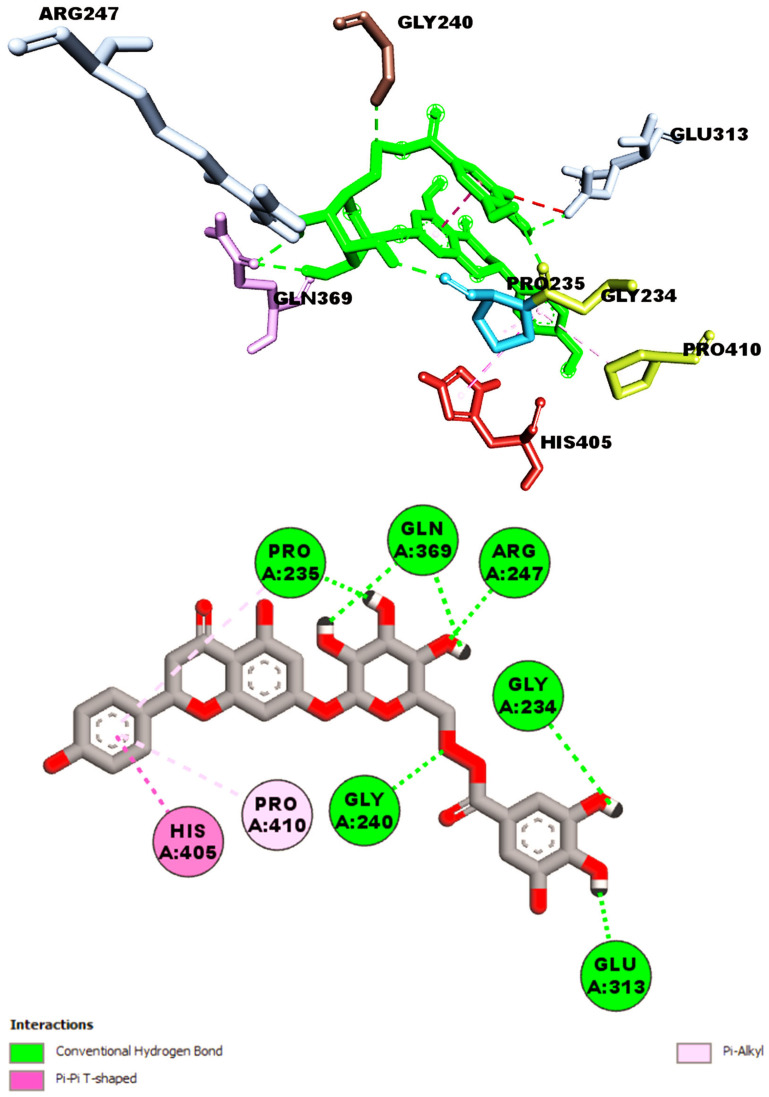
Shown interactions of naringenin 7-*O*-(6″-*O*-galloyl)-*β*-d-glucopyranoside (**2**) in the active site of AChE (PBD ID: 4EY7).

The molecular docking studies of AChE to urolithin M5 (**9**) demonstrated that the molecule could establish H-bond with six various amino acids of the target receptor, including GLY120, TYR124, SER125, GLU202, TYR341, and GLY448. Moreover, one π-π stacked interaction was also found with TRP86 (Figure 3). On the other hand, as shown in Figure 4, the docking pose accomplished by naringenin 7-*O*-(6″-*O*-galloyl)-*β*-d-glucopyranoside involved six interactions with amino acid residues of GLY234, PRO235, GLY240, ARG247, GLU313, and GLN369 via hydrogen bond interaction. Additionally, three hydrophobic interactions were also observed with PRO235 and PRO410 (π-alkyl) and HIS405 (π-π T shaped), thus resulting in the binding affinity of −12.4 kcal/mol.

## 4. Discussion

Numerous research publications have recently concentrated on the qualities of various foods (functional food or nutraceuticals) and established that functional foods and their ingredients have a part in lowering the risk of disease, treating it, and promoting human health. Consuming such nutraceuticals in moderation helps keep the redox state steady and reduce oxidative stress.

In our study, the leaves’ and fruits’ ethyl acetate and butanol fractions exhibited DPPH radical scavenging activity comparable to the known antioxidant gallic and ascorbic acids, respectively (Figure 2). This is consistent with the high levels of total phenolics, total flavonoids, and total tannins (Table 3) and overemphasized by the isolation of eleven compounds belonging to tannins [1,6-di-*O*-galloyl-*β*-d-glucopyranoside (**5**), corilagin (**7**), and 1,2,4,6-tetra-*O*-galloyl-*β*-d-glucopyranoside (**11**)], tannin-related phenolics [3,3′-di-*O*-methyl ellagic acid-4′-*O*-*β*-d-glucopyranoside (**3**), 1-*O*-galloyl glycerol (**4**), and flavogallonic acid bislactone (**6**), and urolithin M5 (**9**)], simple phenolics [gallic acid (**1**), ethyl gallate (**8**), and (E)-p-coumaroyl-1-*O*-*β*-d-glucopyranoside (**10**)], and a flavonoid [naringenin 7-*O*-(6″-*O*-galloyl)-*β*-d-glucopyranoside (**2**)].

According to previous research, one of the putative preventive benefits connected to the Amla bioactive compounds is the mitigation of neurological modifications, particularly the biochemical changes seen in carriers of Alzheimer’s disease. A crude methyl extract of *P. emblica* fruit was found to be strongly active in inhibiting acetylcholinesterase (AChE) and butyrylcholinesterase (BuChE) [48]. In another in vitro study, *P. emblica* fruit polyphenols exhibited strong antioxidant capacities in scavenging free radicals and anti-cholinesterase ability by inhibition of AChE and BuChE. This suggests that *P. emblica* is a rich source of phytochemicals with antioxidants and inhibitors of AChE and BuChE.

To discover further about the protective effects of Amla bioactive molecules against Alzheimer’s disease, the acetylcholinesterase inhibitory effect of isolated compounds **1**–**11** was computationally investigated. Results of molecular docking (Table 18) revealed a high binding affinity (low docking score) and hence a possible high acetylcholinesterase inhibitory effect of the compounds. The highest binding affinity was seen among the ligand and prunin 6″-*O*-gallate (**2**, −12.4 kcal/mol), the tannins 1,2,4,6-tetra-*O*-galloyl-***β***-d-glucopyranoside (**11**, −11.1 kcal/mol), 1,6-di-*O*-galloyl-*β*-d-glucopyranoside (**6**, −10.7), corilagin (**7**, −10.1 kcal/mol), and urolithin M5 (**9**, −9.8 kcal/mol).

The acetylcholinesterase inhibitory effects of naringenin 7-*O*-(6″-*O*-galloyl)-*β*-d-glucopyranoside were not reported early; however, the aglycone part, naringenin, showed a diminishing effect on amnesia due to its inhibitory effect on acetylcholinesterase [49]. It also attenuated behavioral instabilities induced by social–conquer tension in mice via the inhibition of acetylcholinesterase activity, oxidative stress, and release of pro-inflammatory cytokines [50]. In addition, other suggested anti-AD of naringenin were seen in a series of studies [51,52,53,54,55].

On the other hand, ellagitannin-rich food extracts were found essential for both preventing and treating neurological disorders including Alzheimer’s disease (AD). Urolithins, the intestinal microbial metabolites produced from ellagitannin- and ellagic-acid-containing foods, with more bioavailability than their precursors, contribute significantly to the beneficial properties attributed to these polyphenolic compounds. The potential mechanisms of urolithins have been investigated based on in vitro and in vivo tests in several studies. These studies support the positive effects of urolithins in the treatment of several diseases, including Alzheimer’s disease, type 2 diabetes mellitus, liver disease, cardiovascular disease, and various cancers [56,57].

In previous research, *P. emblica* fruit methanol extract was found to be strongly active in inhibiting AChE (IC_50_ 53.88 µg/mL) and BuChE (IC_50_ 65.12 µg/mL) [48]. The *P. emblica* fruit polyphenols exhibited strong inhibition of AChE (IC_50_ 0.2186 ± 0.0416 mg/mL) and BuChE (IC_50_ 0.0542 ± 0.0054 mg/mL), and strong antioxidant capacities in scavenging free radicals [58]. Individual molecules, myricetin, quercetin, fisetin, and gallic acid, isolated from *P. emblica*, exerted low docking scores and strong inhibition ability on AChE with IC_50_ values of 0.1974 ± 0.0047, 0.2589 ± 0.0131, 1.0905 ± 0.0598, and 1.503 ± 0.0728 mM, respectively [59]. In animal models, the fruit extracts have demonstrated protective benefits on organs/tissues against damages brought on by chemicals, stressors, and aging [60]. These outcomes support our findings and highlight the potential of *P. emblica* as a source of bioactive components.

Furthermore, investigation of Amla as a source of nutraceuticals revealed high vitamin C (ascorbic acid) and E (α-tocopherol) (Table 4) and mineral (Zn, Fe, K, and Na) content (Table 5). The vitamin C content in 100 g of fruit is 4.7 times the recommended dietary allowance, while vitamin E content in 100 g of leaves is 0.78% the recommended dietary allowance of vitamins [47]. Vitamin C and E are both antioxidants food ingredients that are established to have a neuroprotective impact by either lowering or avoiding oxidative damage [61]. Vitamin C is a necessary food for humans and serves as a redox buffering that can lower reactive oxygen species and so neutralize them. It is a crucial cofactor and electron donor for enzymes involved in a wide range of biological processes, including stimulating the immune system, producing collagen, hormones, and neurotransmitters, regulating cell division and growth, and removing heavy metals from the body [62]. A lipid soluble vitamin E is assumed to have neuroprotective effects by either lowering or avoiding oxidative damage through preventing the spread of lipid radicals and stops lipid peroxidation chain reactions in cellular membranes [63]. In summary, even though Alzheimer’s disease cannot be reversed or cured, exploring *P. emblica* as a functional food rich in acetylcholinesterase inhibitors would be a beneficial treatment option for problems with memory, cognition, and other mental functions.

The numerous polyphenolics (**1**–**11**) in addition to the high content of vitamins C and E with potent antioxidants point to the possibility that antioxidative activity is one of the *P. emblica* biological mechanisms underpinning Alzheimer’s disease, which makes *P. emblica* a useful functional food with neuroprotective properties.

Results of antimicrobial screening of Amla leaves and fruit extracts (Table 17) revealed superior antimicrobial activity of leaves over the fruits’ tested extract. The active molecules were accumulated mainly in the EtOAc and BuOH subextracts. The most sensitive bacterial species (exhibiting a wide inhibition zone) were *B. subtilis*, *S. aureus*, and *P. aeruginosa*, whereas the most sensitive fungal species was Candida albicans. Among the isolated compounds, corilagin (**7**) inhibited the growth of *E. coli*, *S. aureus*, and *C. albicans*. It exhibited membrane permeability disruption of *E. coli* and *C. albicans*, but acted on extracellular fibrinogen-binding protein, the response regulator SaeR protein, and sensor kinase SaeS of *S. aureus* [64]. Further, the reported antimicrobial activity of several isolated compounds, including gallic acid (**1**) [65], flavogallonic acid bislactone (**6**) [22,66], ethyl gallate (**9**) [67,68], and urolithin M5 (**9**) [69], against a broad spectrum of bacterial and viral species also emphasizes our antimicrobial findings (Table 17).

## 5. Conclusions

Oxidative stress is a key player in the pathophysiology of diseases such as atherosclerosis, cancer, Parkinson’s, and Alzheimer’s. Lower overall serum antioxidant components decrease the antioxidant capacity of the body. Functional foods or nutraceuticals have the capacity to function as natural antioxidants. Consuming such nutraceuticals in moderation helps keep the redox state steady and thus reduce oxidative stress damage. Our herein findings highlight P. emblica as an affordable source rich in natural vitamins, minerals, and polyphenolic compounds. Our findings from biological studies suggest that these components may integrate together to mitigate oxidative damage, treat microbial infections, and alleviate Alzheimer’s symptoms by acetylcholinesterase inhibition.

## Figures and Tables

**Table 1:** NMR data of compound **1** in comparison with the literature data [17].

	Compound 1		Gallic Acid (in DMSO-*d*_6_) [17]	
Position	*δ*_H_ ppm	*δ*_C_ ppm	*δ*_H_ ppm	*δ*_C_ ppm
1	–	121.2	–	120.9
2	6.91, s	109.2	6.91, s	109.2
3	–	145.8	–	145.9
4	–	138.4	–	138.4
5	–	145.8	–	145.8
6	6.91, s	109.2	6.91, s	109.2
7	–	168.2	–	167.9

**Table 2:** NMR data of compound **2** in comparison with the literature data [18].

	Compound 2		Prunin 6″-*O*-Gallate in (CD_3_)_2_CO [18]
Position	*δ*_H_ ppm (H, mult., *J* in Hz)	*δ*_C_^a^ ppm	*δ*_H_ ppm (H, mult., *J* in Hz)
2	5.38 (1H, dd, *J* = 12.6, 3.2)	78.8	5.41 (1H, td, *J* = 12)
3	2.77 (H-3 *eq*. dd, *J* = 17.1, 3.2) 3.16 (H-3 *ax*. dd, *J* = 17.1, 12.6)	42.4	3.0 (2H, m)
4	–	198.0	–
5	–	163.1	–
6	6.18 (H, d, *J* = 2.5)	96.7	6.23 (H, br s)
7	–	165.1	–
8	6.18 (H, d, *J* = 2.5)	95.4	6.23 (H, br s)
9	–	163.1	–
10	–	103.4	–
1′	–	129.4	–
2′, 6′	7.28 (2H, d, *J* = 8.5)	127.9	7.35 (2H, d, *J* = 9)
3′, 5′	6.81 (2H, d, *J* = 8.5)	115.1	6.87 (2H, d, *J* = 9)
4′	–	157.2	–
1″	5.04 (1H, d, *J* = 7.2)	99.3	5.18 (1H, d, *J* = 7)
2″	3.79 (1H, m)	73.1	3.3–4.1 (4H, m)
3″	3.50 (3H, m)	76.1	
4″		70.5	
5″		74.1	
6″	4.61 (1H, dd, *J* = 12.0, 2.2) 4.41 (1H, dd, *J* = 12.0, 5.9)	63.4	4.50 (2H, m)
1″′	–	119.7	–
2‴, 6‴	7.06 (2H, s)	109.1	7.07 (2H, s)
3‴, 5‴	–	145.0	–
4‴	–	138.6	–
7‴	–	167.2	–

^a^ The ^13^C NMR data of compound **2** are first-time reported in this research.

**Table 3:** NMR data of compound **3**.

Position	*δ*_H_ ppm (H, mult., *J* in Hz)	*δ*_C_ ppm
1	–	111.1
2	–	140.9
3	–	140.1
4	–	151.5
5	7.54 (1H, s)	111.6
6	–	111.9
7	–	158.3
1′	–	114.1
2′	–	141.6
3′	–	141.8
4′	–	152.8
5′	7.81 (1H, s)	111.9
6′	–	112.7
7′	–	158.3
3-*O*-Me	4.05	61.0
3′-*O*-Me	4.09	61.6
1″	5.14 (1H, d, *J* = 7.8 Hz, H-1″)	101.3
2″	3.35 (2H, overlapped with solvent signal)	73.3
3″		76.4
4″	3.27 (1H, t, *J* = 9)	69.5
5″	3.43 (1H, ddd, *J* = 2.4, 5.4, 12)	77.2
6″	3. 70 (1H, d, *J* = 12.6),	60.5
	3.52 (1H, dd, *J* = 5.4, 12.6)	

**Table 4:** NMR data of compound **4**.

Position	*δ*_H_ ppm (H, mult., *J* in Hz)	*δ*_C_ ppm
1	4.24 (1H, dd, *J* = 11.4, 4.3)	66.2
	4.15 (1H, d, *J* = 11.4)	
2	3.92 (1H, m, H-2)	70.3
3	3.58 (2H, m, H-3)	63.2
1′	–	120.8
2′	–	109.7
3′	–	145.6
4′	–	138.8
5′	7.81 (1H, s)	145.6
6′	–	109.7
7′	–	167.5

**Table 5:** ^1^H NMR data of compound **5** in comparison with the literature data [21].

Position	Compound 5	1,6-Digalloyl-*β*-d-glucose (CD_3_OD) [21]
	*δ*_H_ ppm (H, mult., *J* in Hz)	*δ*_H_ ppm (H, mult., *J* in Hz)
Glucose 1	5.67 (1H, d, *J* = 7.8)	5.73 (1H, d, *J* = 7.8)
2	3.55–3.75 (4H, m)	3.55–3.75 (4H, m)
3		
4		
5		
6	4.39 (1H, dd, *J* = 12.0, 5.4)	4.44 (1H, dd, *J* = 12.0, 5.4)
	4.55 (1H, dd, *J* = 12.0, 2.0)	4.59 (1H, dd, *J* = 12.0, 1.8)
Galloyl [(H-2/H-6) × 2]	7.08, 7.13 (each 2H, s)	7.12, 7.17 (each 2H, s)

**Table 6:** NMR data of compound **6** in comparison with the literature data [22].

	Compound 6		Flavogallonic Acid Bislactone in CD_3_OD [22]	
Position	*δ*_H_ ppm (H, mult., *J* in Hz)	*δ*_C_ (ppm)	*δ*_H_ ppm (H, mult., *J* in Hz)	*δ*_C_ ppm
1	–	108.8 ^a^	–	108.1
2	–	136.3	–	135.7
3	–	137.3	–	136.3
4	–	137.8	–	136.5
5	–	111.1	–	112.8
6	7.29 (1H, s)	110.9	7.26 (1H, s)	110.1
7		160.5		160.4
1′		109.0 ^a^		108.1
2′	–	138.9	–	137.8
3′	–	139.8	–	139.2
4′	–	144.1	–	143.2
5′	–	118.1	–	117.5
6′	–	113.9	–	114.4
7′	–	158.5	–	158.9
1″	–	125.8	–	124.9
2″	–	121.3	–	120.2
3″	–	144.7	–	144.1
4″	–	146.6	–	145.9
5″	–	148.4	–	147.8
6″	7.57 (1H, s)	113.2	7.50 (1H, s)	113.3
7″	–	168.2	–	168.9

^a^ exchangeable.

**Table 7:** NMR data of compound **7.**

Position	*δ*_H_ ppm (H, mult., *J* in Hz)	*δ*_C_ ppm
Glucose 1	6.34 (1H, d, *J* = 2)	95.84
2	4.00 (1H, brs)	70.07
3	4.81 (1H, brs)	72.31
4	4.45 (1H, brs)	63.22
5	4.52 (1H, br t, *J* = 8)	76.89
6	4.15 (1H, dd, *J* = 11, 8)	65.76
	4.92 (1H, t, *J* = 11)	
Galloyl 1	–	121.3
2/6	7.05 (2H, s)	111.8
3/5	–	147.1
4	–	141.2
7	–	167.6
HHDP 1,1′	–	117.4, 118.0
2,2′	–	126.2, 126.3
3,3′	6.69, 6.66 (each 1H, s)	109.1, 111.1
4,4′	–	146.4, 146.8
5,5′	–	138.7, 139.0
6,6′	–	146.0, 146.1
7,7′	–	169.4, 170.9

**Table 8:** MR data of compound **8**.

Position	*δ*_H_ ppm (H, mult., *J* in Hz)	*δ*_C_ ppm
Galloyl 1	–	121.0
2/6	7.06 (2H, s)	109.5
3/5	–	145.9
4	–	138.7
7	–	167.2
Ethyl CH_2_	4.21 (2H, q, *J* = 7.1)	60.9
Ethyl CH_3_	1.27 (3H, t, *J* = 7.1)	14.5

**Table 9:** NMR data of compound **9**.

	Compound 9		Urolithin M5 in CD_3_OD [26]	
Position	*δ*_H_ ppm (H, mult., *J* in Hz)	*δ*_C_ ppm	*δ*_H_ ppm (H, mult., *J* in Hz)	*δ*_C_ ppm
1	8.4 (1H, d, *J* = 9)	118.4	8.44 (1H, d, *J* = 9)	119.2
2	6.8 (1H, d, *J* = 9)	112.0	6.77 (1H, d, *J* = 9)	112.5
3	–	145.9	–	144.0
4	–	133.5	–	133.3
4a	–	140.3	–	140.9
5	–	–	–	–
6	–	162.1	–	163.9
6a	–	112.0	–	112.0
7	7.40 (1H, s)	107.9	7.37 (1H, s)	108.2
8	–	146.0	–	146.4
9	–	147.8	–	146.7
10	–	143.3	–	141.9
10a	–	112.2	–	112.8

**Table 10:** NMR data of compound **10**.

Position	*δ*_H_ ppm (H, mult., *J* in Hz)	*δ*_C_ ppm
1	–	118.1
2/6	7.42 (2H, dd, *J* = 8.4, 9.0)	129.7
3/5	7.68 (2H, dd, *J* = 8.4, 9.0)	129.1
4	–	146.8
7	7.76 (1H, d, *J* = 16)	135.0
8	6.56 (1H, d, *J* = 16)	131.4
9	7.40 (1H, s)	166.2
Glucose 1	5.57 (1H, d, *J* = 8.1)	95.4
2	3.39–3.53 (4H, m)	77.3
3		73.4
4		70.6
5		78.2
6	3.80 (1H, dd, *J* = 12.1, 2.4)	62.0
	3.65 (1H, dd, *J* = 12.1, 5.4)	

**Table 11:** ^1^H NMR data of compound **11** in comparison with 2.5.1 the literature data [28].

	Compound 11	1,2,4,6-Tetra-*O*-galloyl-*β*-d-glucopyranoside in CD_3_OD [28]
Position	*δ*_H_ ppm (H, mult., *J* in Hz)	*δ*_H_ ppm (H, mult., *J* in Hz)
Galloyls H-2/H-6	7.12, 7.11, 7.07, 7.05 (each 2H, s)	7.12, 7.11, 7.07, 7.05 (each 2H, s)
Glucose 1	6.07 (1H, d, *J* = 8.4)	6.07 (1H, d, *J* = 8.4)
2	5.36 (1H, dd, *J* = 8.4, 9.6)	5.37 (1H, dd, *J* = 8.4, 9.6)
3	4.18 (1H, t, *J* = 9.6)	4.16 (1H, t, *J* = 9.6)
4	5.39 (1H, t, *J* = 9.6)	5.38 (1H, t, *J* = 9.6)
5	4.22 (1H, m)	4.19 (1H, m)
6	4.48 (1H, dd, *J* = 1.8, 12.3)	4.49 (1H, dd, *J* = 1.8, 12.3)
	4.31 (1H, dd, *J* = 4.2, 12.6)	4.29 (1H, dd, *J* = 4.2, 12.6)

**Table 12:** Conditions of HPLC analysis of vitamin C and E.

Condition	Vitamin C	Vitamin E
Column	RP C-18 Jupiter ODS-2 (5 μm)	RP C-18 Nova Pak ODS-2 (4 μm)
Dimensions	250 × 4.6 nm i.d.	300 × 3.9 mm i.d.
Mobile phase	2.3 mM Na_2_EDTA in 66 mM phosphate-20 mM acetate buffer (pH = 4.50)	Isocratic (isopropanol: heptane, 1: 99, *v*/*v*)
Flow rate	1.2 mL/min	2 mL/min
Detector	UV spectrophotometer	Photodiode array detector (PDA)
Detector UV wavelength	247 nm	195–330 nm
Injection volume	20 μL	10 μL
Column temperature	20 °C	40 °C
Run time	14 min	13 min
Photodiode array (PDA) measurement frequency	-----	1 spectrum/s
PDA spectral resolution	-----	1.2 nm

**Table 13:** Operating conditions of inductively coupled plasma mass spectrometry (ICP-MS).

Nebulizer	Babington Type
Spray chamber	Quartz, double pass
Radiofrequency (RF) generator Frequency	10 MHz, power output:1220W
Air flow rate (L/min)	20
Auxiliary gas flow rate (L/min)	0.9
Nebulizer gas flow rate (L/min)	1–1.2
Sample uptake (L/min)	400
Number of replicates	3
Integration time	0.1
Internal standards	Bi, Be, Rh, Sc
Isotopes	^57^Fe, ^66^Zn, ^39^K, ^22^Na
UV wavelengths of the determined minerals.	
Mineral	Wavelength
Sodium	589.592 nm
Potassium	766.490 nm
Zinc	206.200 nm
Iron	238.204 nm

**Table 14:** Total phenolic, flavonoid, and tannin contents in different organs of *Phyllanthus emblica* L.

Plant Organ	Total Phenolics (mg GAE/g Dry Extract)	Total Flavonoids (mg QE/g Dry Extract)	Total Tannins (mg GAE/g Dry Extract)
Leaves	29 ± 1	13 ± 0.2	2.5 ± 0.1
Fruits	29 ± 1	24 ± 0.4	2.2 ± 0.2
Stem branches	8 ± 1	4.5 ± 0.1	4.2 ± 0.1

**Table 16:** Amount of minerals detected in the leaves, stem branches, and fruits of *P. emblica.*

Mineral	Content (mg/kg Dry Weight)			RAD for Adults (Amount/Day)
	Leaves	Stem Branches	Fruits	
Zinc	17 ± 2	20 ± 1	4 ± 0.2	15 mg
Sodium	443 ± 64	807 ± 7	194 ± 4	23 mg
Potassium	10,725 ± 136	8665 ± 11	13,510 ± 11	90 mg
Iron	1039 ± 10	25 ± 4	NA	10 mg

NA: not analysed. RAD: recommended dietary allowances [47].

**Table 17:** Antimicrobial screening of different extracts of *P. emblica*.

Extract		Bacterial Species												Fungal Species			
	Organ	*B.* *subtilis*		*S.* *faecalis*		*S.* *aureus*		*E.* *coli*		*P.* *aeruginosa*		*N.* *gonorrhoeae*		*C.* *albicans*		*A. flavus*	
		I. Z.^a^	%^b^	I. Z.^a^	% ^b^	I. Z.^a^	% ^b^	I. Z.^a^	% ^b^	I. Z.^a^	% ^b^	I. Z.^a^	% ^b^	I. Z.^a^	% ^b^	I. Z.^a^	% ^b^
Tot. EtOH	L	22 ± 2	73	16 ± 1	53	21 ± 1	75	19 ± 1	63	23 ± 2	74	18 ± 1	62	10 ± 1	50	0	0
	F	13 ± 1	43	14 ± 1	47	12 ± 1	43	15 ± 1	50	14 ± 1	45	12 ± 1	41	9 ± 1	45	0	0
Pet. Ether	L	16 ± 2	53	15 ± 1	50	19 ± 2	68	15 ± 1	50	16 ± 1	52	16 ± 1	55	0	0	0	0
	F	13 ± 1	43	13 ± 1	43	13 ± 1	46	13 ± 1	43	13 ± 1	42	12 ± 1	41	0	0	0	0
EtOAc	L	21 ± 1	70	19 ± 1	63	23 ± 1	82	17 ± 1	57	20 ± 2	65	23 ± 1	79	9 ± 0.2	45	0	0
	F	14 ± 1	47	13 ± 1	43	13 ± 1	46	13 ± 1	43	12 ± 1	39	12 ± 1	41	0	0	0	0
BuOH	L	18 ± 11	60	17 ± 1	57	23 ± 1	82	19 ± 1	63	20 ± 1	65	23 ± 1	79	0	0	0	0
	F	11 ± 1	37	12 ± 1	40	15 ± 1	54	11 ± 1	37	16 ± 1	52	13 ± 1	45	10 ± 1	50	0	0
MeOH	L	13 ± 1	43	12 ± 1	40	10 ± 1	36	12 ± 1	40	11 ± 1	35	10 ± 1	34	0	0	0	0
	F	1 ± 0.1	3	11 ± 1	37	12 ± 1	43	13 ± 1	43	11 ± 1	35	11 ± 1	38	0	0	0	0
Tetra.	L	30 ± 2	100	30 ± 1	100	28 ± 1	100	30 ± 1	100	31 ± 1	100	29 ± 2	100	--	--	--	
Ampho.	F	--	--	--	--	--	--	--	--	--	--	--	--	20 ± 2	100	17	100

^a^ I. Z: inhibition zone in mm. ^b^: % of I. Z. of a sample relative to the corresponding antimicrobial standard, calculated from the equation (I.Z. of a sample/I.Z. of the corresponding standard antimicrobial × 100). Each value is the means of three readings ± SE. L.: leaf; F.: fruit; Tot. EtOH, 80 ethanol extract; Pet. ether: petroleum ether fraction; Met.ch.: methylene chloride fraction; Eth.ac.: ethyl acetate fraction; But.: butanol fraction; Tetra.: tetracycline; Ampho.: amphotericin B.

**Table 18:** Molecular docking results.

No	Compound	Binding Affinity (kcal/mol)
1	gallic acid	−6.6
2	Prunin 6″-*O*-gallate (Naringenin 7-*O*-(6″-*O*-galloyl)-*β*-d-glucopyranoside)	−12.4
3	3,3′-di-*O*-methyl ellagic acid-4′-*O*-*β*-d-glucopyranoside	−9.7
4	1-*O*-Galloylglycerol	−7.8
5	1,6-di-*O*-galloyl-*β*-d-glucopyranoside	−10.7
6	flavogallonic acid bislactone	−9.3
7	Corilagin	−10.1
8	Ethyl gallate	−7.1
9	Urolithin M5	−9.8
10	(E)-*p*-coumaroyl-1-*O*-*β*-d-glucopyranoside	−9.2
11	1,2,4,6-tetra-*O*-galloyl-*β*-d-glucopyranoside	−11.1

## Data Availability

Raw NMR data spectra are available with the corresponding author and ready to supply upon request.

## Supplementary Materials

The following supporting information can be downloaded at: https://www.mdpi.com/article/10.3390/metabo13091013/s1, Figure S1. ^1^H NMR spectrum of compound **1** (DMSO-*d*_6_, 400 MHz); Figure S2. ^13^C NMR spectrum of compound **1** (DMSO-*d*_6_, 100 MHz); Figure S3. ^1^H NMR spectrum of compound **2** (CD_3_OD, 400 MHz); Figure S4. DPTQ NMR spectrum of compound **2** (CD_3_OD, 100 MHz); Figure S5. ^1^H NMR spectrum of compound **3** [acetone-*d*_6_/D_2_O, 9/1, *v*/*v*, 600 MHz]; Figure S6. ^13^C NMR spectrum of compound **3** [acetone-*d*_6_/D_2_O, 9/1, *v*/*v*, 151 MHz]; Figure S7. HSQC spectrum of compound **3** [acetone-*d*_6_/D_2_O, 9/1, *v*/*v*, 600 MHz]; Figure S8. HMBC spectrum of compound **3** [acetone-*d*_6_/D_2_O, 9/1, *v*/*v*, 600 MHz]; Figure S9. ^1^H NMR spectrum of compound **4** [acetone-*d*_6_/D_2_O, 9/1, *v*/*v*, 600 MHz]; Figure S10. ^13^C NMR spectrum of compound **4** [acetone-*d*_6_/D_2_O, 9/1, *v*/*v*, 151 MHz]; Figure S11. ^1^H–^1^H COSY spectrum of compound **4** [acetone-*d*_6_/D_2_O, 9/1, *v*/*v*, 600 MHz]; Figure S12. HSQC spectrum of compound **4** [acetone-*d*_6_/D_2_O, 9/1, *v*/*v*, 600 MHz]; Figure S13. HMBC spectrum of compound **4** [acetone-*d*_6_/D_2_O, 9/1, *v*/*v*, 600 MHz]; Figure S14. ^1^H NMR spectrum of compound **5** [acetone-*d*_6_/D2O, 9/1, *v*/*v*, 600 MHz]; Figure S15. 1H–1H COSY spectrum of compound **5** [acetone-*d*_6_/D2O, 9/1, *v*/*v*, 600 MHz]; Figure S16. ^1^H NMR spectrum of compound **6** [acetone-*d*_6_/D2O, 9/1, *v*/*v*, 600 MHz]; Figure S17. ^13^C NMR spectrum of compound **6** [acetone-*d*_6_/D_2_O, 9/1, *v*/*v*, 151 MHz]; Figure S18. HSQC spectrum of compound **6** [acetone-*d*_6_/D_2_O, 9/1, *v*/*v*, 600 MHz]; Figure S19. HMBC spectrum of compound **6** [acetone-*d*_6_/D_2_O, 9/1, *v*/*v*, 600 MHz]; Figure S20. ^1^H NMR spectrum of compound **7** [acetone-*d*_6_/D_2_O, 9/1, *v*/*v*, 600 MHz]; Figure S21. ^1^H–^1^H COSY spectrum of compound **7** [acetone-*d*_6_/D_2_O, 9/1, *v*/*v*, 600 MHz]; Figure S22. ^13^C NMR spectrum of compound **7** [acetone-*d*_6_/D_2_O, 9/1, *v*/*v*, 151 MHz]; Figure S23. HSQC spectrum of compound **7** [acetone-*d*_6_/D_2_O, 9/1, *v*/*v*, 600 MHz]; Figure S24. HMBC spectrum of compound **7** [acetone-*d*_6_/D_2_O, 9/1, *v*/*v*, 600 MHz]; Figure S25. ^1^H NMR spectrum of compound **8** [acetone-*d*_6_/D_2_O, 9/1, *v*/*v*, 600 MHz]; Figure S26. ^13^C NMR spectrum of compound **6** [acetone-*d*_6_/D_2_O, 9/1, *v*/*v*, 151 MHz]; Figure S27. HSQC spectrum of compound **8** [acetone-*d*_6_/D_2_O, 9/1, *v*/*v*, 600 MHz]; Figure S28. HMBC spectrum of compound **8** [acetone-*d*_6_/D_2_O, 9/1, *v*/*v*, 600 MHz]; Figure S29. ^1^H NMR spectrum of compound **9** [acetone-*d*_6_/D_2_O, 9/1, *v*/*v*, 600 MHz]; Figure S30. ^13^C NMR spectrum of compound **9** [acetone-*d*_6_/D_2_O, 9/1, *v*/*v*, 151 MHz]; Figure S31. ^1^H NMR spectrum of compound **10** [acetone-*d*_6_/D_2_O, 9/1, *v*/*v*, 600 MHz]; Figure S32. ^1^H–^1^H COSY spectrum of compound **10** [acetone-*d*_6_/D_2_O, 9/1, *v*/*v*, 600 MHz]; Figure S33. ^13^C NMR spectrum of compound **10** [acetone-*d*_6_/D_2_O, 9/1, *v*/*v*, 151 MHz]; Figure S34. HSQC spectrum of compound **10** [acetone-*d*_6_/D_2_O, 9/1, *v*/*v*, 600 MHz]; Figure S35. HMBC spectrum of compound **10** [acetone-*d*_6_/D_2_O, 9/1, *v*/*v*, 600 MHz]; Figure S36. ^1^H NMR spectrum of compound 11 CD_3_OD, 100 MHz); Figure S37. ^13^C NMR spectrum of compound **11** (CD_3_OD, 100 MHz).
