# Supplementary material for: Nutritional, Antioxidant, Antimicrobial, and Anticholinesterase Properties of Phyllanthus emblica: A Study Supported by Spectroscopic and Computational Investigations
Source: Metabolites. 2023 Sep 14;13(9):1013. doi: 10.3390/metabo13091013 (PMC10537340; doi:10.3390/metabo13091013)
Supplement: Supplementary file 1 [file metabolites-13-01013-s001.zip › metabolites-2542705-supplementary.pdf]

metabolites

# Supplementary Materials

# Nutritional, antioxidant, antimicrobial, and anticholinesterase properties of *Phyllanthus emblica*: A study supported by spectroscopic and computational investigations

**Mohamed A. A. Orabi [1,*], Aso Hameed Hasan [2,3], Sameh F. AbouZid [4], Dalia El Amir [4,*], Mona H. Hetta [5], Ahmed Abdullah Al Awadh [6], Omaish Salman Alqahtani [1], Tsutomu Hatano [7] and Mohamed A. El-Shanawany [8]**

[1] Department of Pharmacognosy, College of Pharmacy, Najran University 1988, Najran 66454, Saudi Arabia; mohamedorabi@azhar.edu.eg (MAAO); osalqahtani@nu.edu.sa (OSA)
[2] Department of Chemistry, College of Science, University of Garmian, Kalar 46021, Kurdistan Region-Iraq, Iraq; aso.hameed@garmian.edu.krd
[3] Department of Chemistry, Faculty of Science, Universiti Teknologi Malaysia, Johor Bahru 81310, Johor, Malaysia
[4] Department of Pharmacognosy, Faculty of Pharmacy, Beni-Suef University, Beni-Suef 62514, Egypt; sameh.zaid@pharm.bsu.edu.eg (S.F.A.), dalia.elamir@pharm.bsu.edu.eg (DEA)
[5] Department of Pharmacognosy, Faculty of Pharmacy, Fayoum University - 63514 Fayoum, Egypt; monahetta@gmail.com
[6] Department of Clinical Laboratory Sciences, Faculty of Applied Medical Sciences, Najran University, 1988, Najran 66454, Saudi Arabia; aaalawadh@nu.edu.sa
[7] Graduate School of Medicine, Dentistry and Pharmaceutical Sciences, Okayama University, Tsushima, Okayama 700-8530, Japan; hatano-t@cc.okayama-u.ac.jp
[8] Department of Pharmacognosy, Faculty of Pharmacy, Badr University in Cairo (BUC), Badr City 11829, Egypt; mohamed.elshanawani@pharm.aun.edu.eg

* Correspondence: mohamedorabi@azhar.edu.eg (MAAO), Dalia.elamir@pharm.bsu.edu.eg (DE)

**List of supplementary materials**


| **Content** | **Page** |
|---|---|
| **Figure S1.** $^{1}$H NMR spectrum of compound **1** (DMSO-$d_6$, 400 MHz) | **S4** |
| **Figure S2.** $^{13}$C NMR spectrum of compound **1** (DMSO-$d_6$, 100 MHz) | **S5** |
| **Figure S3.** $^{1}$H NMR spectrum of compound **2** ($CD_3OD$, 400MHz) | **S6** |
| **Figure S4.** DPTQ NMR spectrum of compound **2** ($CD_3OD$, 100MHz) | **S7** |
| **Figure S5.** $^{1}$H NMR spectrum of compound **3** [acetone-$d_6$/$D_2O$, 9/1, *v*/*v*, 600 MHz] | **S8** |
| **Figure S6.** $^{13}$C NMR spectrum of compound **3** [acetone-$d_6$/$D_2O$, 9/1, *v*/*v*, 151 MHz] | **S9** |
| **Figure S7.** HSQC spectrum of compound **3** [acetone-$d_6$/$D_2O$, 9/1, *v*/*v*, 600 MHz] | **S10** |
| **Figure S8.** HMBC spectrum of compound **3** [acetone-$d_6$/$D_2O$, 9/1, *v*/*v*, 600 MHz] | **S11** |
| **Figure S9.** $^{1}$H NMR spectrum of compound **4** [acetone-$d_6$/$D_2O$, 9/1, *v*/*v*, 600 MHz] | **S12** |
| **Figure S10.** $^{13}$C NMR spectrum of compound **4** [acetone-$d_6$/$D_2O$, 9/1, *v*/*v*, 151 MHz] | **S13** |
| **Figure S11.** $^{1}$H-$^{1}$H COSY spectrum of compound **4** [acetone-$d_6$/$D_2O$, 9/1, *v*/*v*, 600 MHz] | **S14** |
| **Figure S12.** HSQC spectrum of compound **4** [acetone-$d_6$/$D_2O$, 9/1, *v*/*v*, 600 MHz] | **S15** |
| **Figure S13.** HMBC spectrum of compound **4** [acetone-$d_6$/$D_2O$, 9/1, *v*/*v*, 600 MHz] | **S16** |
| **Figure S14.** $^{1}$H NMR spectrum of compound **5** [acetone-$d_6$/$D_2O$, 9/1, *v*/*v*, 600 MHz] | **S17** |
| **Figure S15.** $^{1}$H–$^{1}$H COSY spectrum of compound **5** [acetone-$d_6$/$D_2O$, 9/1, *v*/*v*, 600 MHz] | **S18** |
| **Figure S16.** $^{1}$H NMR spectrum of compound **6** [acetone-$d_6$/$D_2O$, 9/1, *v*/*v*, 600 MHz] | **S19** |
| **Figure S17.** $^{13}$C NMR spectrum of compound **6** [acetone-$d_6$/$D_2O$, 9/1, *v*/*v*, 151 MHz] | **S20** |
| **Figure S18.** HSQC spectrum of compound **6** [acetone-$d_6$/$D_2O$, 9/1, *v*/*v*, 600 MHz] | **S21** |
| **Figure S19.** HMBC spectrum of compound **6** [acetone-$d_6$/$D_2O$, 9/1, *v*/*v*, 600 MHz] | **S22** |
| **Figure S20.** $^{1}$H NMR spectrum of compound **7** [acetone-$d_6$/$D_2O$, 9/1, *v*/*v*, 600 MHz] | **S23** |
| **Figure S21.** $^{1}$H-$^{1}$H COSY spectrum of compound **7** [acetone-$d_6$/$D_2O$, 9/1, *v*/*v*, 600 MHz] | **S24** |
| **Figure S22.** $^{13}$C NMR spectrum of compound **7** [acetone-$d_6$/$D_2O$, 9/1, *v*/*v*, 151 MHz] | **S25** |
| **Figure S23.** HSQC spectrum of compound **7** [acetone-$d_6$/$D_2O$, 9/1, *v*/*v*, 600 MHz] | **S26** |
| **Figure S24.** HMBC spectrum of compound **7** [acetone-$d_6$/$D_2O$, 9/1, *v*/*v*, 600 MHz] | **S27** |
| **Figure S25.** $^{1}$H NMR spectrum of compound **8** [acetone-$d_6$/$D_2O$, 9/1, *v*/*v*, 600 MHz] | **S28** |
| **Figure S26.** $^{13}$C NMR spectrum of compound **6** [acetone-$d_6$/$D_2O$, 9/1, *v*/*v*, 151 MHz] | **S29** |
| **Figure S27.** HSQC spectrum of compound **8** [acetone-$d_6$/$D_2O$, 9/1, *v*/*v*, 600 MHz] | **S30** |
| **Figure S28.** HMBC spectrum of compound **8** [acetone-$d_6$/$D_2O$, 9/1, *v*/*v*, 600 MHz] | **S31** |
| **Figure S29.** $^{1}$H NMR spectrum of compound **9** [acetone-$d_6$/$D_2O$, 9/1, *v*/*v*, 600 MHz] | **S32** |
| **Figure S30.** $^{13}$C NMR spectrum of compound **9** [acetone-$d_6$/$D_2O$, 9/1, *v*/*v*, 151 MHz] | **S33** |

**Figure S31.** $^1H$ NMR spectrum of compound **10** [acetone-$d_6$/$D_2O$, 9/1, *v*/*v*, 600 MHz] **S34**

**Figure S32.** $^1H$–$^1H$ COSY spectrum of compound **10** [acetone-$d_6$/$D_2O$, 9/1, *v*/*v*, 600 MHz] **S35**

**Figure S33.** $^{13}C$ NMR spectrum of compound **10** [acetone-$d_6$/$D_2O$, 9/1, *v*/*v*, 151 MHz] **S36**

**Figure S34.** HSQC spectrum of compound **10** [acetone-$d_6$/$D_2O$, 9/1, *v*/*v*, 600 MHz] **S37**

**Figure S35.** HMBC spectrum of compound **10** [acetone-$d_6$/$D_2O$, 9/1, *v*/*v*, 600 MHz] **S38**

**Figure S36.** $^1H$ NMR spectrum of compound **11** $CD_3OD$, 100 MHz) **S39**

**Figure S37.** $^{13}C$ NMR spectrum of compound **11** ($CD_3OD$, 100 MHz) **S40**

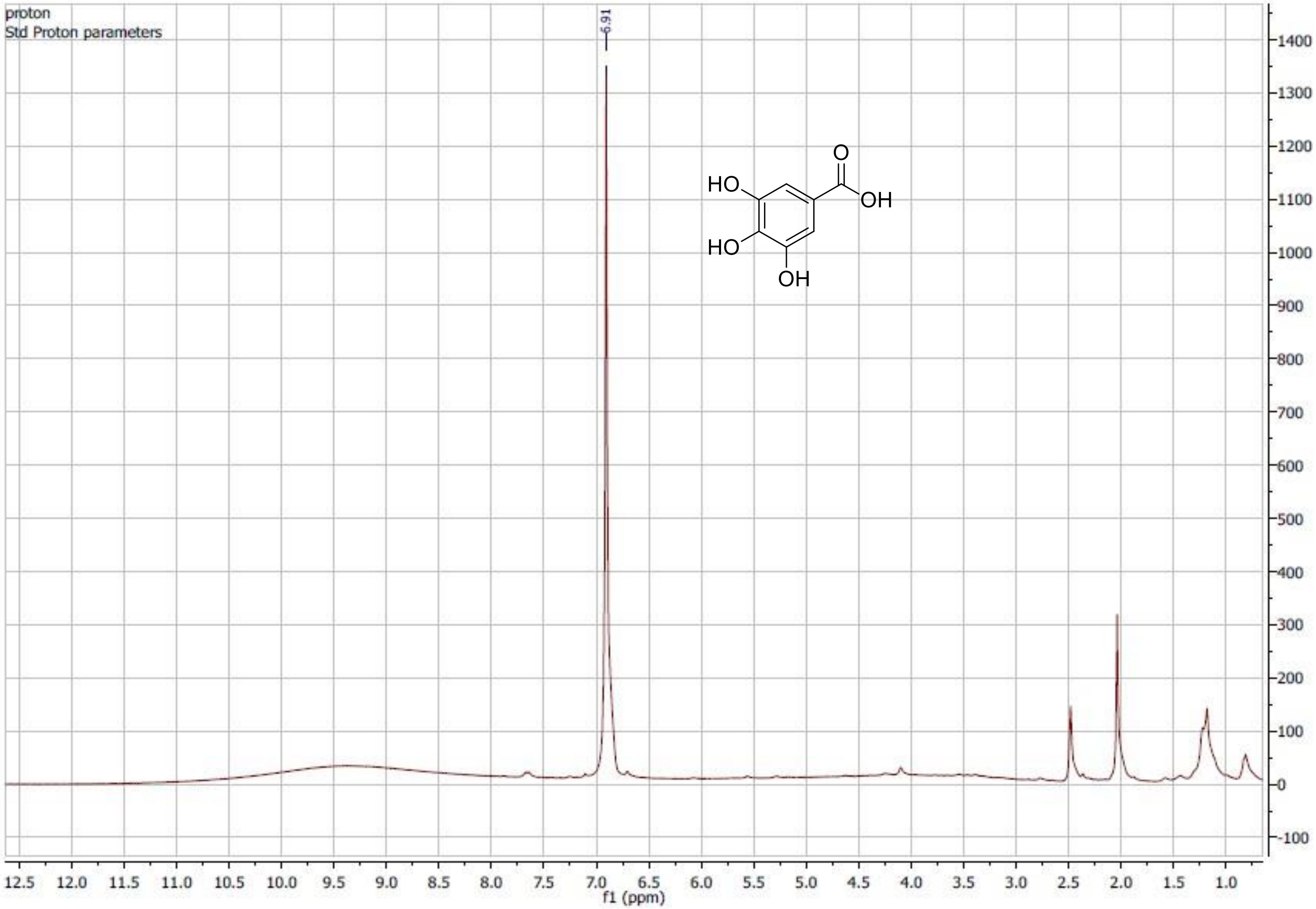


**Figure S1.** $^1$H NMR spectrum of compound **1** (DMSO-$d_6$, 400 MHz)

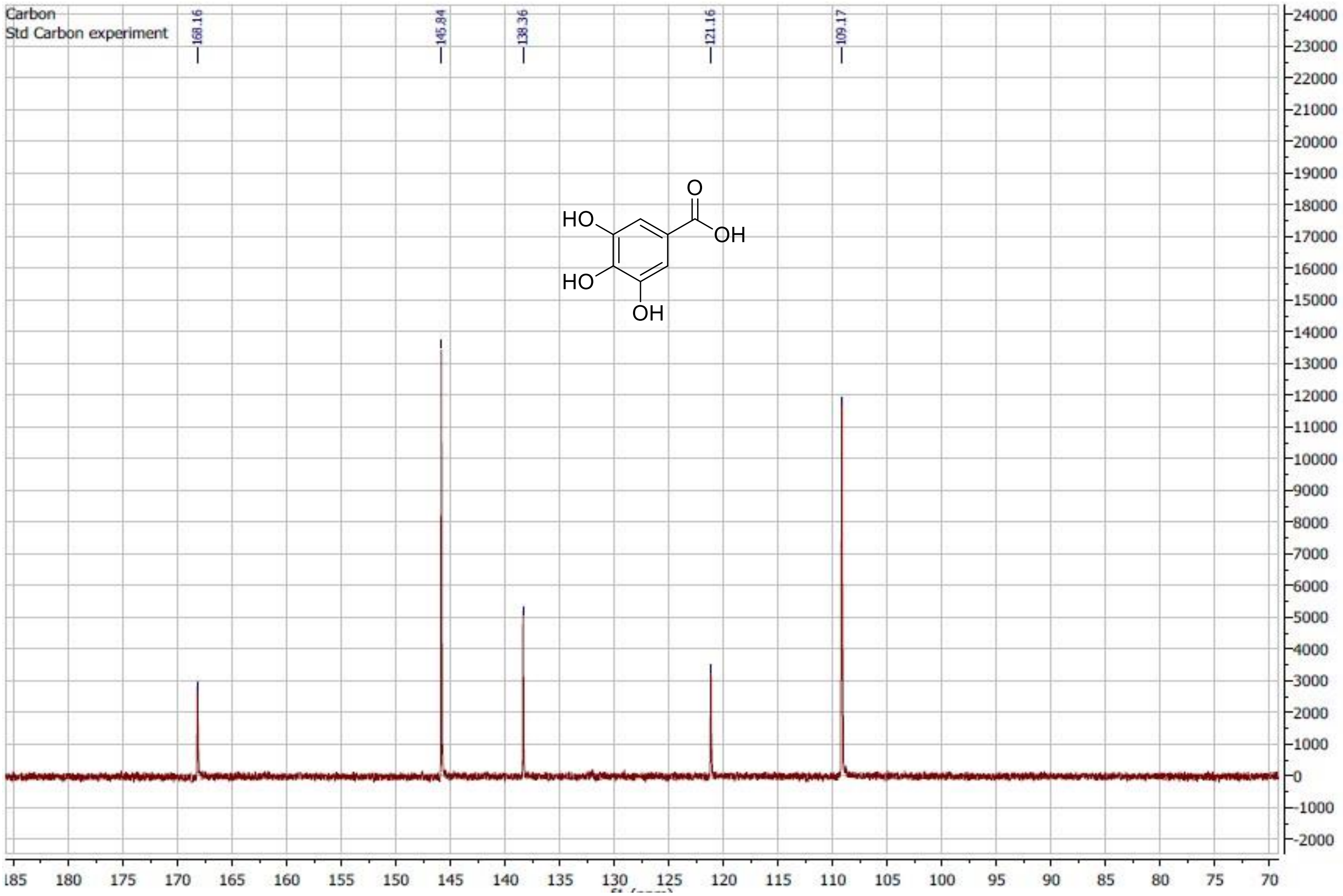


**Figure S2.** $^{13}C$ NMR spectrum of compound **1** (DMSO-$d_6$, 100 MHz)

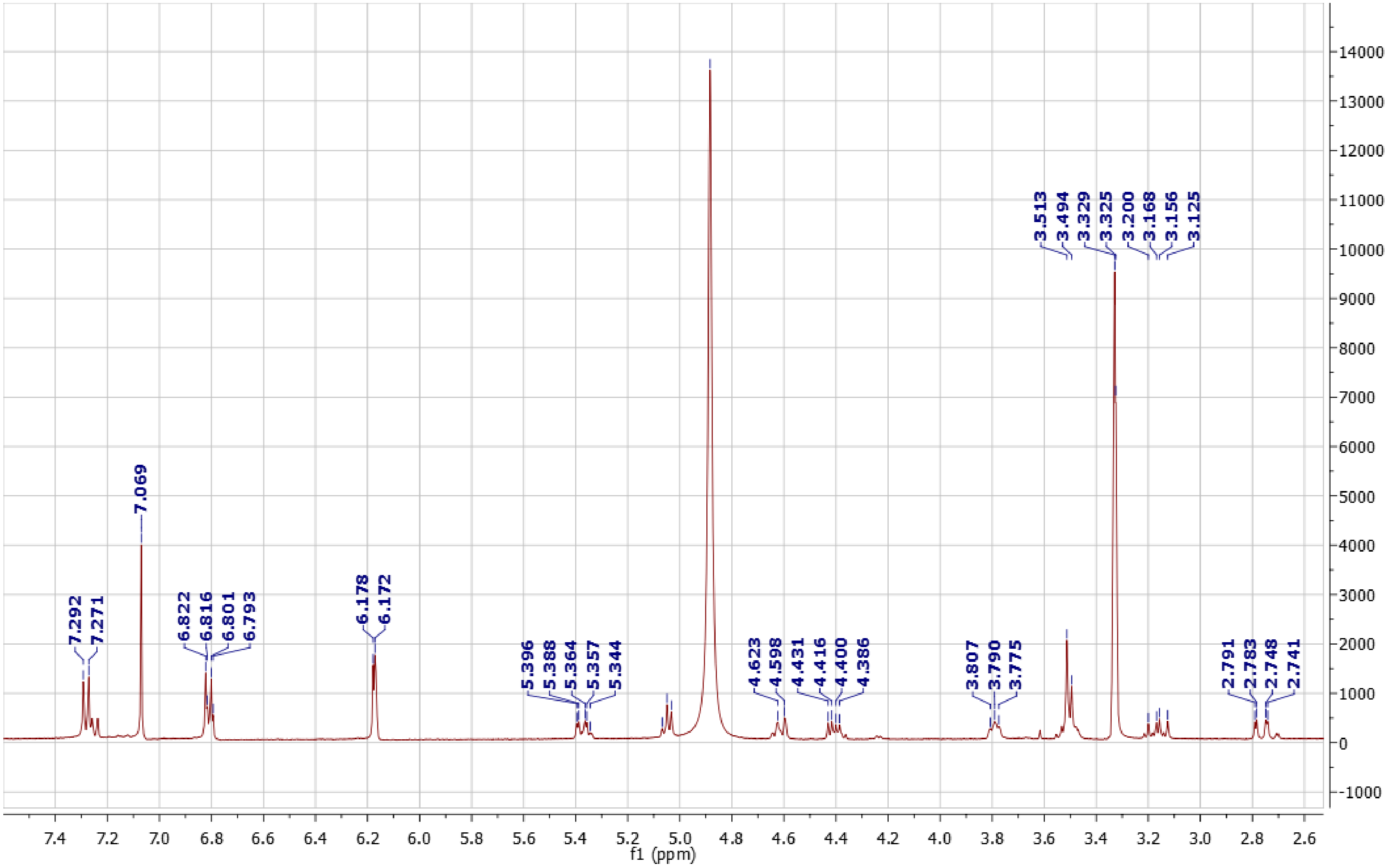


**Figure S3.**$^{1}$H NMR spectrum of compound **2** ($CD_3OD$, 400MHz)

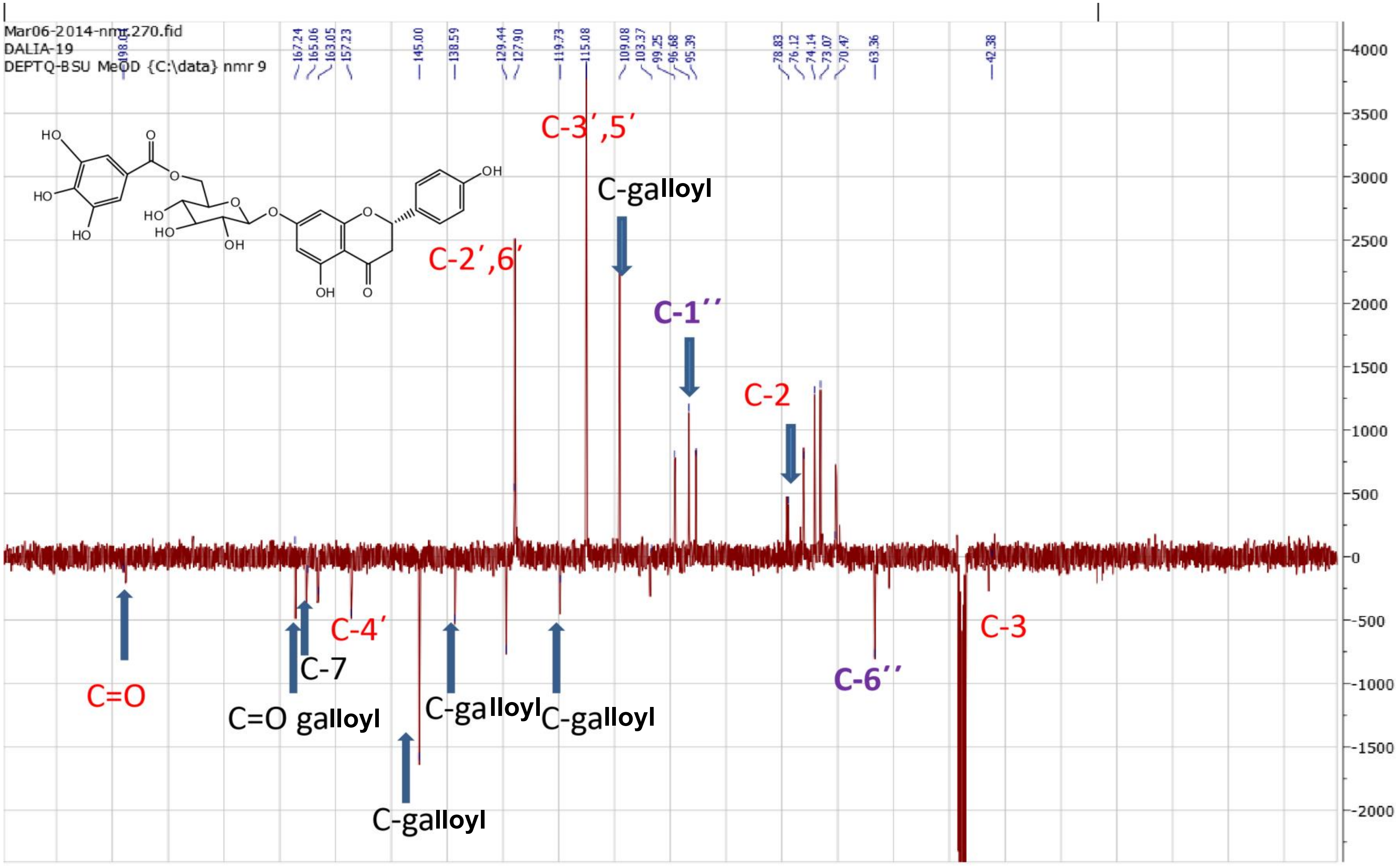


**Figure S4.** DPTQ NMR spectrum of compound **2** ($CD_3OD$, 100 MHz)

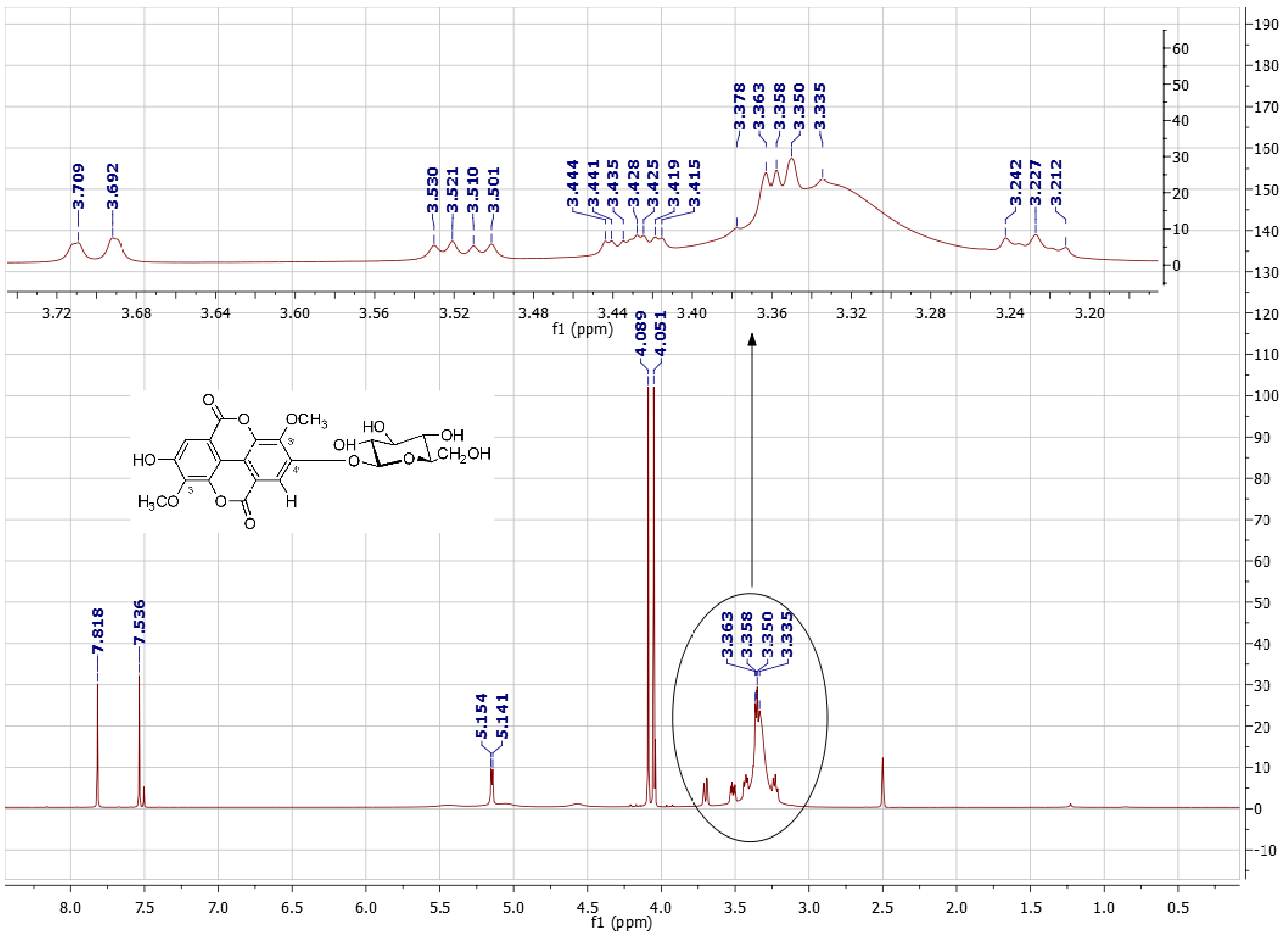

**Figure S5.** $^{1}$H NMR spectrum of compound **3** [acetone-$d_6$/$D_2O$, 9/1, *v/v*, 600 MHz]

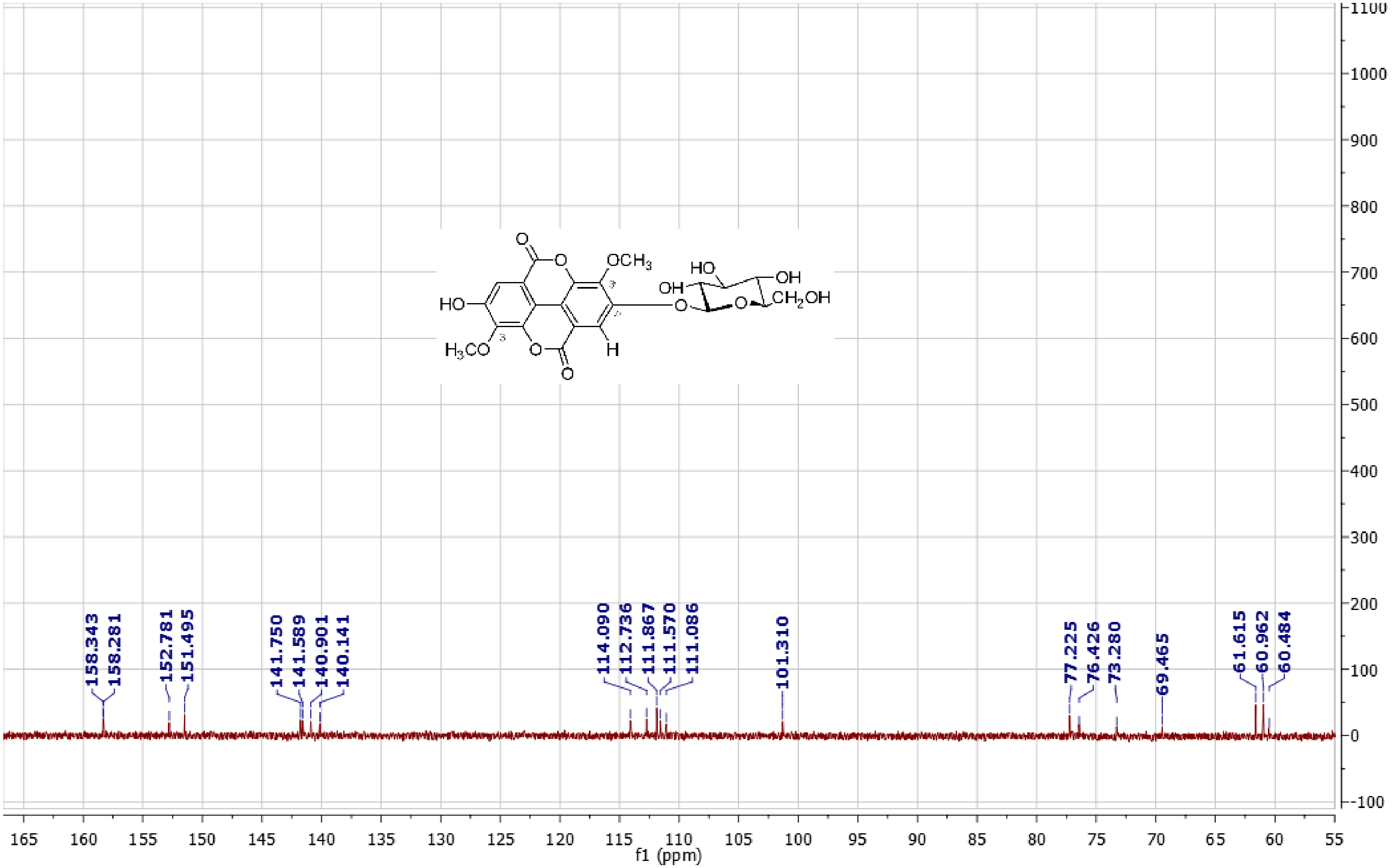


**Figure S6.** $^{13}C$ NMR spectrum of compound **3** [acetone-$d_6$/$D_2O$, 9/1, *v/v*, 151 MHz]

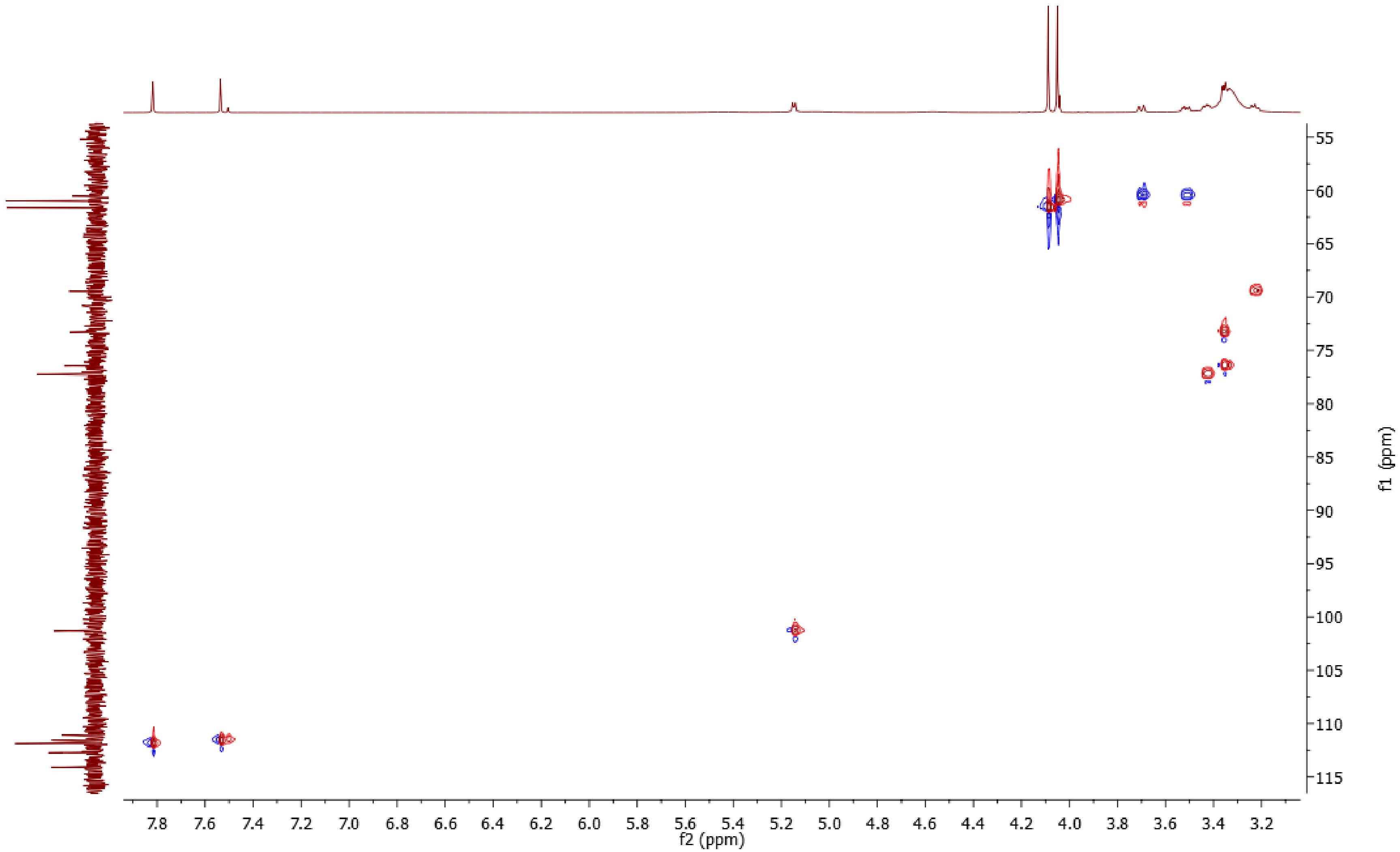


**Figure S7.** HSQC spectrum of compound **3** [acetone-$d_6$/$D_2O$, 9/1, *v/v*, 600 MHz]

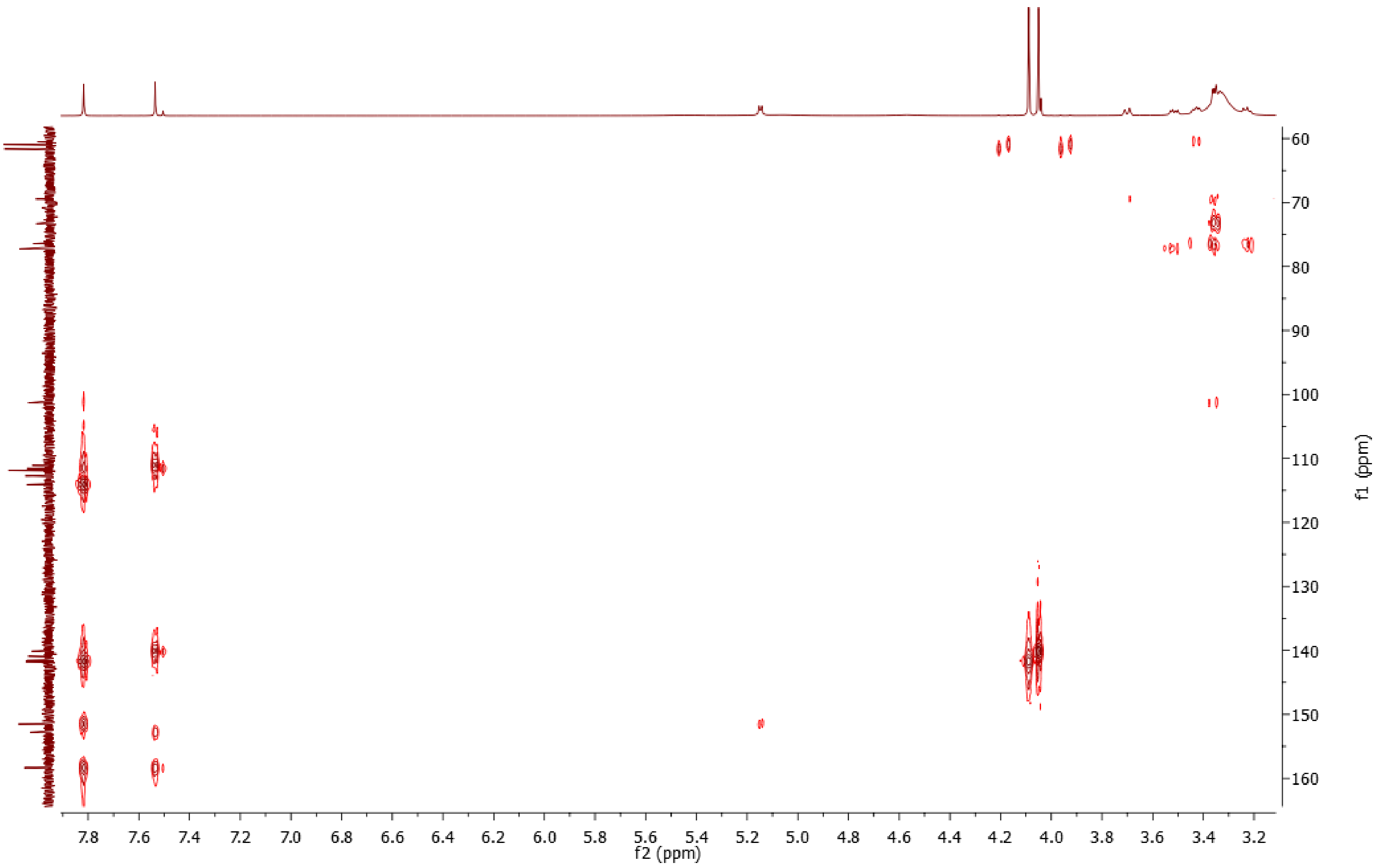


**Figure S8.** HMBC spectrum of compound **3** [acetone-$d_6$/$D_2O$, 9/1, *v/v*, 600 MHz]

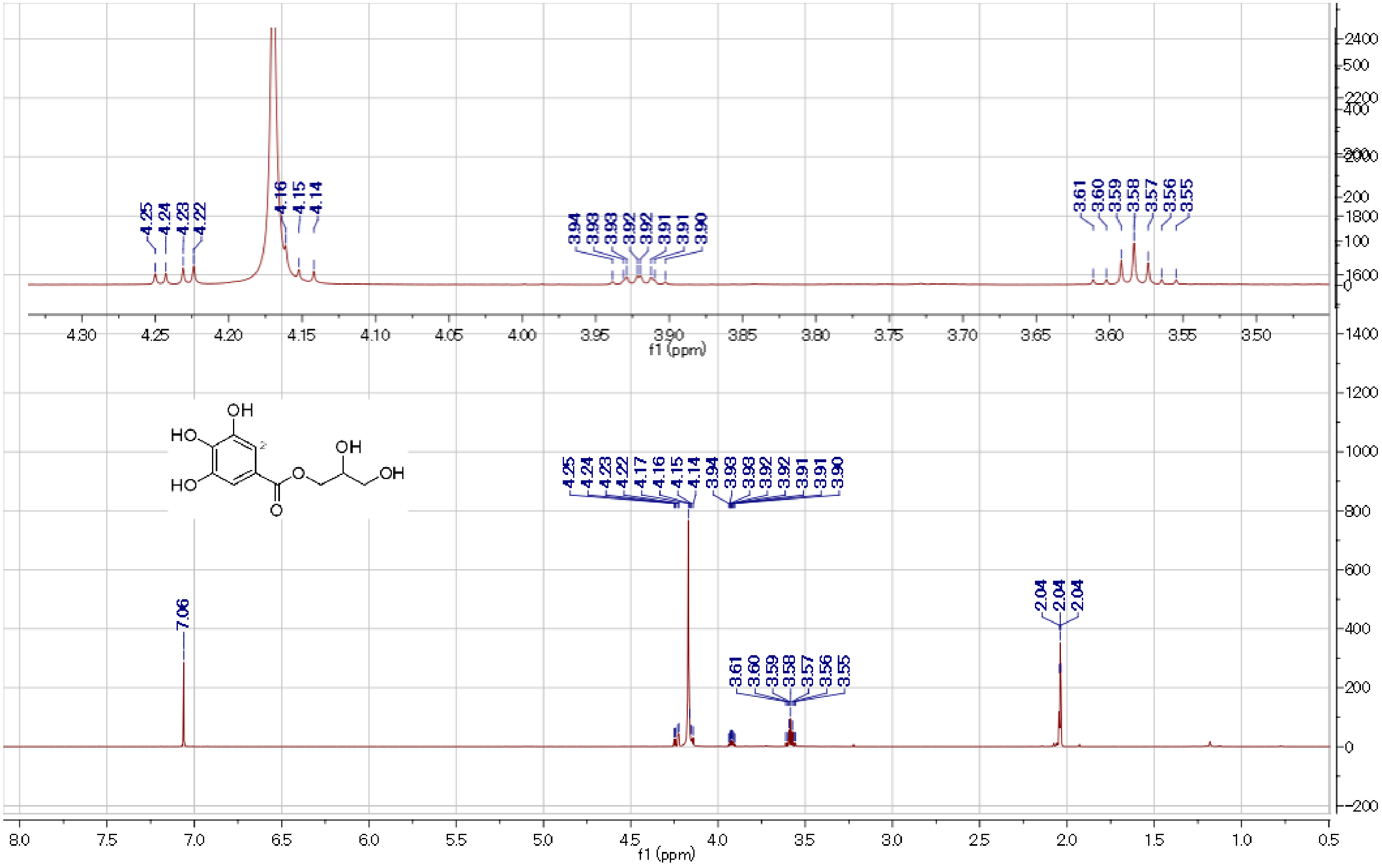


**Figure S9.** $^{1}H$ NMR spectrum of compound **4** [acetone-$d_6$/$D_2O$, 9/1, *v*/*v*, 600 MHz]

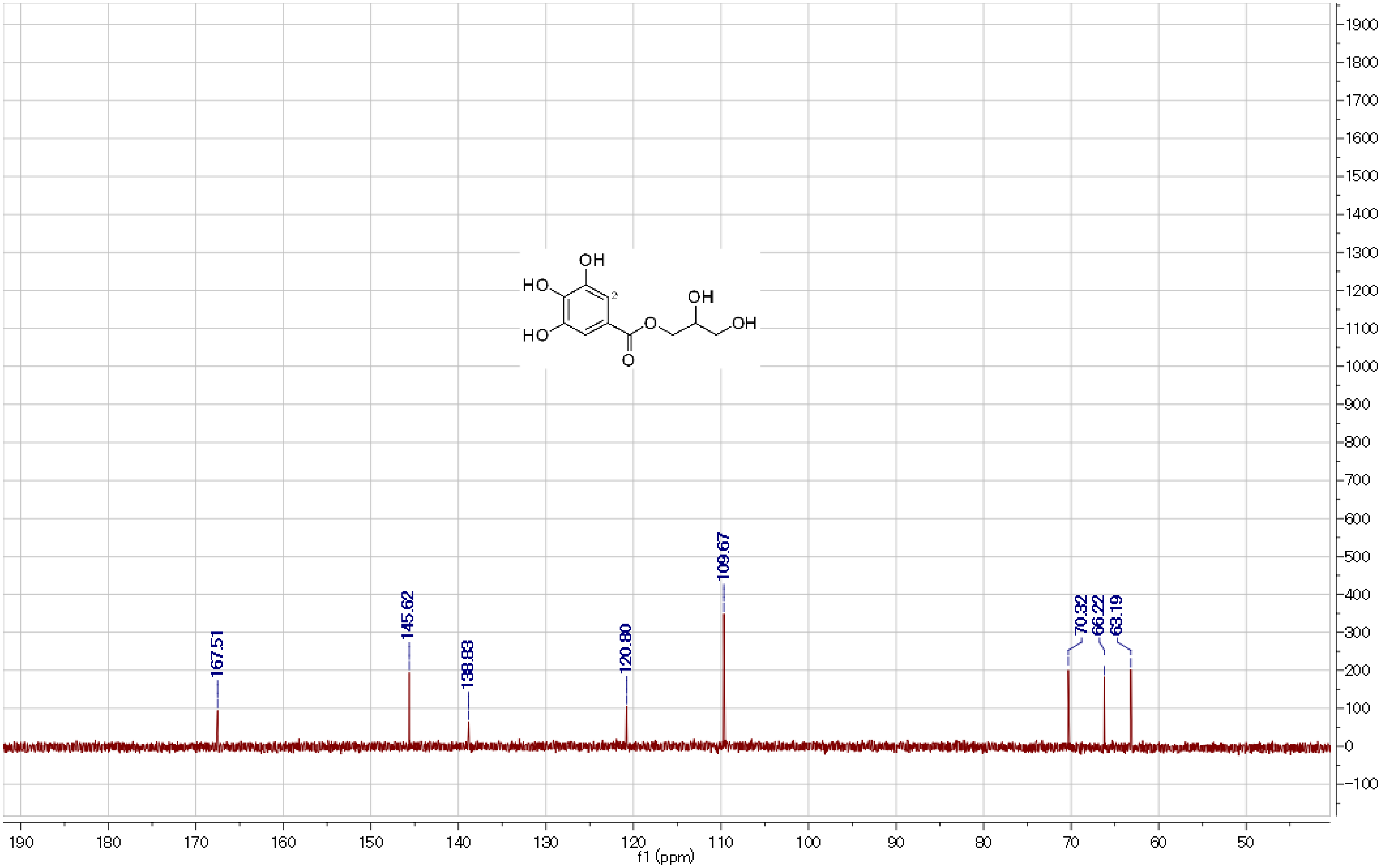


**Figure S10.** $^{13}$C NMR spectrum of compound **4** [acetone-$d_6$/$D_2O$, 9/1, *v/v*, 151 MHz]

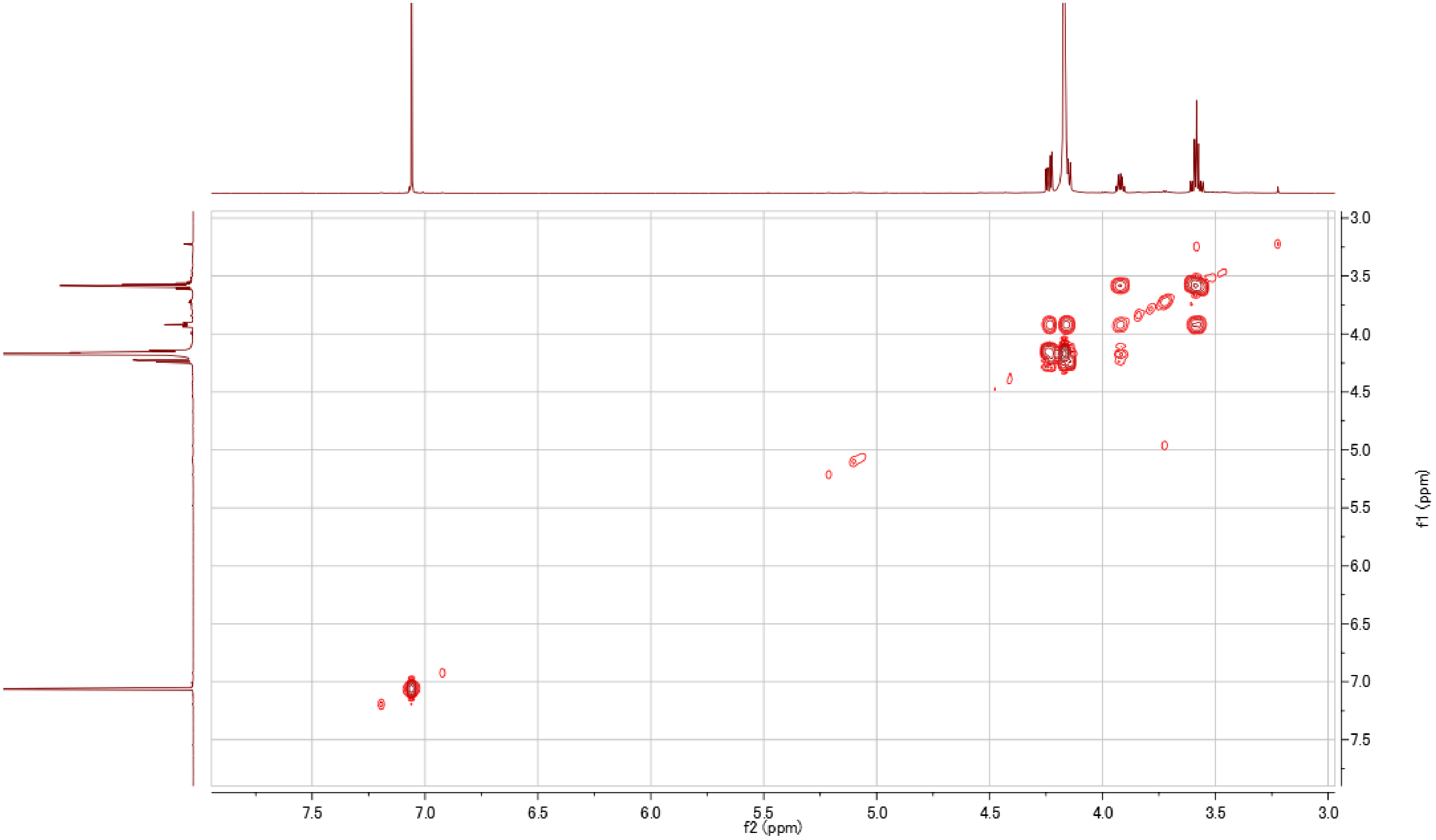


**Figure S11.** $^1H$-$^1H$ COSY spectrum of compound **4** [acetone-$d_6$/$D_2O$, 9/1, *v/v*, 600 MHz]

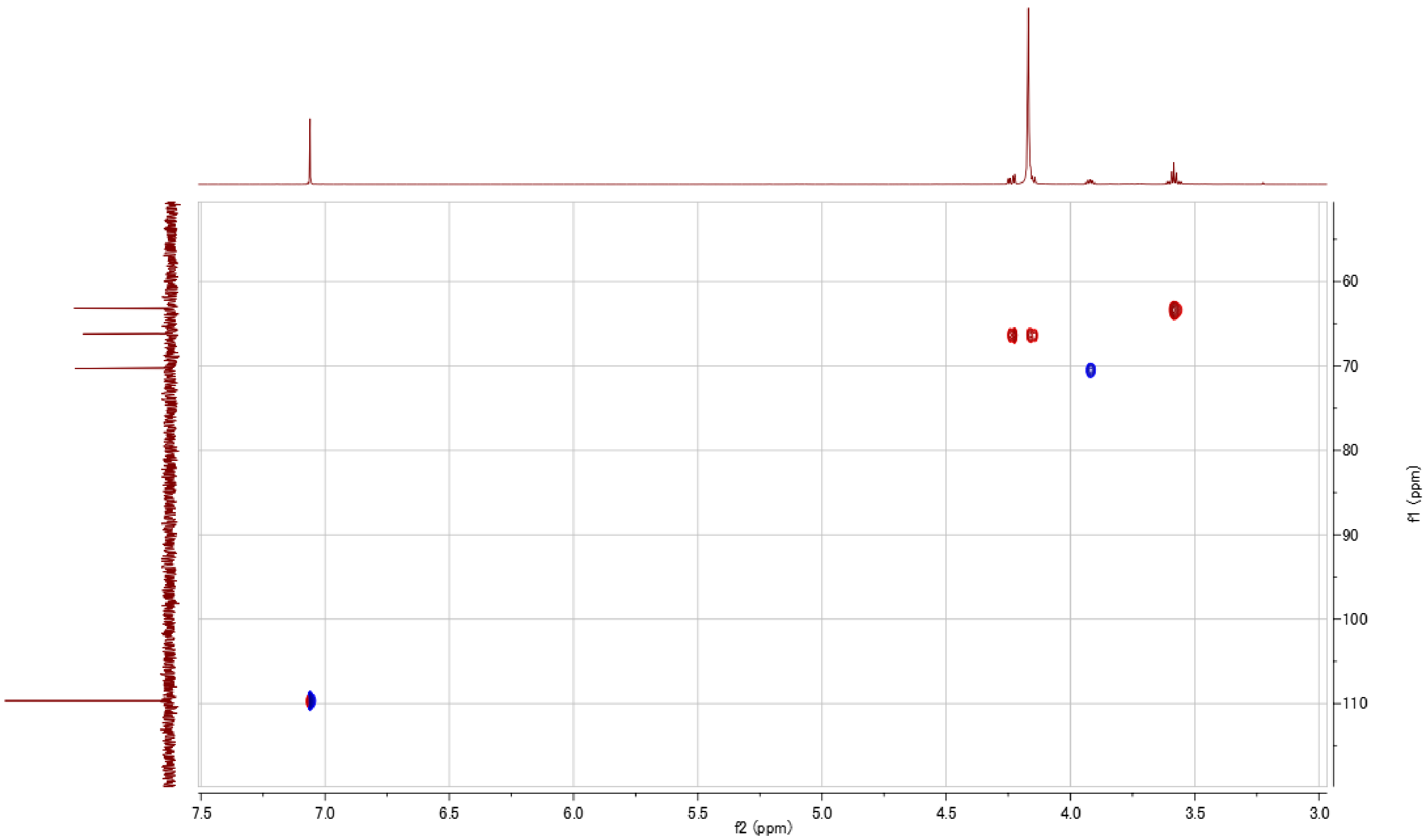


**Figure S12.** HSQC spectrum of compound **4** [acetone-$d_6$/$D_2O$, 9/1, *v/v*, 600 MHz]

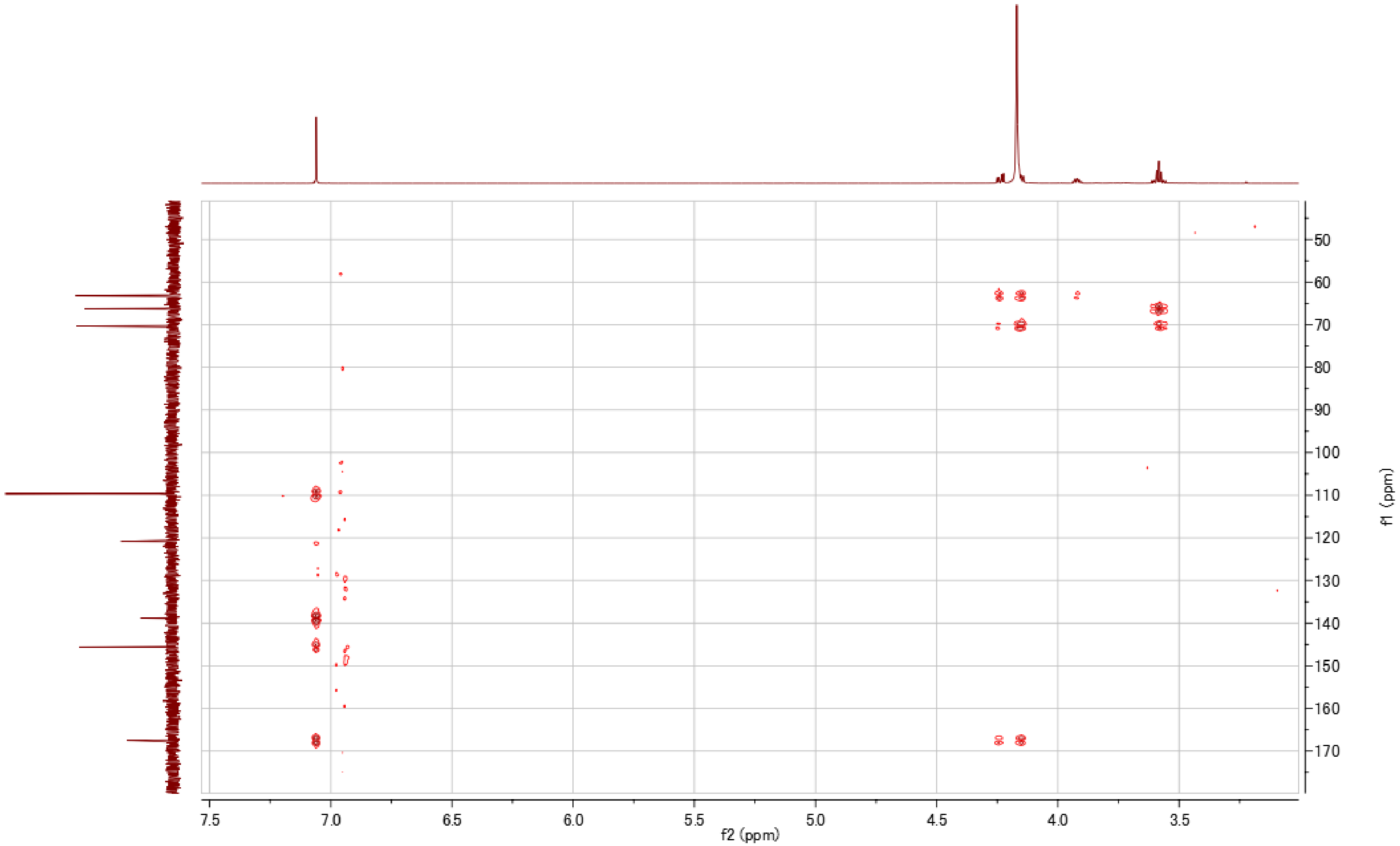


**Figure S13.** HMBC spectrum of compound **4** [acetone-$d_6$/$D_2O$, 9/1, *v/v*, 600 MHz]

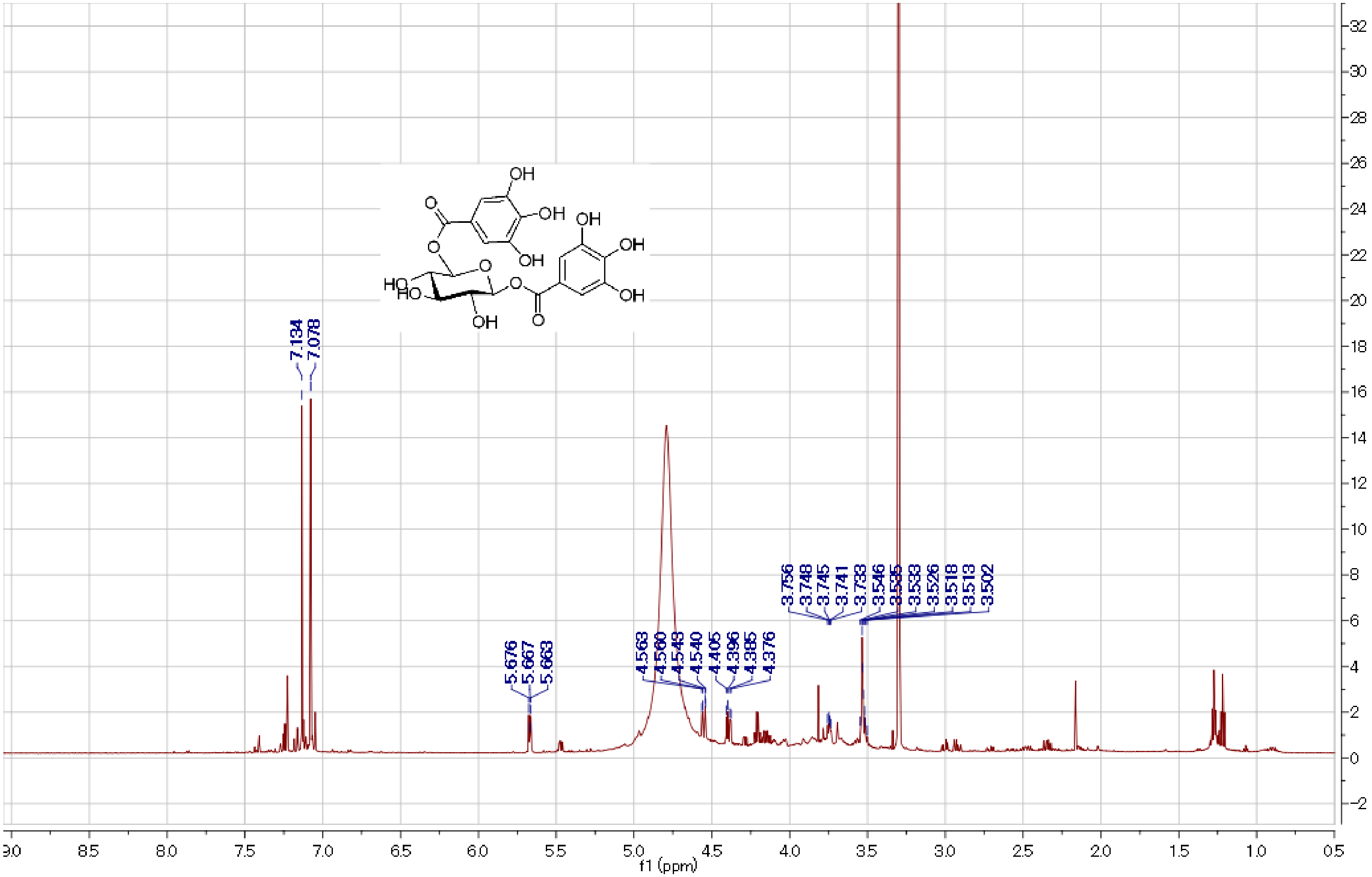

**Figure S14.** $^{1}$H NMR spectrum of compound **5** [acetone-$d_6$/$D_2O$, 9/1, *v/v*, 600 MHz]

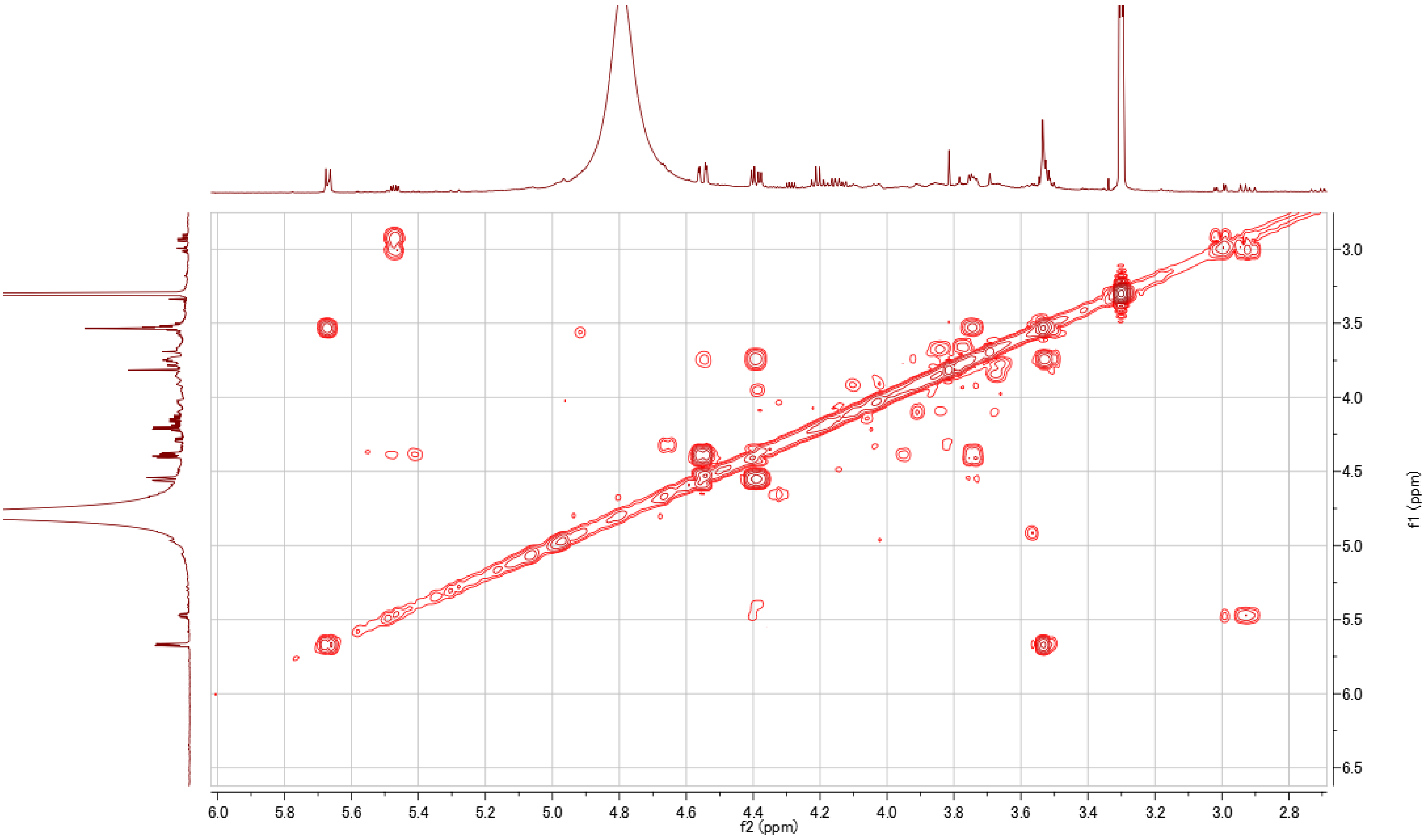


**Figure S15.** $^{1}H$–$^{1}H$ COSY spectrum of compound **5** [acetone-$d_6$/$D_2O$, 9/1, *v*/*v*, 600 MHz]

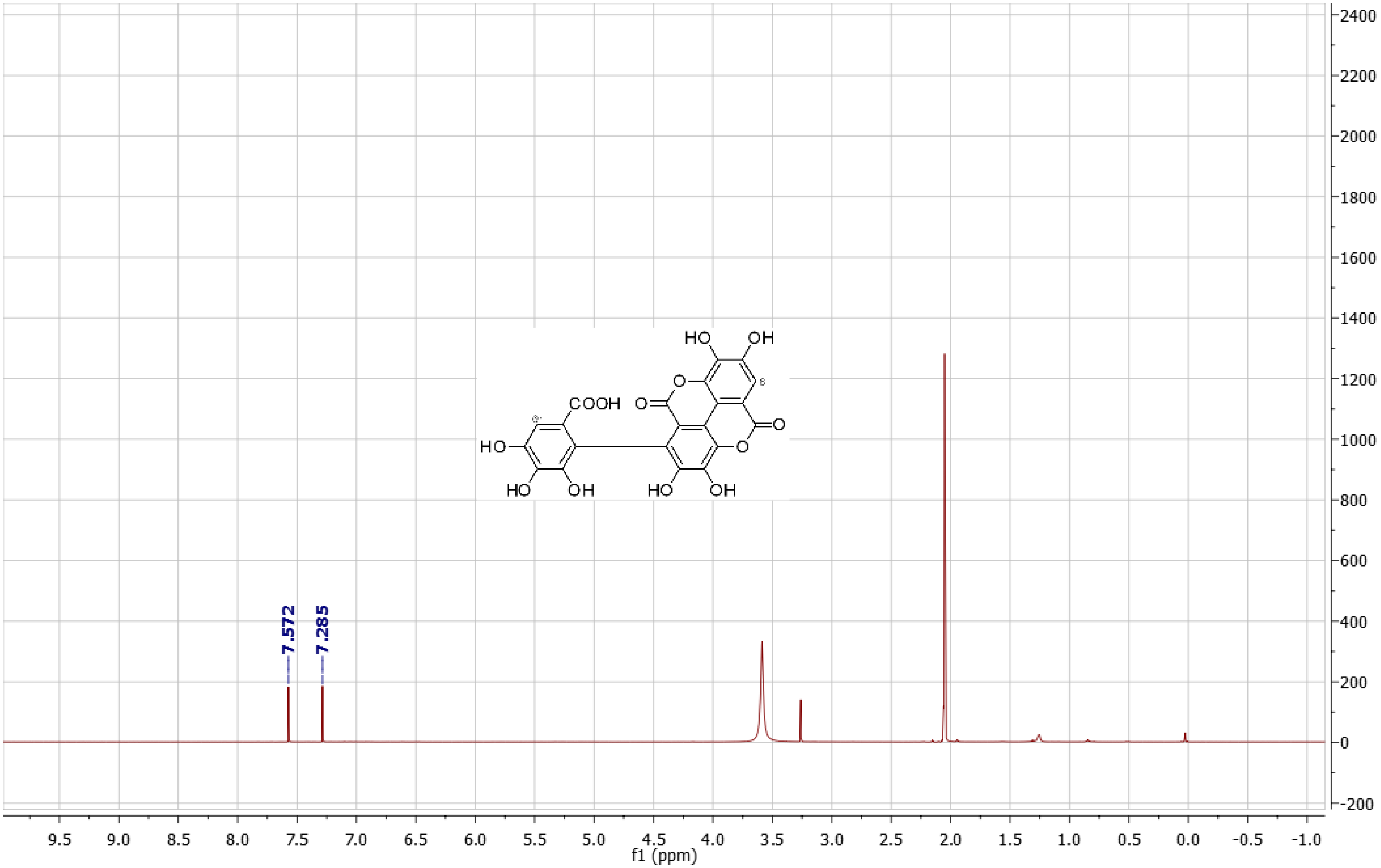


**Figure S16.** $^{1}$H NMR spectrum of compound **6** [acetone-$d_6$/$D_2O$, 9/1, *v/v*, 600 MHz]

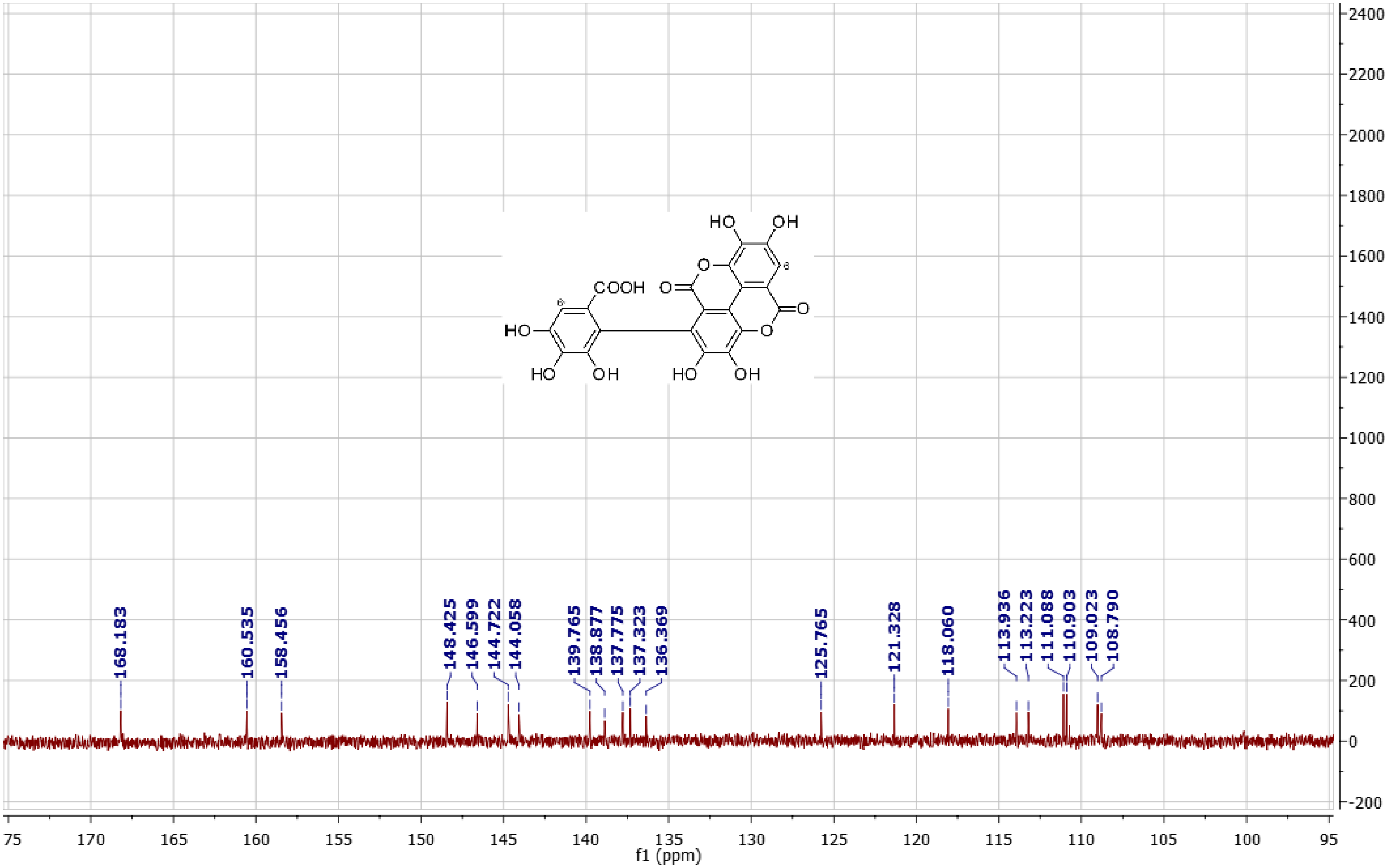


**Figure S17.** $^{13}C$ NMR spectrum of compound **6** [acetone-$d_6$/$D_2O$, 9/1, *v/v*, 151 MHz]

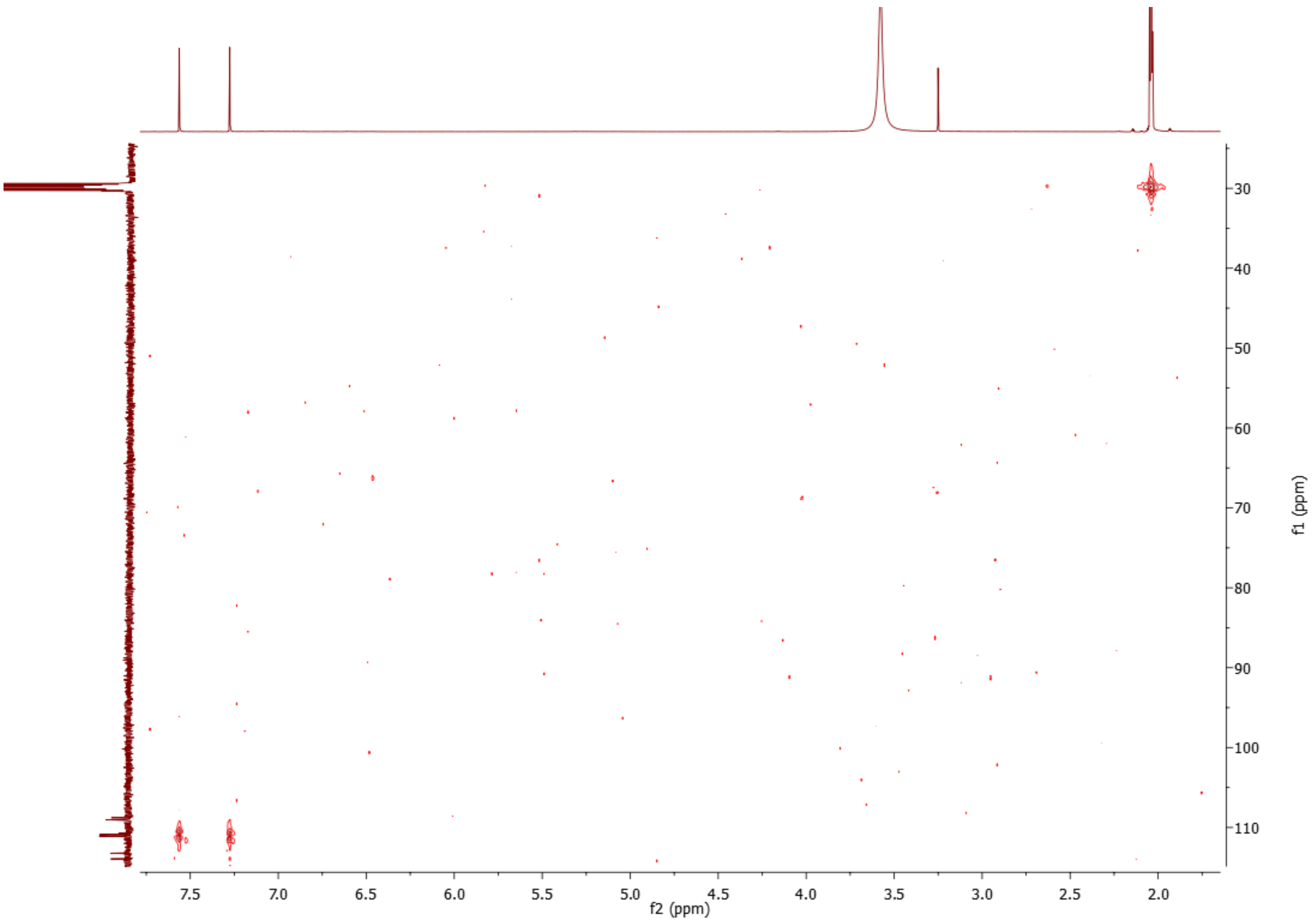


**Figure S18.** HSQC spectrum of compound **6** [acetone-$d_6$/$D_2O$, 9/1, *v/v*, 600 MHz]

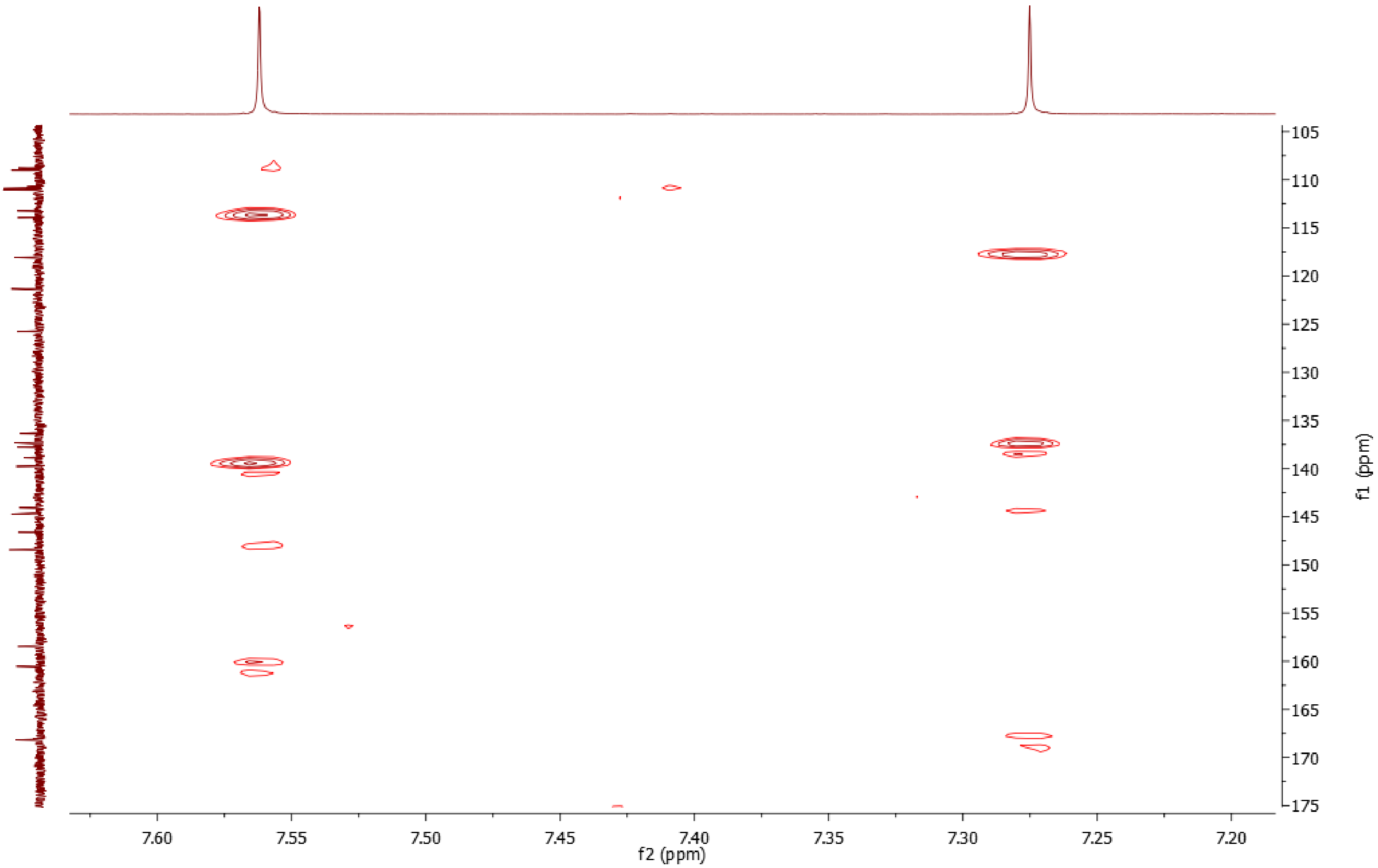


**Figure S19.** HMBC spectrum of compound **6** [acetone-$d_6$/$D_2O$, 9/1, *v/v*, 600 MHz]

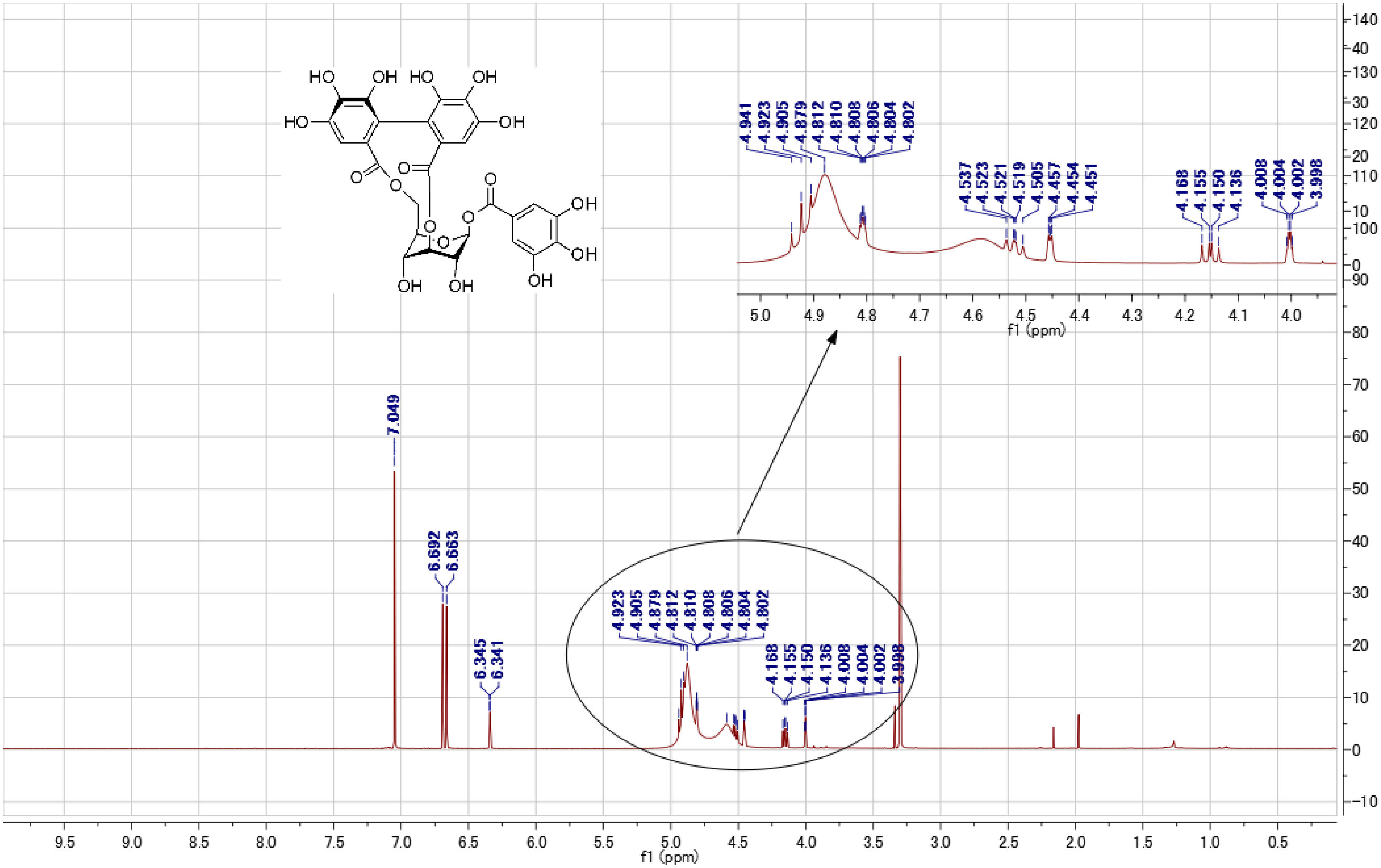

**Figure S20.** $^{1}$H NMR spectrum of compound **7** [acetone-$d_6$/$D_2O$, 9/1, *v/v*, 600 MHz]

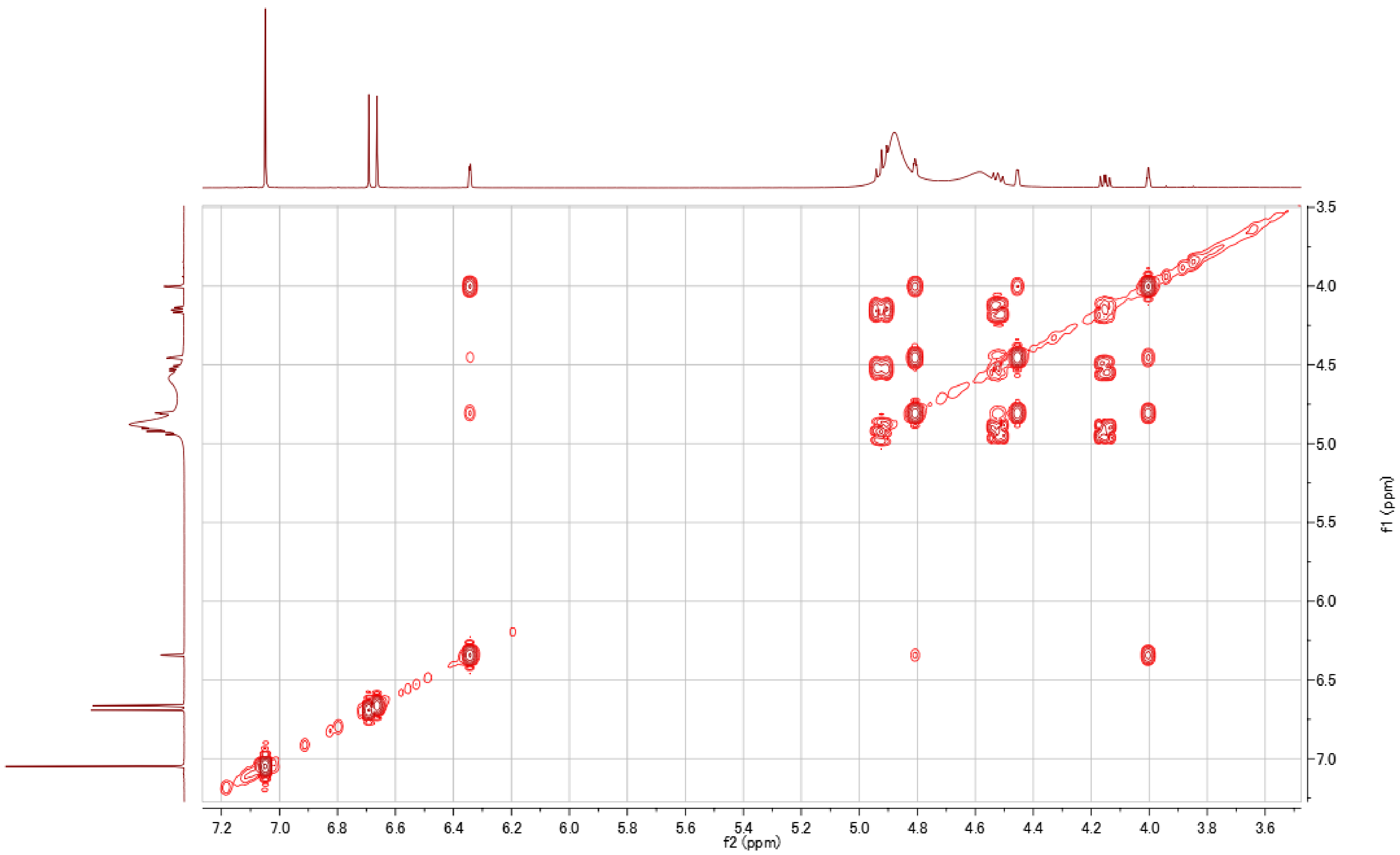


**Figure S21.** $^{1}H$-$^{1}H$ COSY spectrum of compound **7** [acetone-$d_6$/$D_2O$, 9/1, *v/v*, 600 MHz]

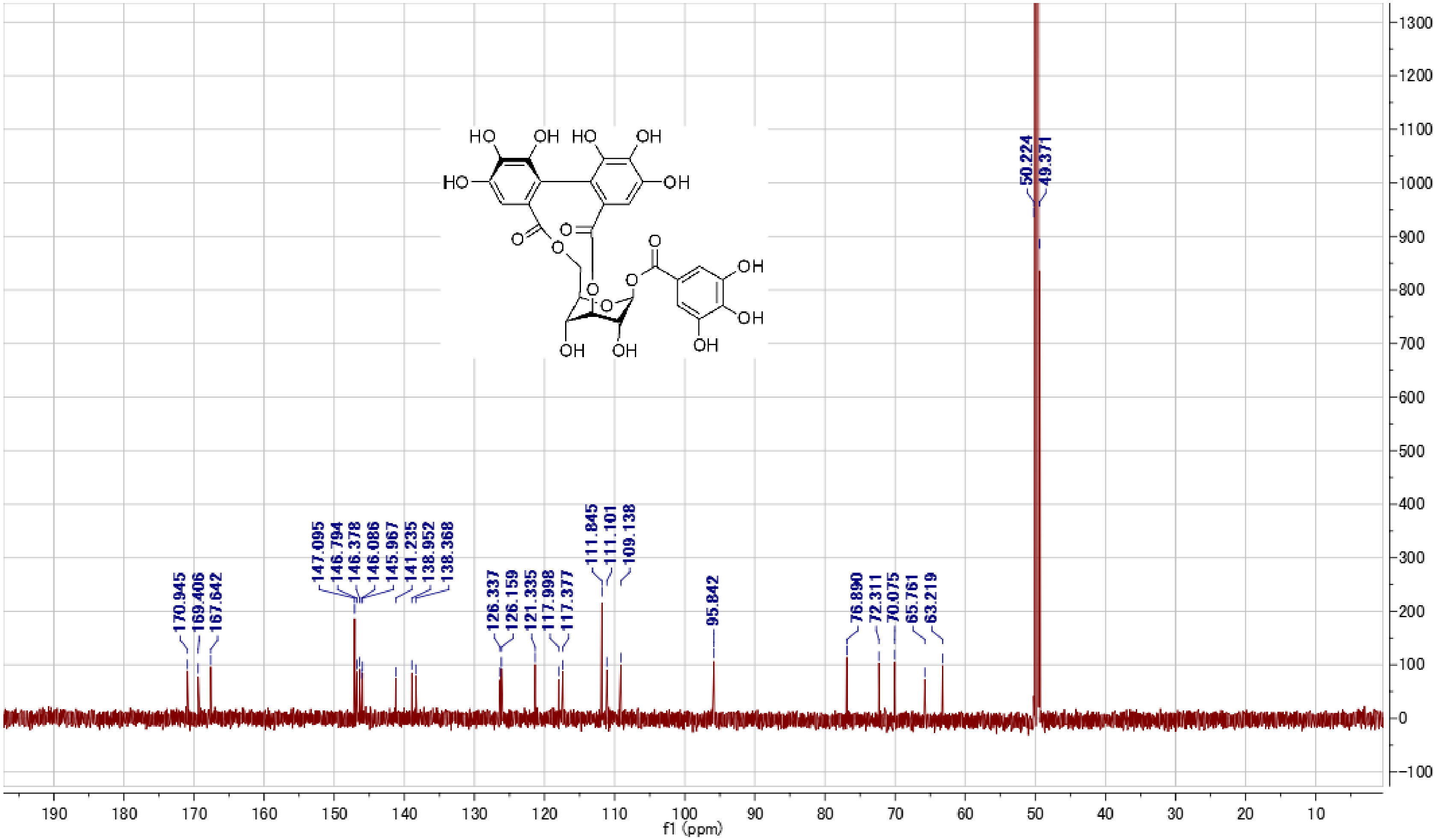


**Figure S22.** $^{13}$C NMR spectrum of compound **7** [acetone-$d_6$/$D_2O$, 9/1, *v/v*, 151 MHz]

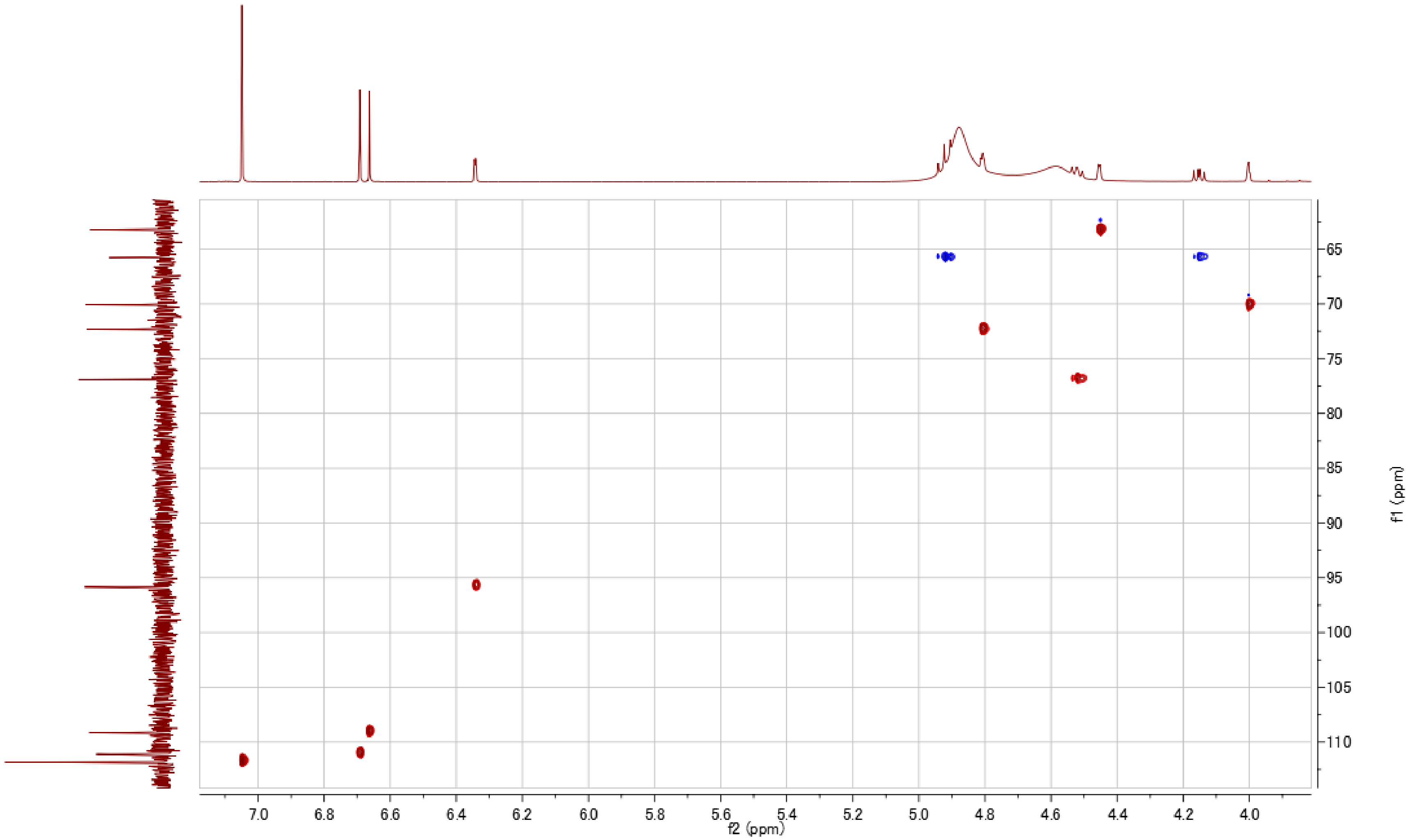


**Figure S23.** HSQC spectrum of compound **7** [acetone-$d_6$/$D_2O$, 9/1, *v/v*, 600 MHz]

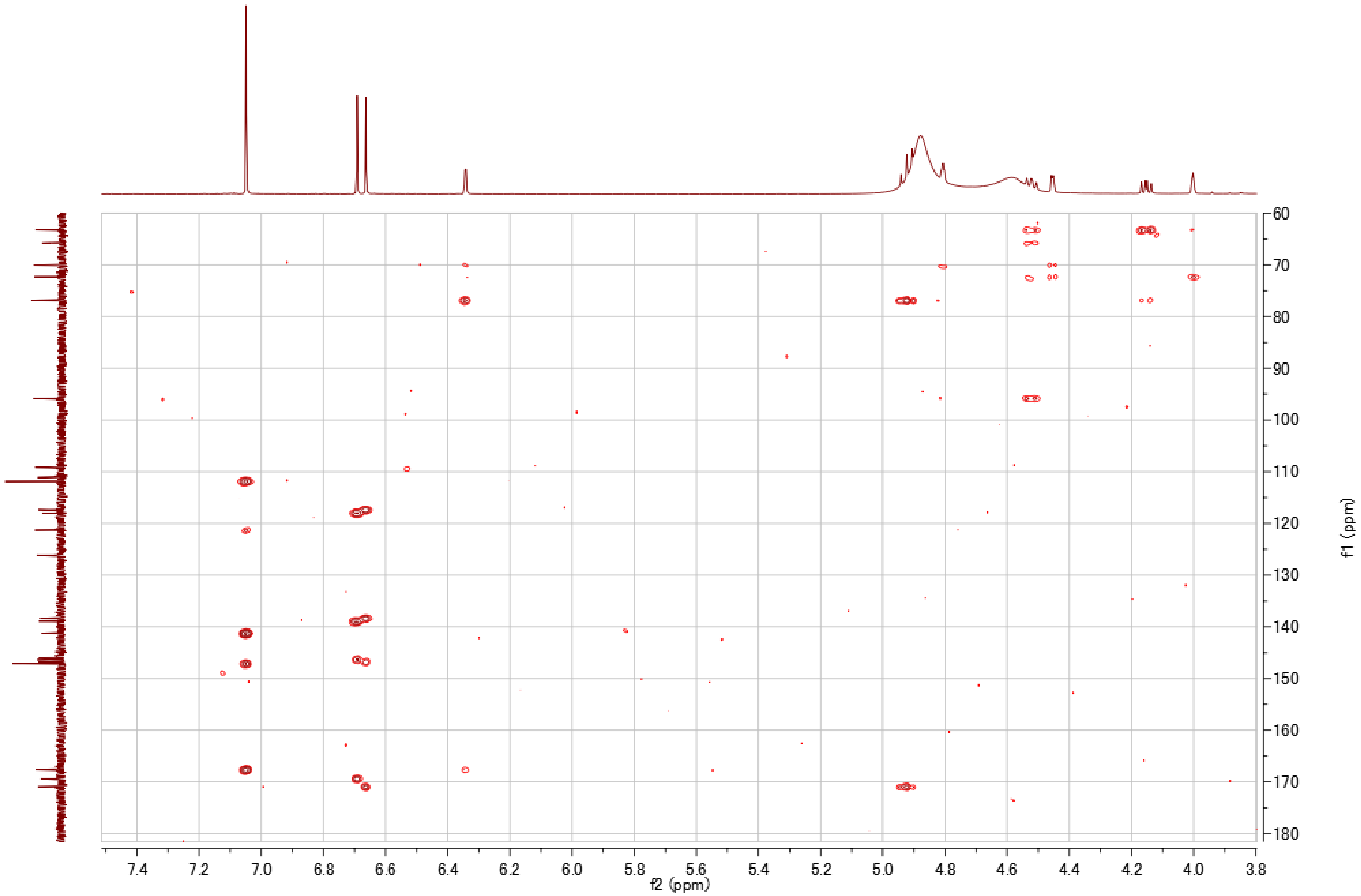


**Figure S24.** HMBC spectrum of compound **7** [acetone-$d_6$/$D_2O$, 9/1, *v/v*, 600 MHz]

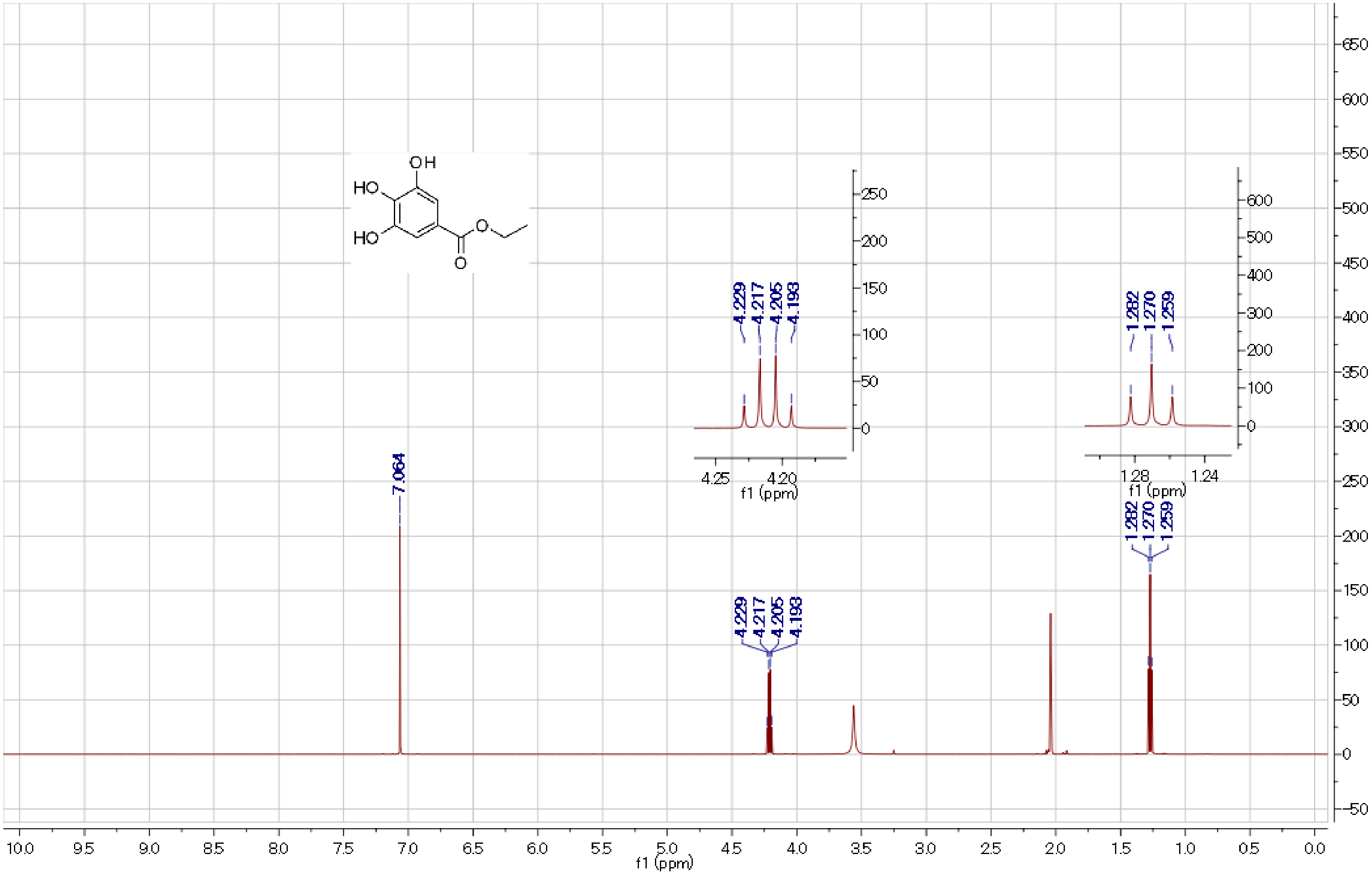


**Figure S25.** $^1$H NMR spectrum of compound **8** [acetone-$d_6$/$D_2O$, 9/1, *v*/*v*, 600 MHz]

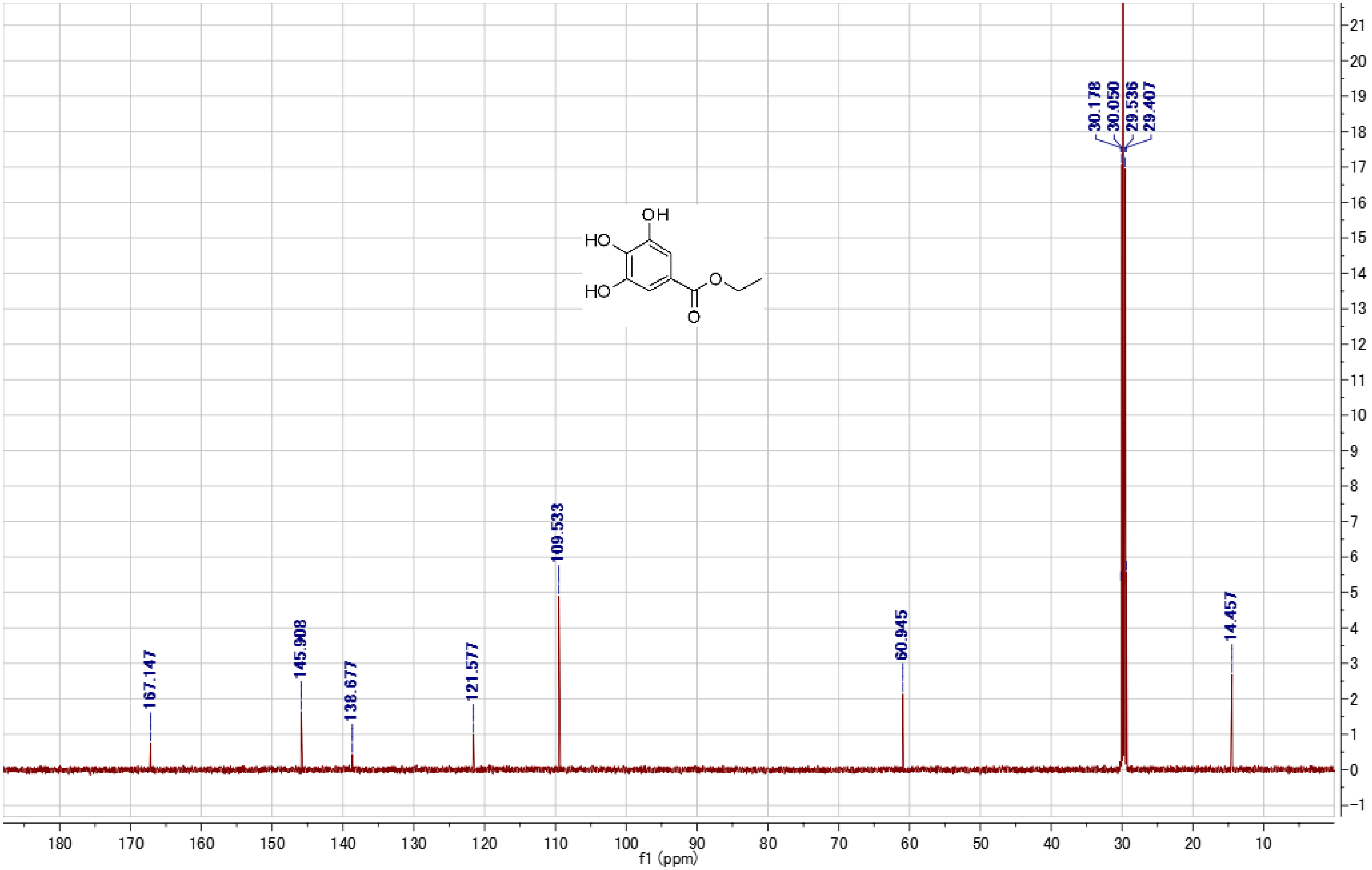


**Figure S26.** $^{13}C$ NMR spectrum of compound **8** [acetone-$d_6$/$D_2O$, 9/1, *v/v*, 151 MHz]

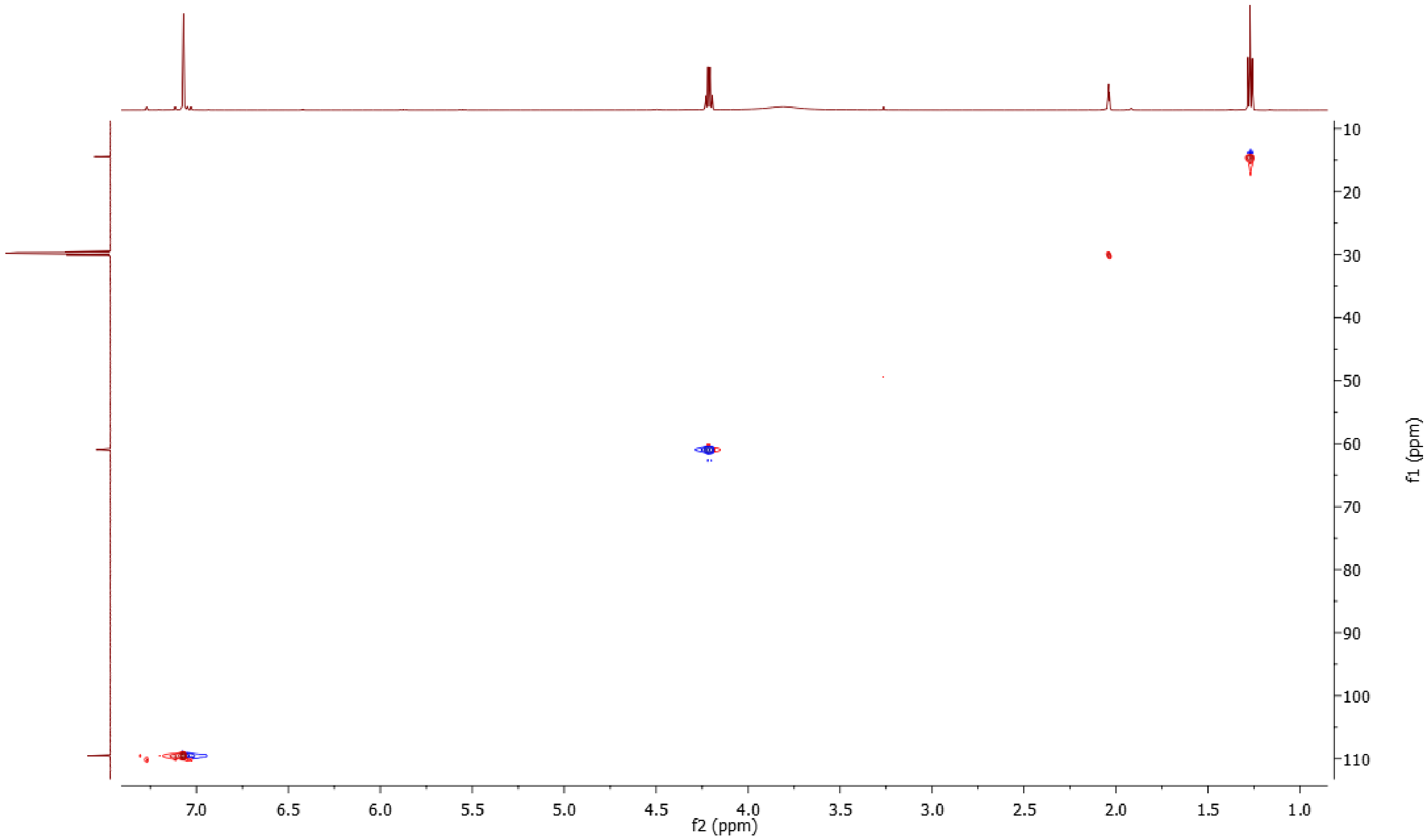


**Figure S27.** HSQC spectrum of compound **8** [acetone-$d_6$/$D_2O$, 9/1, *v/v*, 600 MHz]

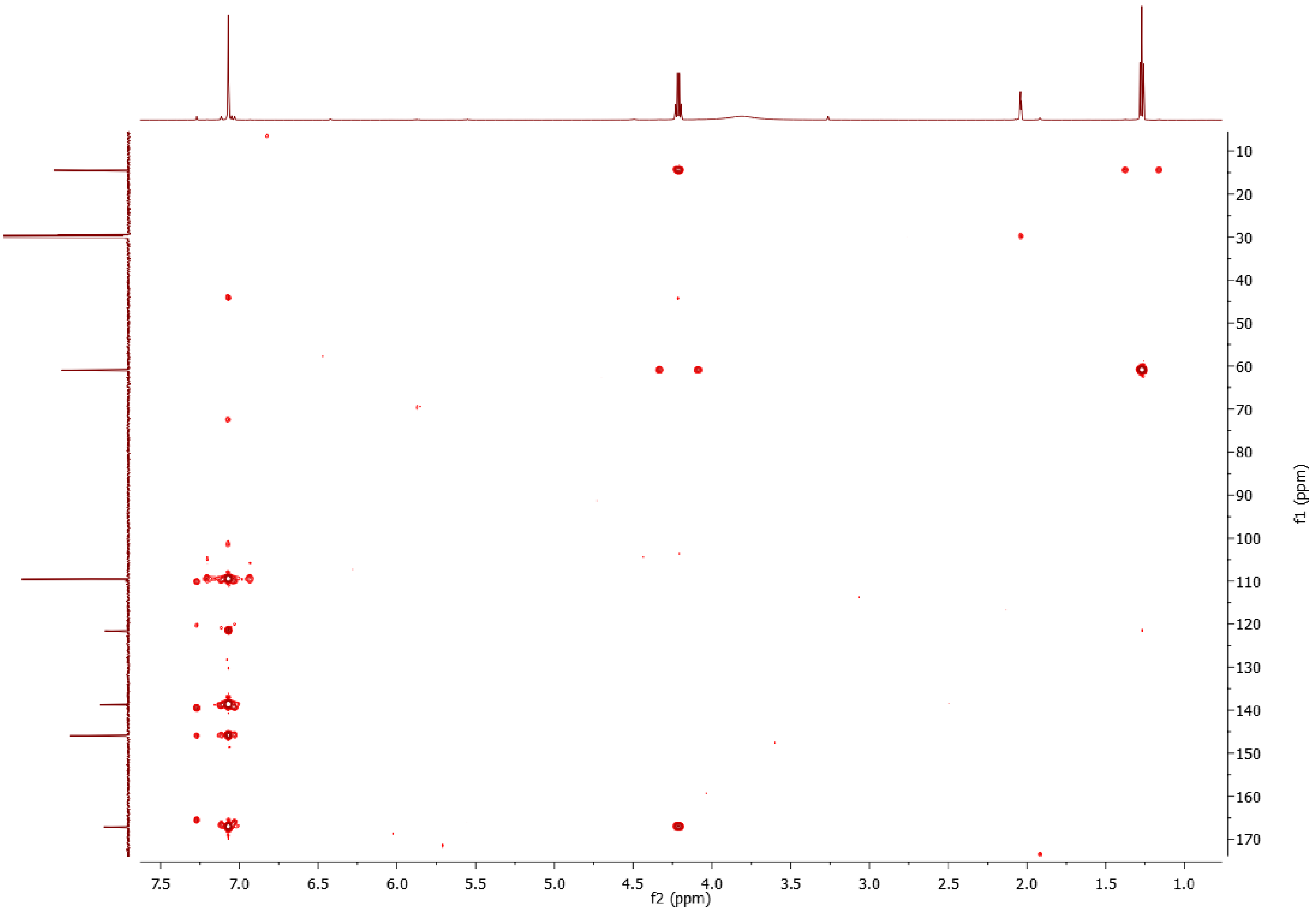


**Figure S28.** HMBC spectrum of compound **8** [acetone-$d_6$/$D_2O$, 9/1, *v/v*, 600 MHz]

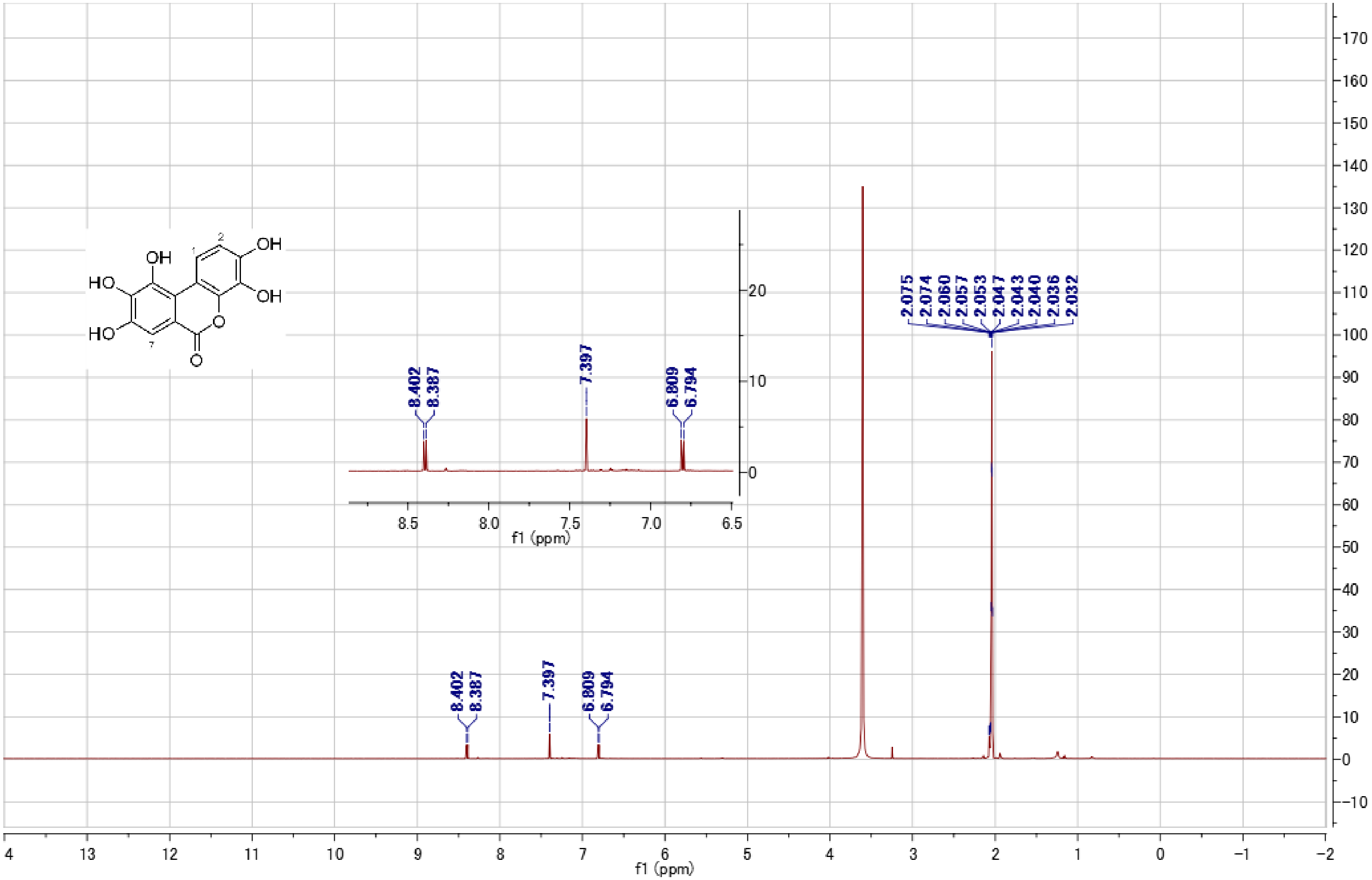


**Figure S29.** $^1$H NMR spectrum of compound **9** [acetone-$d_6$/$D_2O$, 9/1, *v/v*, 600 MHz]

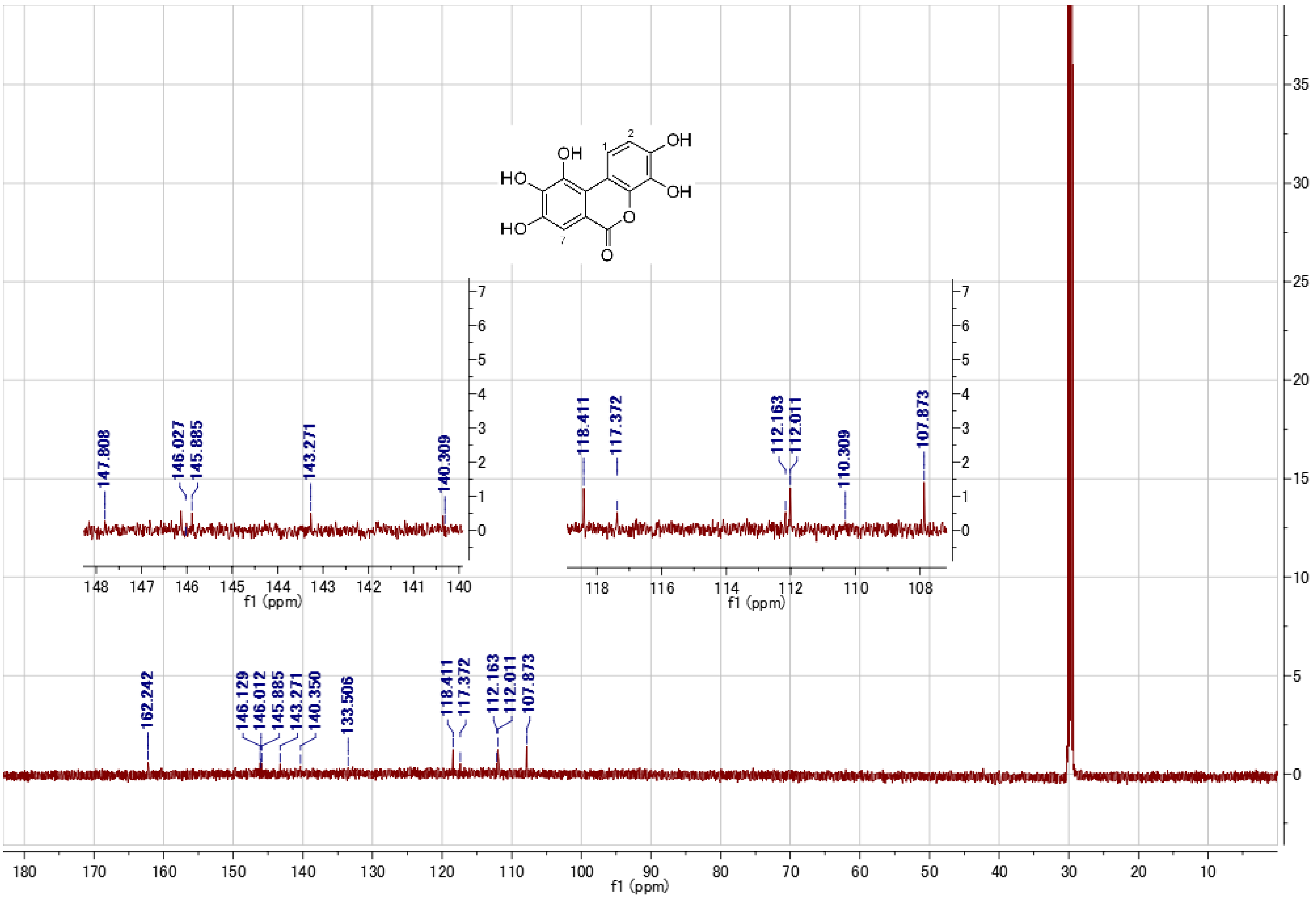

**Figure S30.** $^{13}C$ NMR spectrum of compound **9** [acetone-$d_6$/$D_2O$, 9/1, *v/v*, 151 MHz]

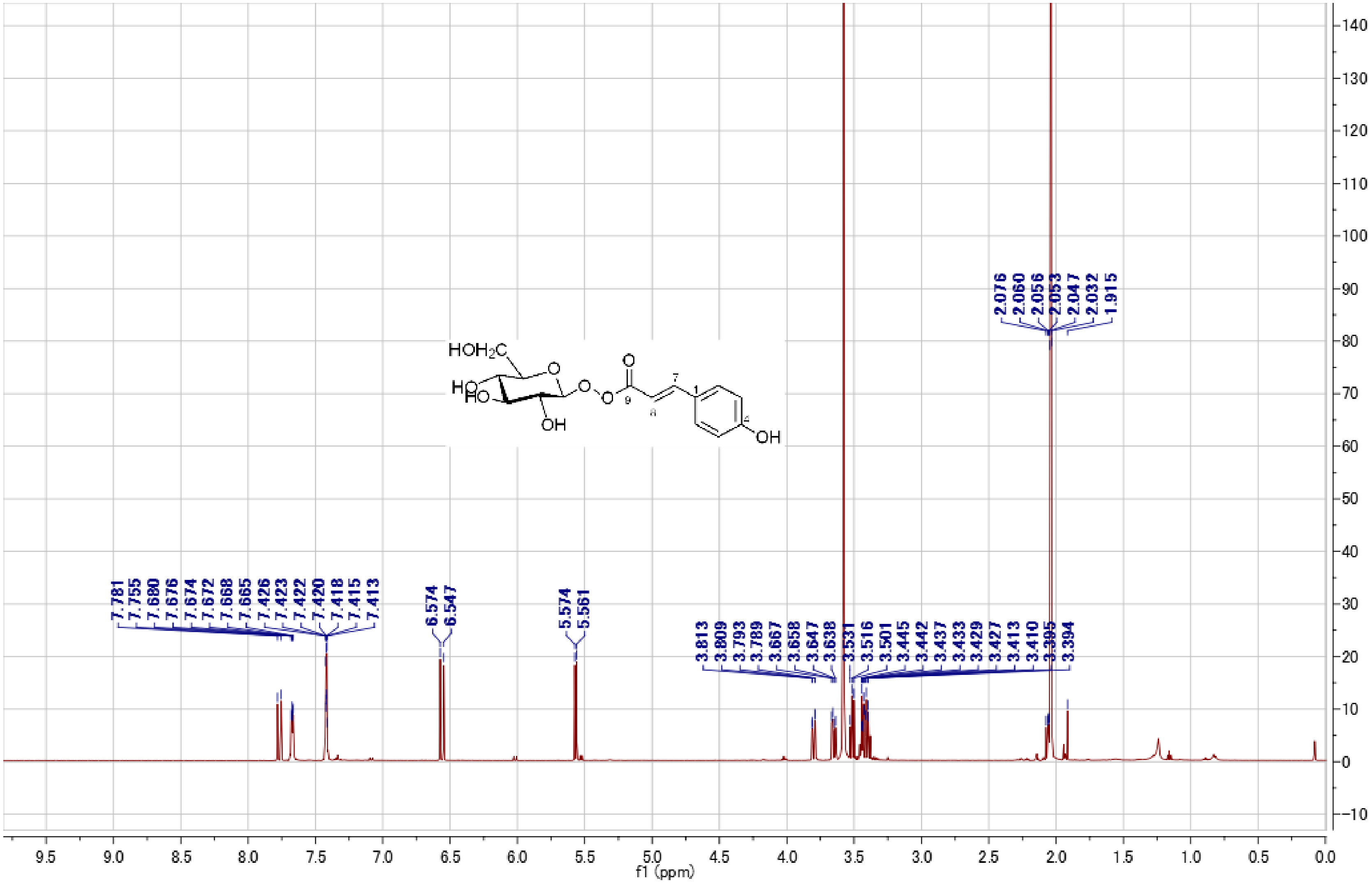

**Figure S31.** $^{1}$H NMR spectrum of compound **10** [acetone-$d_6$/$D_2O$, 9/1, *v/v*, 600 MHz]

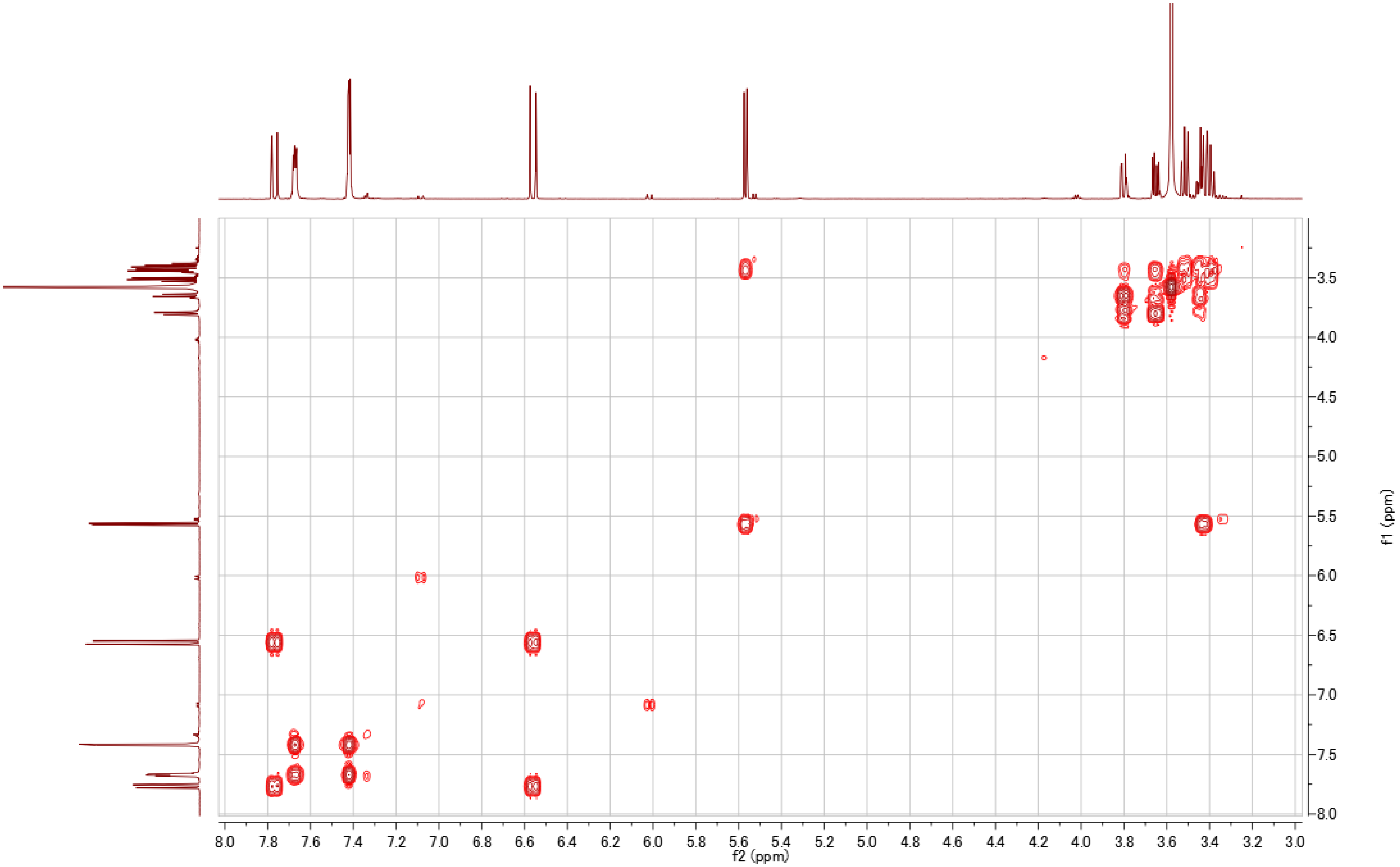


**Figure S32.** $^{1}$H–$^{1}$H COSY spectrum of compound **10** [acetone-$d_6$/$D_2O$, 9/1, *v/v*, 600 MHz]

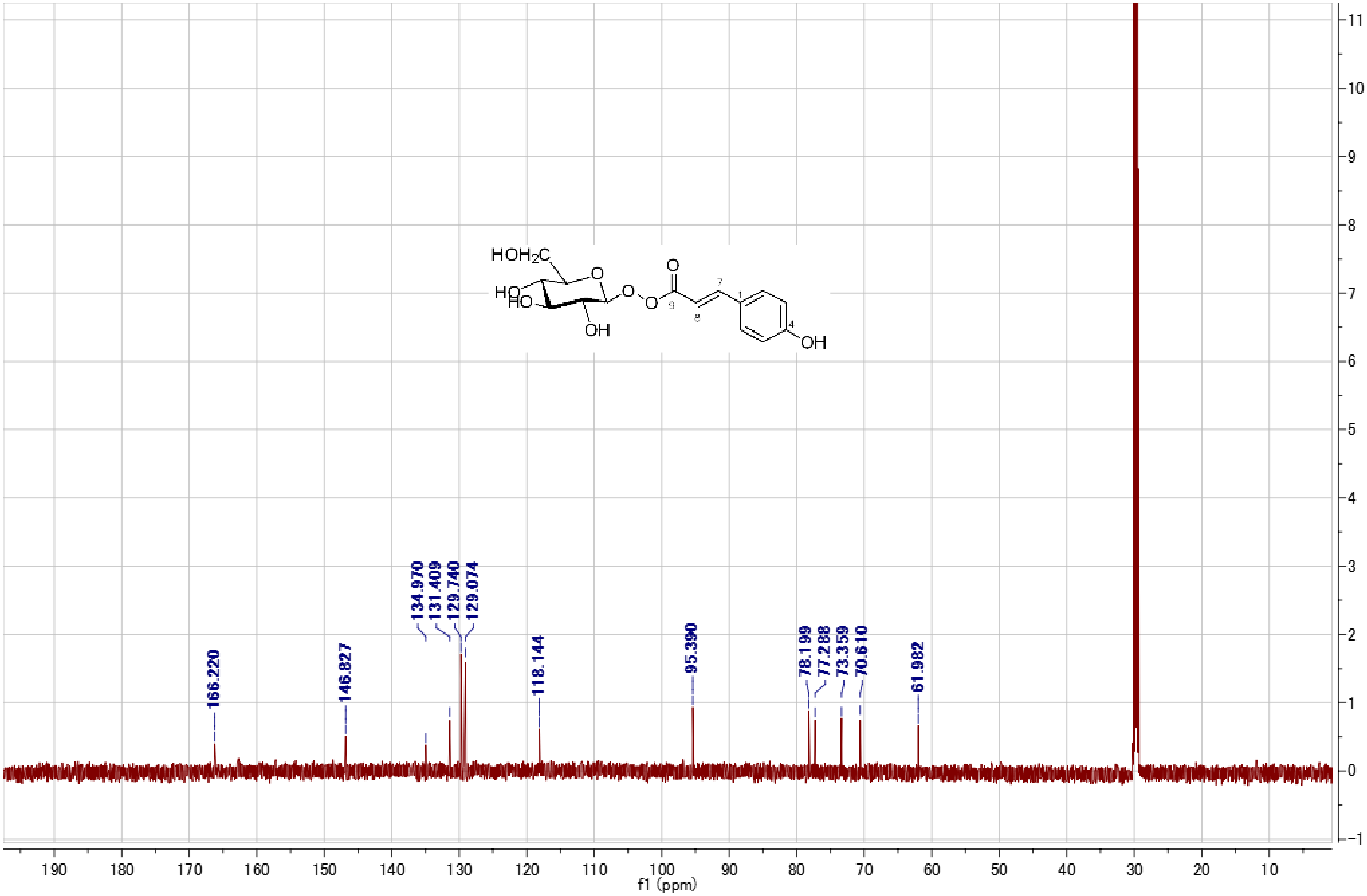


**Figure S33.** $^{13}$C NMR spectrum of compound **10** [acetone-$d_6$/$D_2O$, 9/1, *v/v*, 151 MHz]

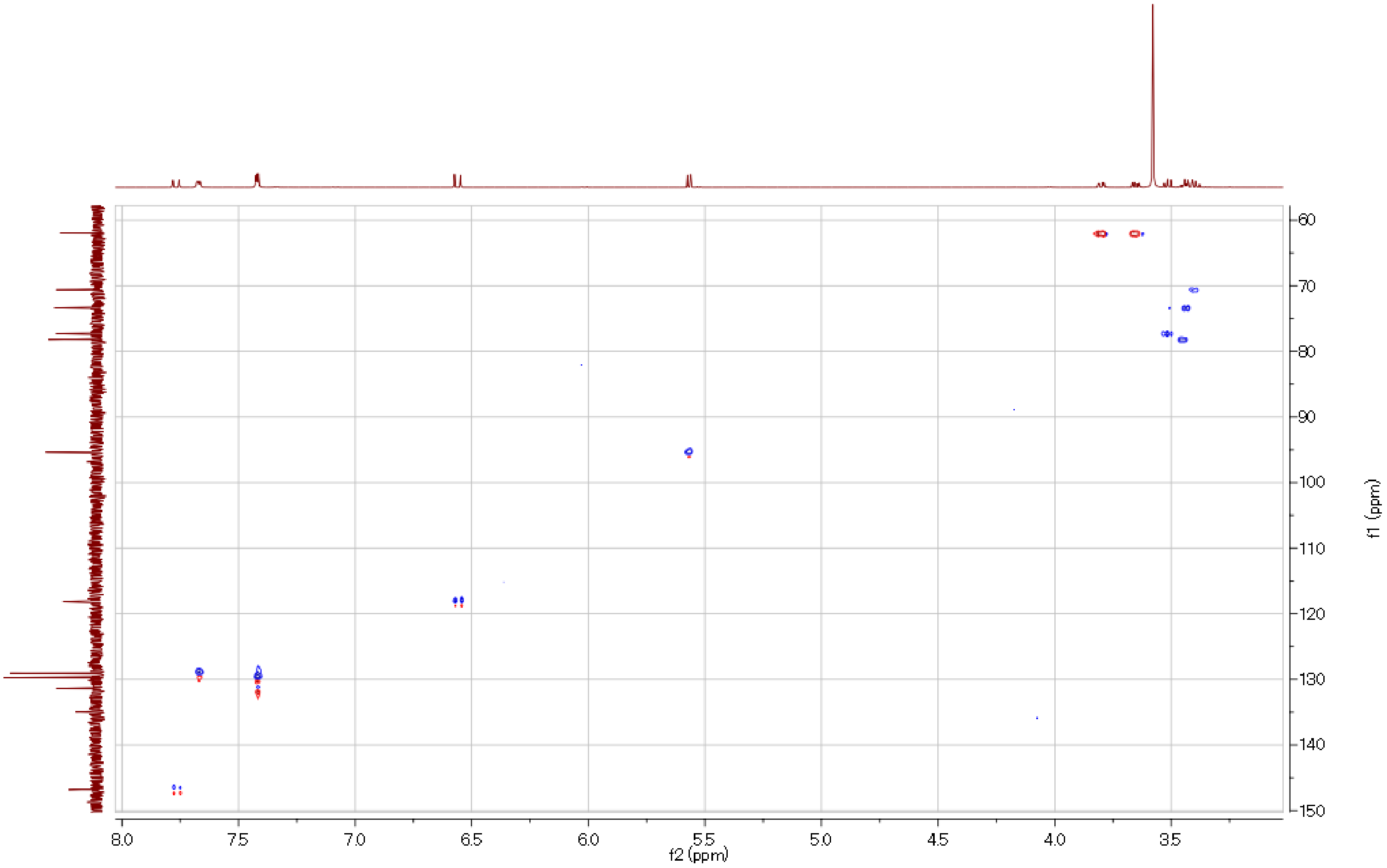


**Figure S34.** HSQC spectrum of compound **10** [acetone-$d_6$/$D_2O$, 9/1, *v*/*v*, 600 MHz]

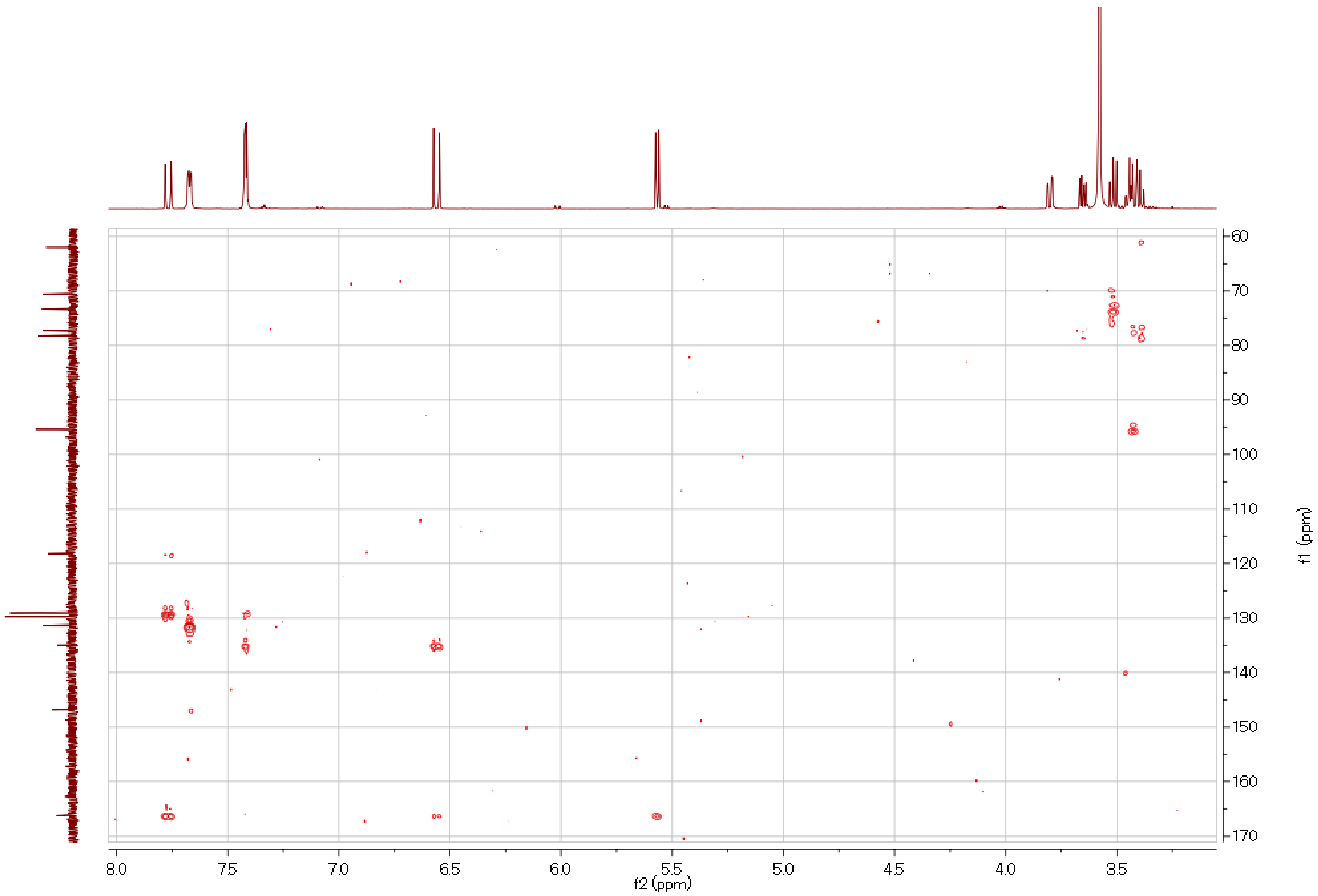


**Figure S35.** HMBC spectrum of compound **10** [acetone-$d_6$/$D_2O$, 9/1, *v/v*, 600 MHz]

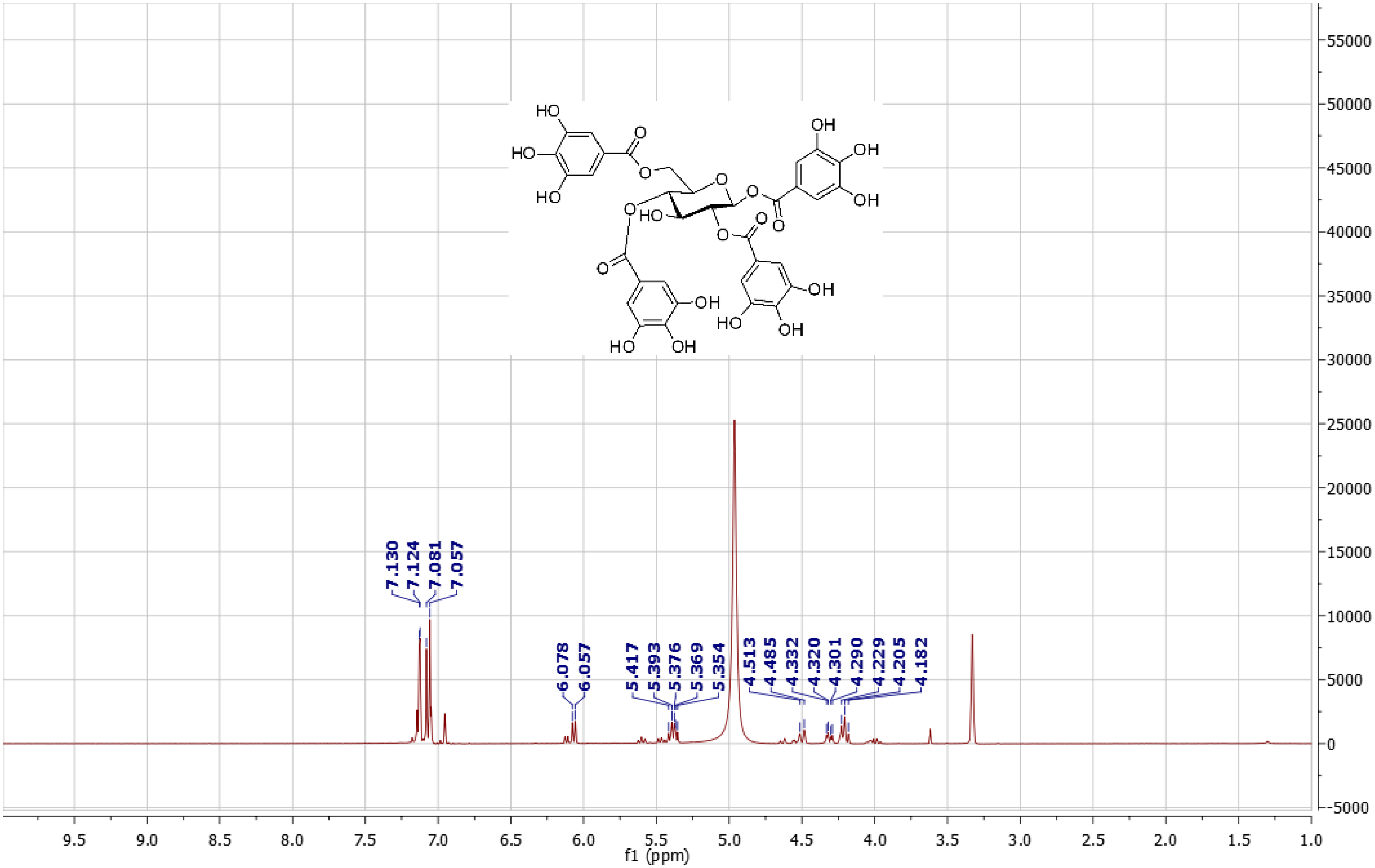


**Figure S36.** $^{1}$H NMR spectrum of compound **11** ($CD_3OD$, 100 MHz)

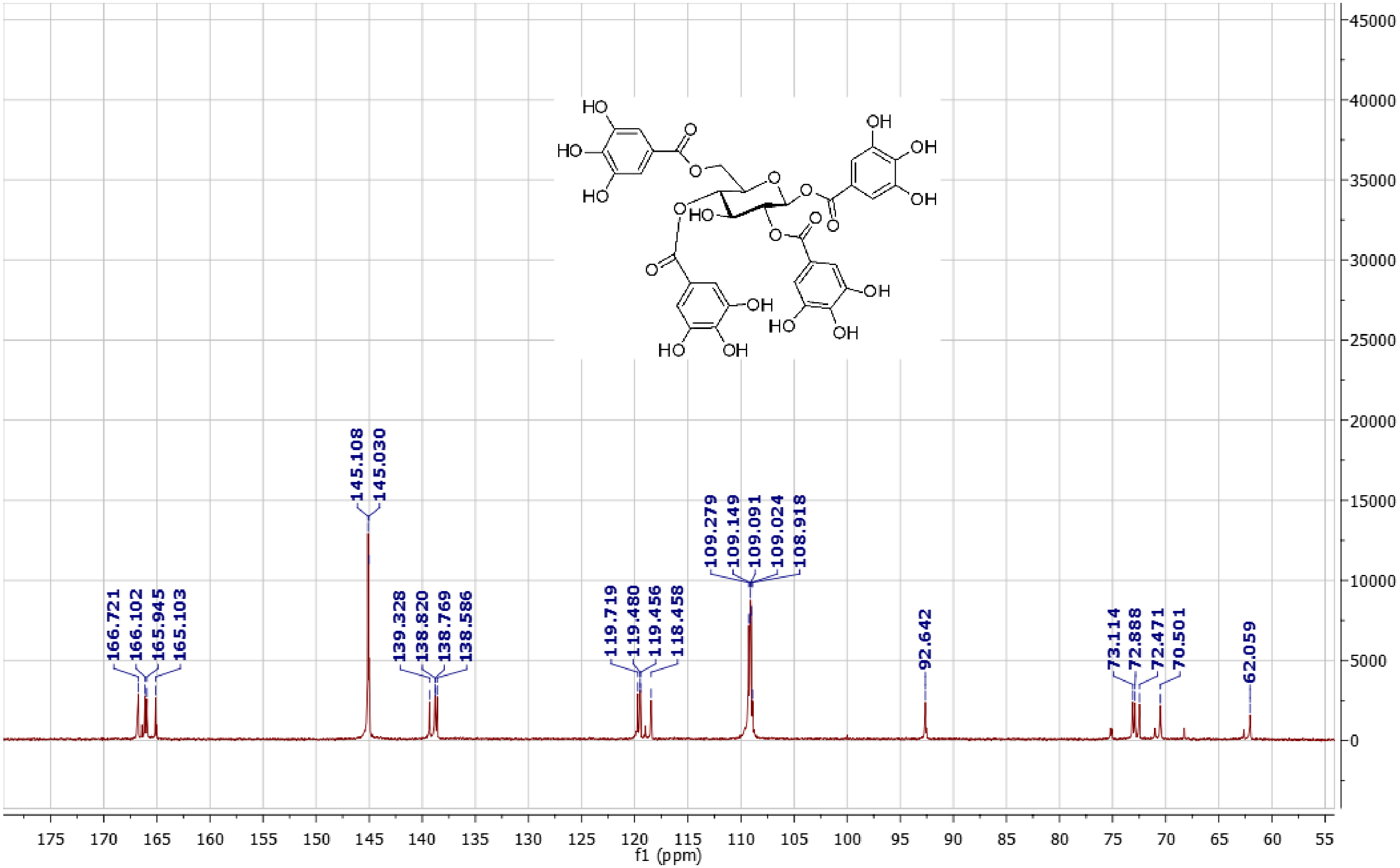


**Figure S37.** $^{13}$C NMR spectrum of compound **11** ($CD_3OD$, 100 MHz)
